# Supplementary figures and images for: Combinatorial expression of γ-protocadherins regulates synaptic connectivity in the mouse neocortex
Source: eLife. 2024 Mar 12;12:RP89532. doi: 10.7554/eLife.89532 (PMC10932546; doi:10.7554/eLife.89532)

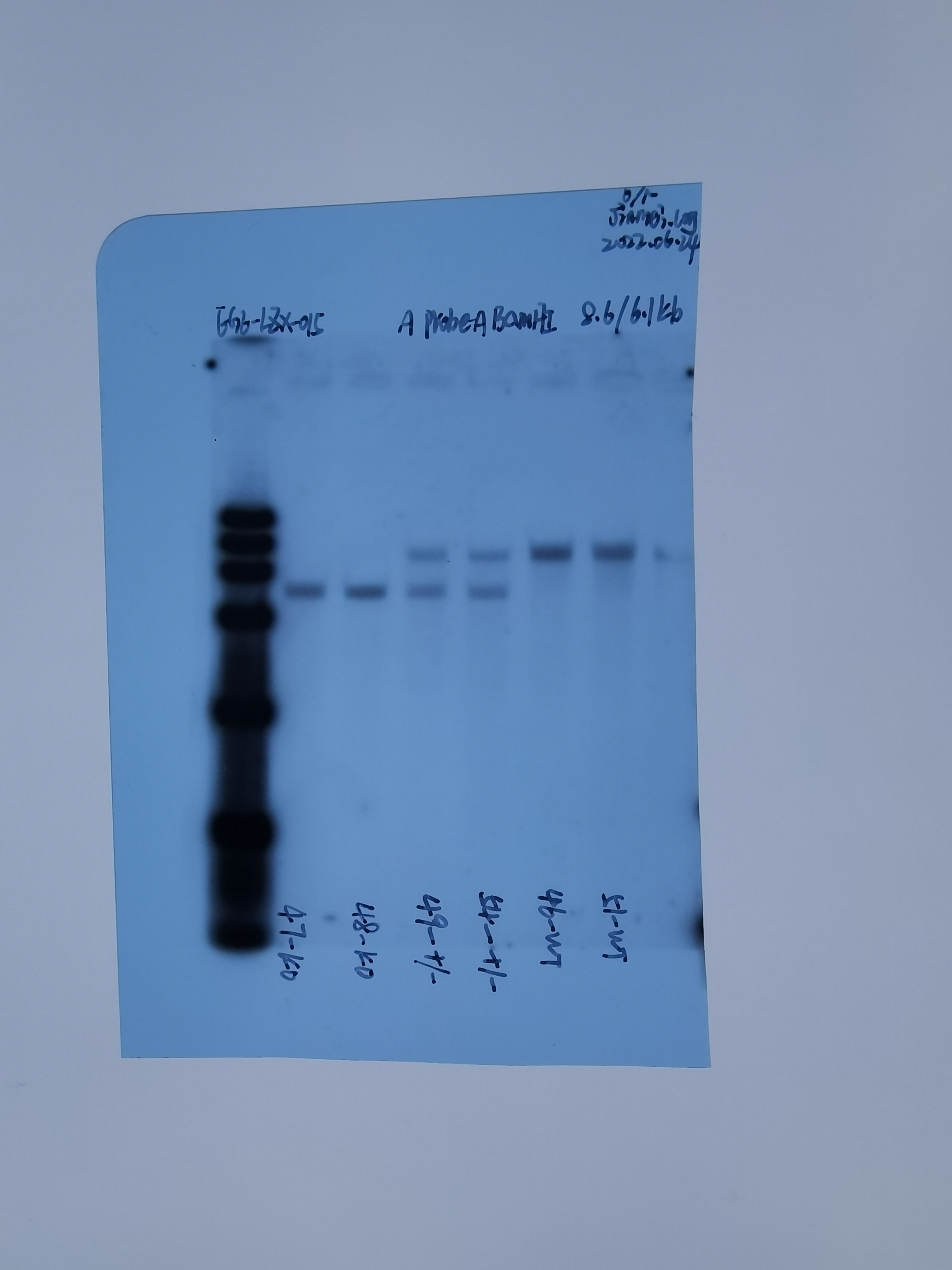

Supplement: Figure 2—figure supplement 2—source data 1. [file elife-89532-fig2-figsupp2-data1.zip › Figure 2-Figure Supplement2-Source Data1/Fig.2-S2C_1.jpg]

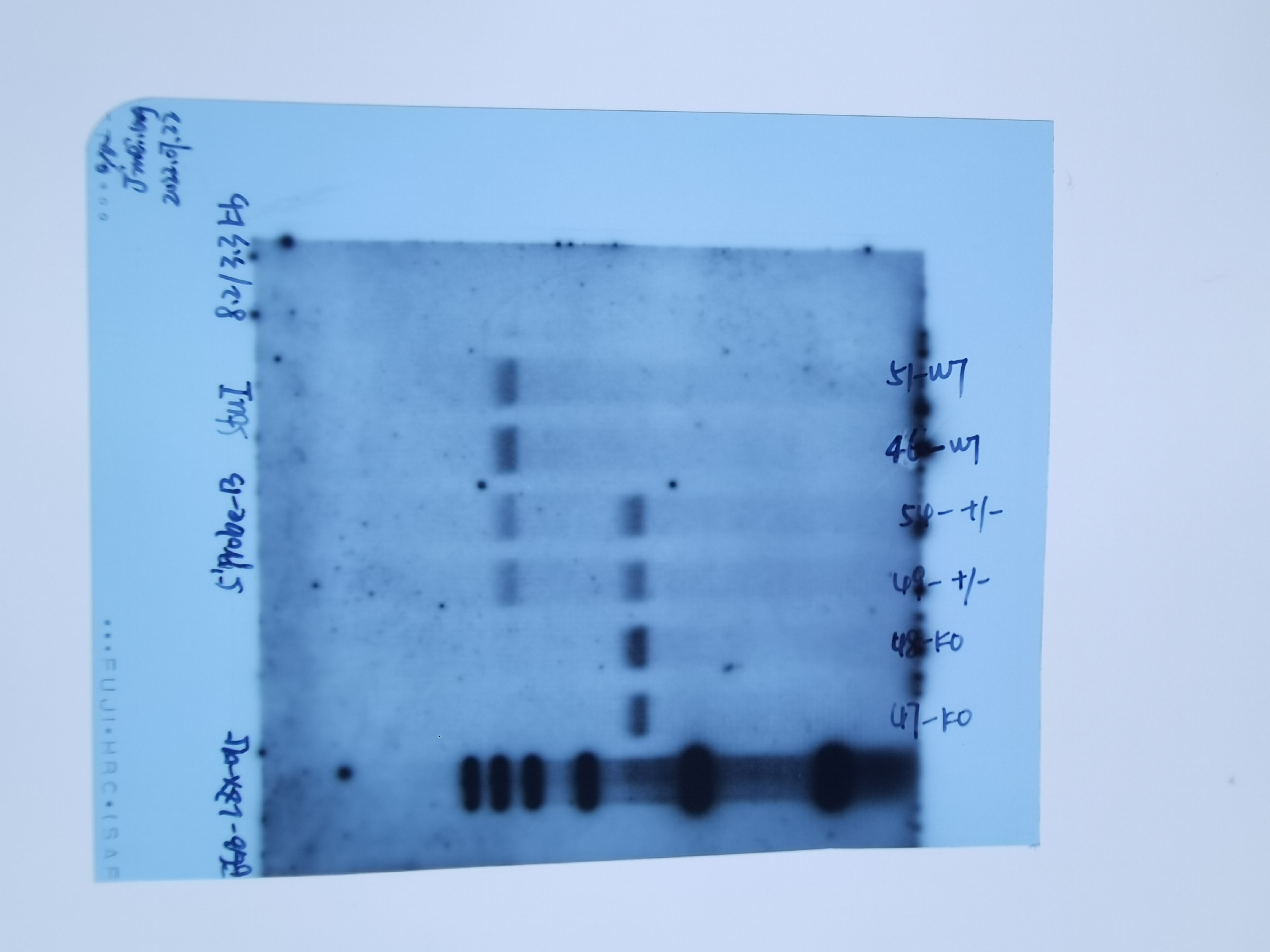

Supplement: Figure 2—figure supplement 2—source data 1. [file elife-89532-fig2-figsupp2-data1.zip › Figure 2-Figure Supplement2-Source Data1/Fig.2-S2C_2.jpg]

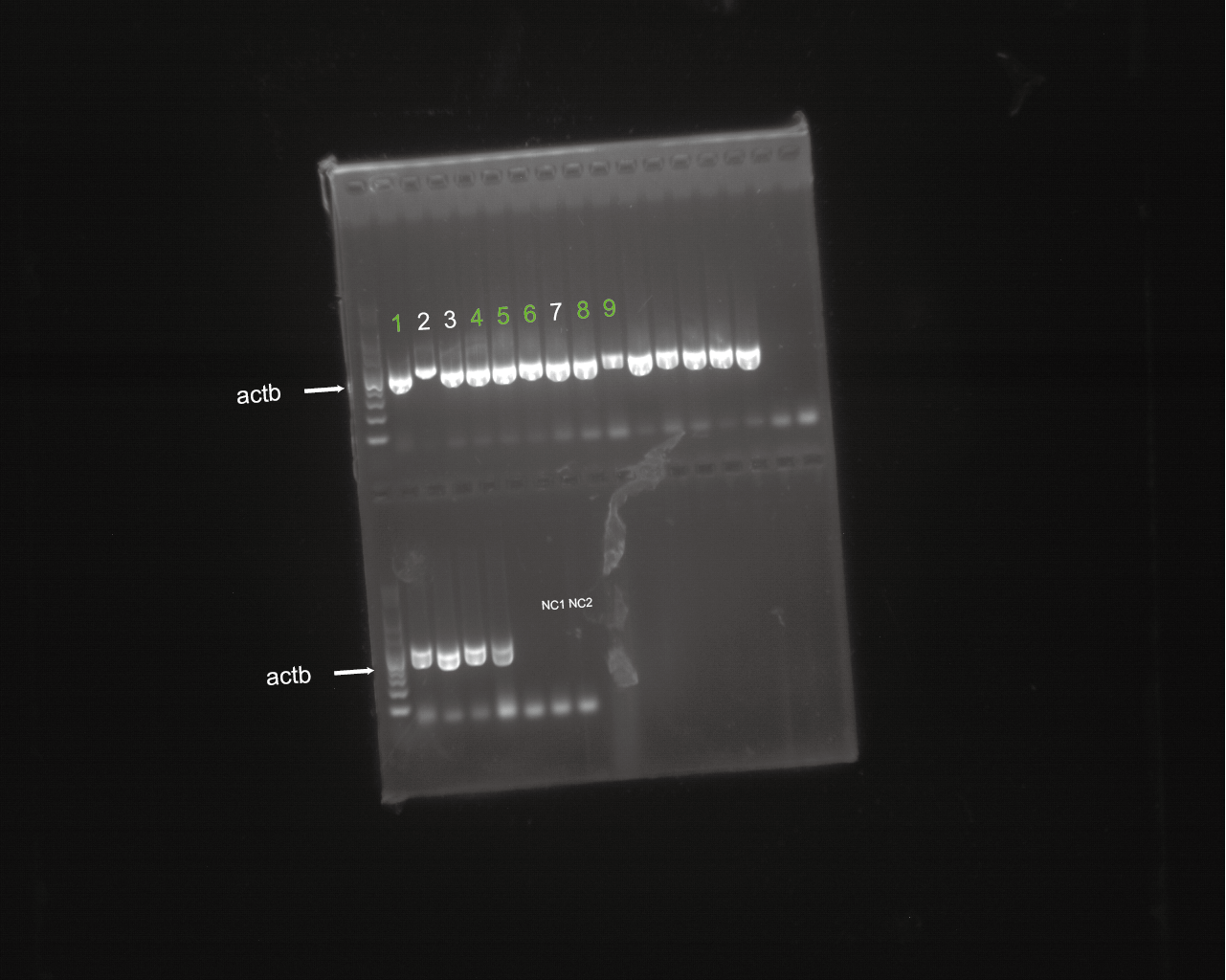

Supplement: Figure 3—source data 2. [file elife-89532-fig3-data2.zip › Figure 3-Source Data 2/E1/labelled image/actin confirm .tif]

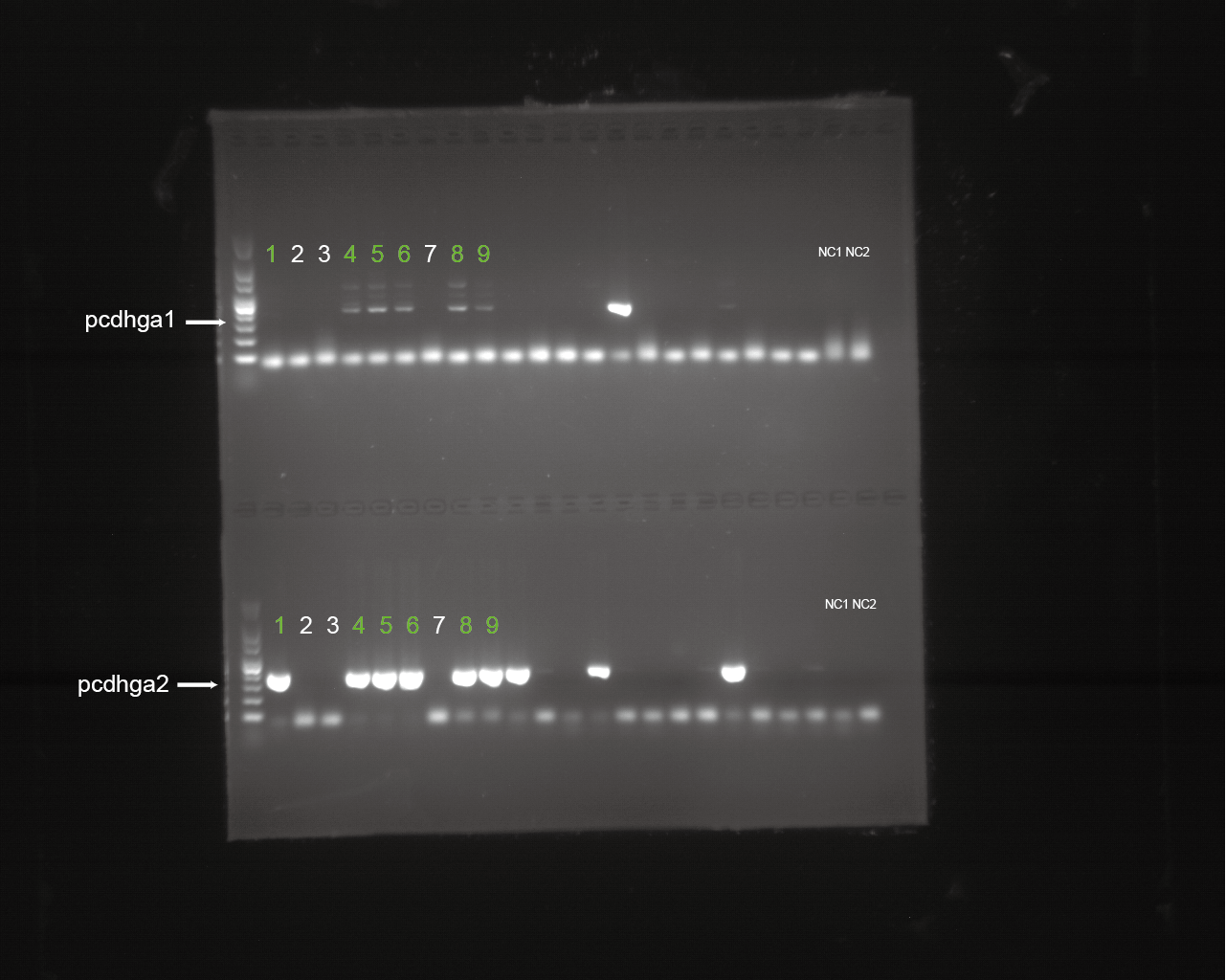

Supplement: Figure 3—source data 2. [file elife-89532-fig3-data2.zip › Figure 3-Source Data 2/E1/labelled image/ga1 g2 .tif]

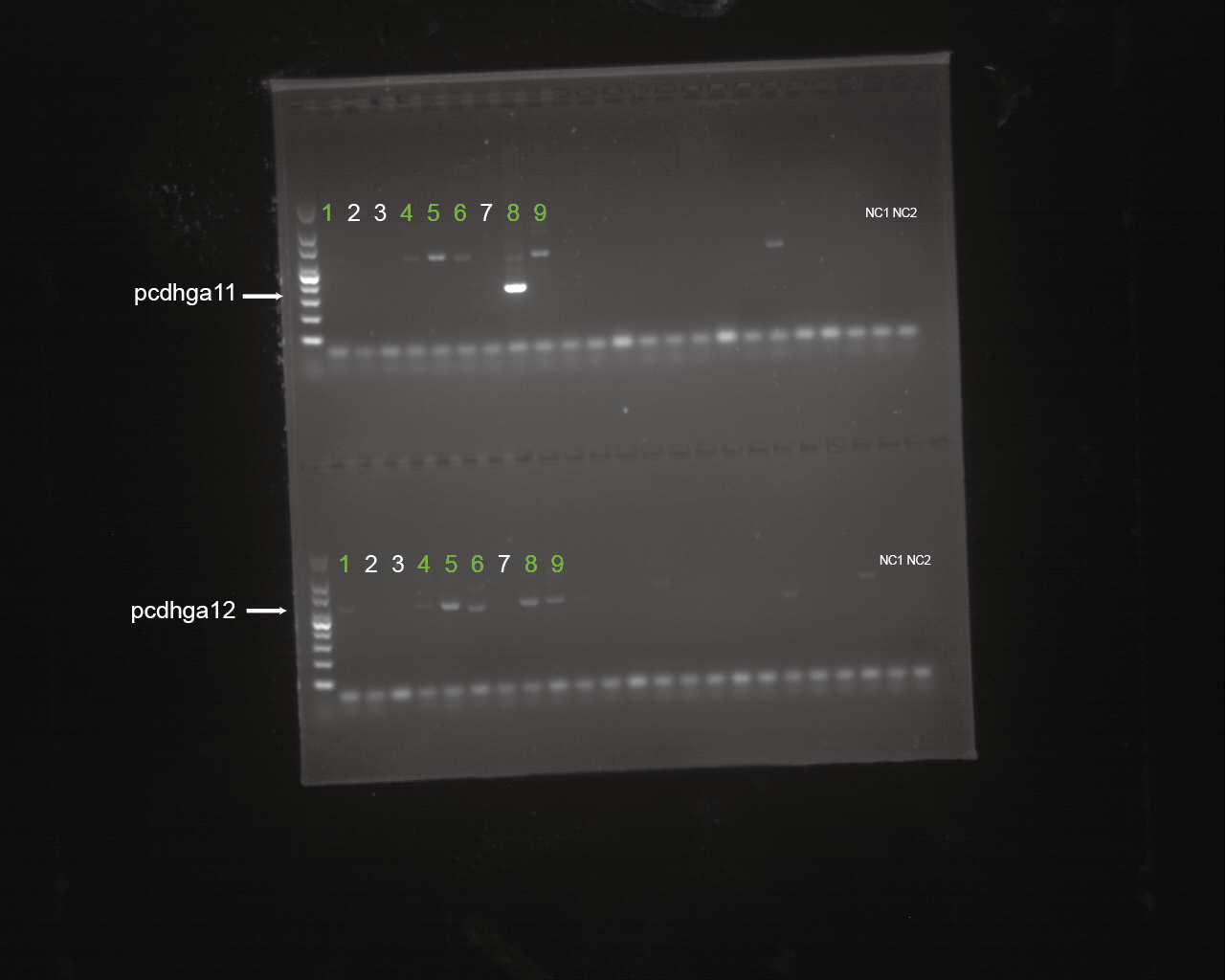

Supplement: Figure 3—source data 2. [file elife-89532-fig3-data2.zip › Figure 3-Source Data 2/E1/labelled image/ga11,ga12 .tif]

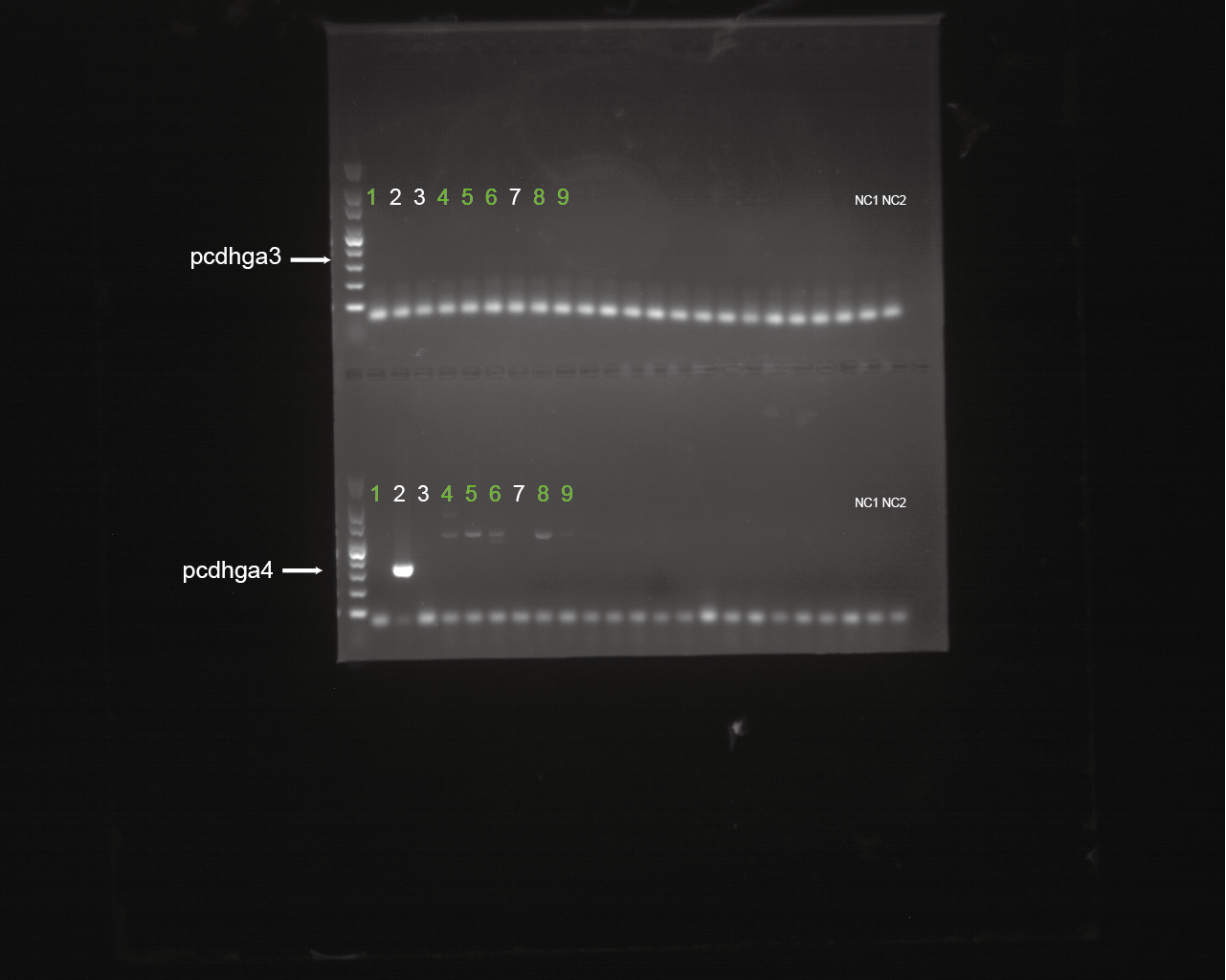

Supplement: Figure 3—source data 2. [file elife-89532-fig3-data2.zip › Figure 3-Source Data 2/E1/labelled image/ga3,ga4 .tif]

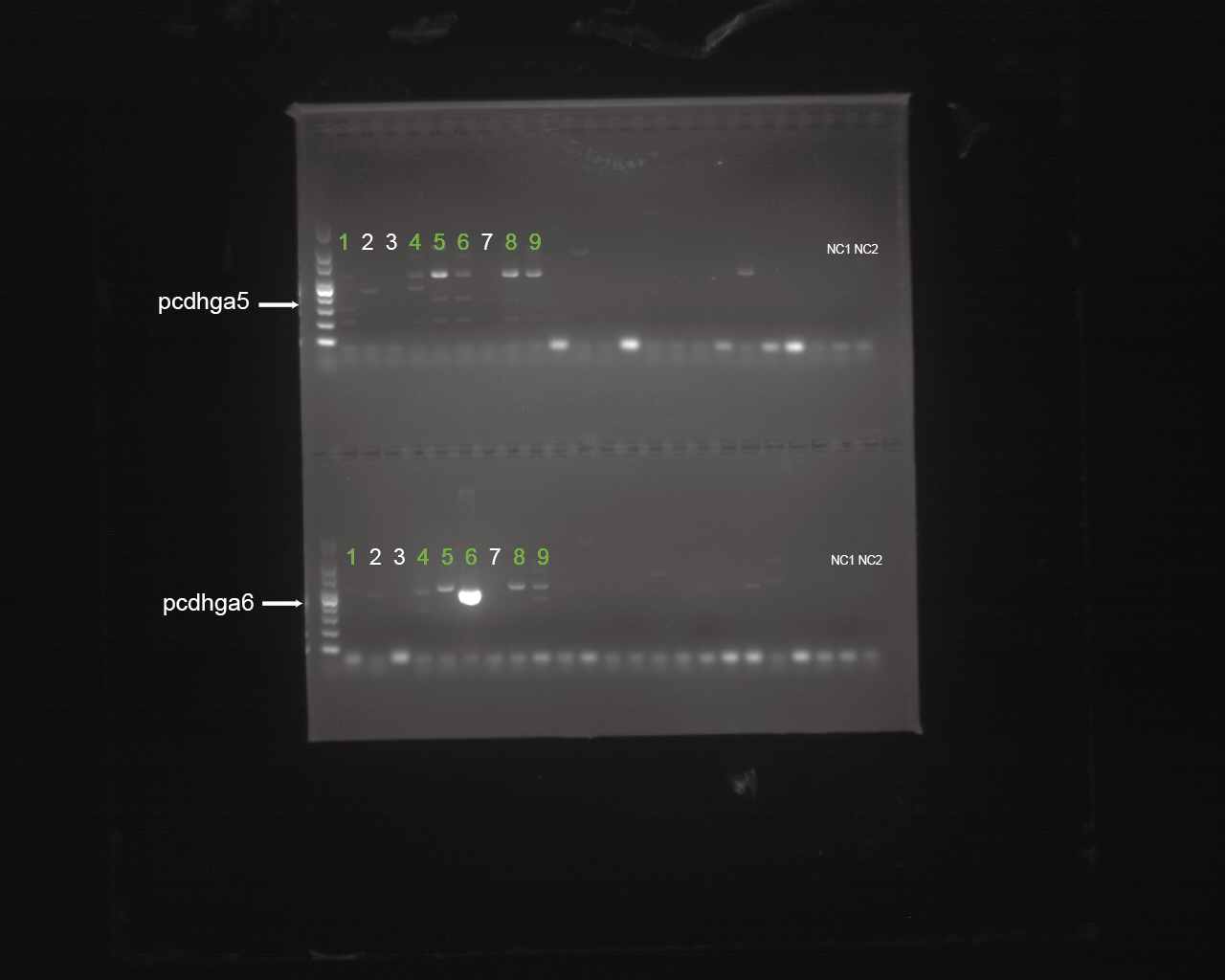

Supplement: Figure 3—source data 2. [file elife-89532-fig3-data2.zip › Figure 3-Source Data 2/E1/labelled image/ga5,ga6 .tif]

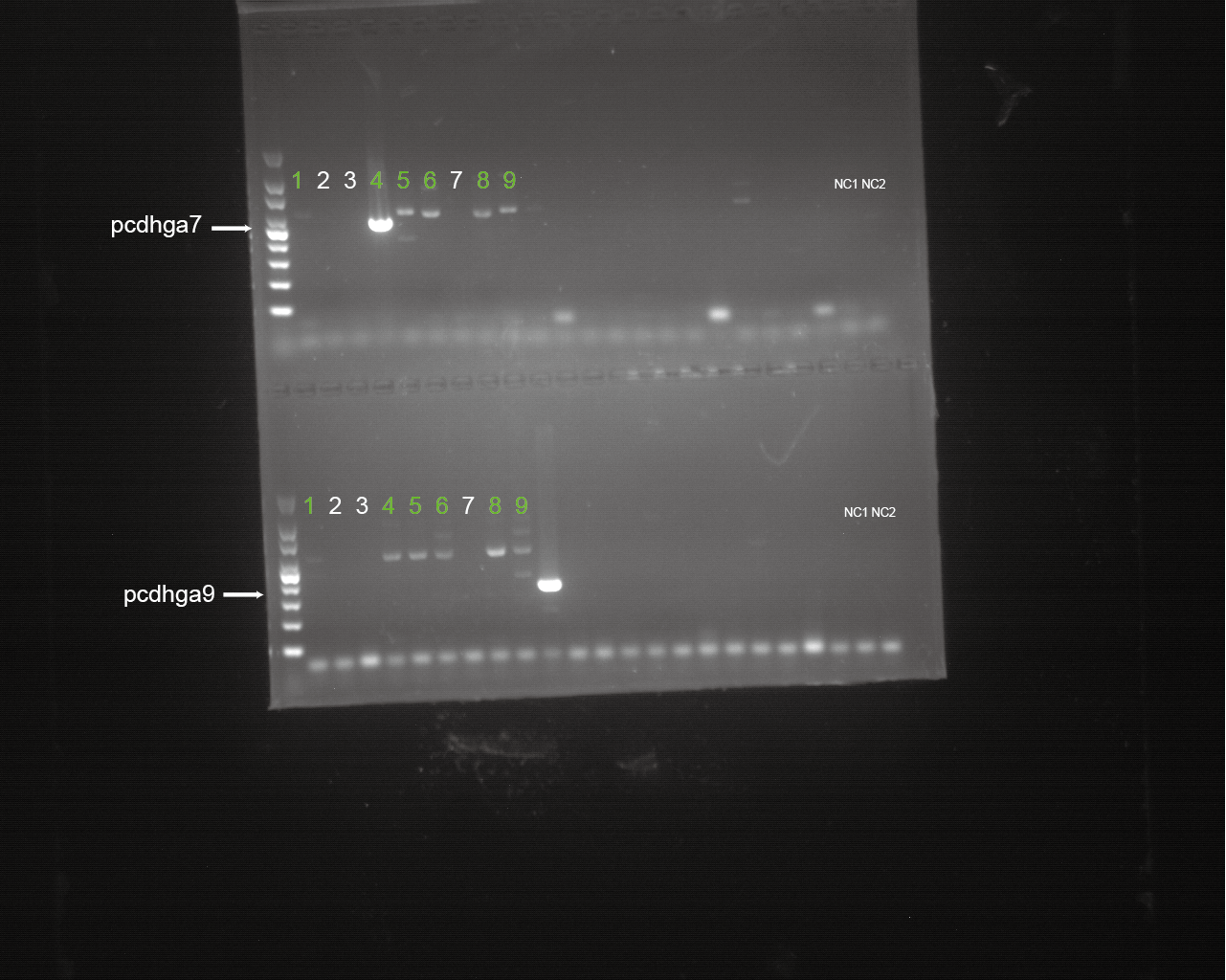

Supplement: Figure 3—source data 2. [file elife-89532-fig3-data2.zip › Figure 3-Source Data 2/E1/labelled image/ga7,ga9 .tif]

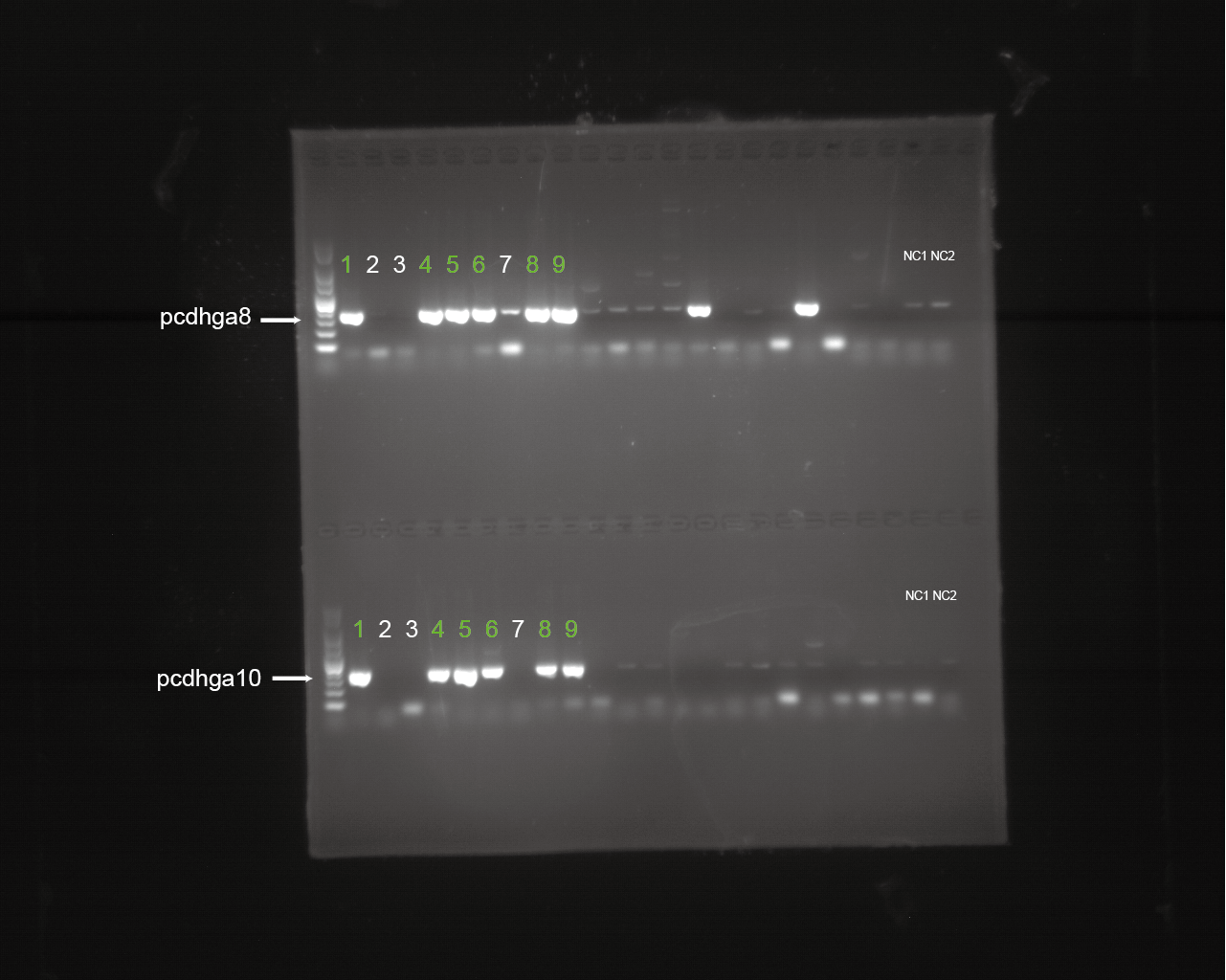

Supplement: Figure 3—source data 2. [file elife-89532-fig3-data2.zip › Figure 3-Source Data 2/E1/labelled image/ga8 g10 .tif]

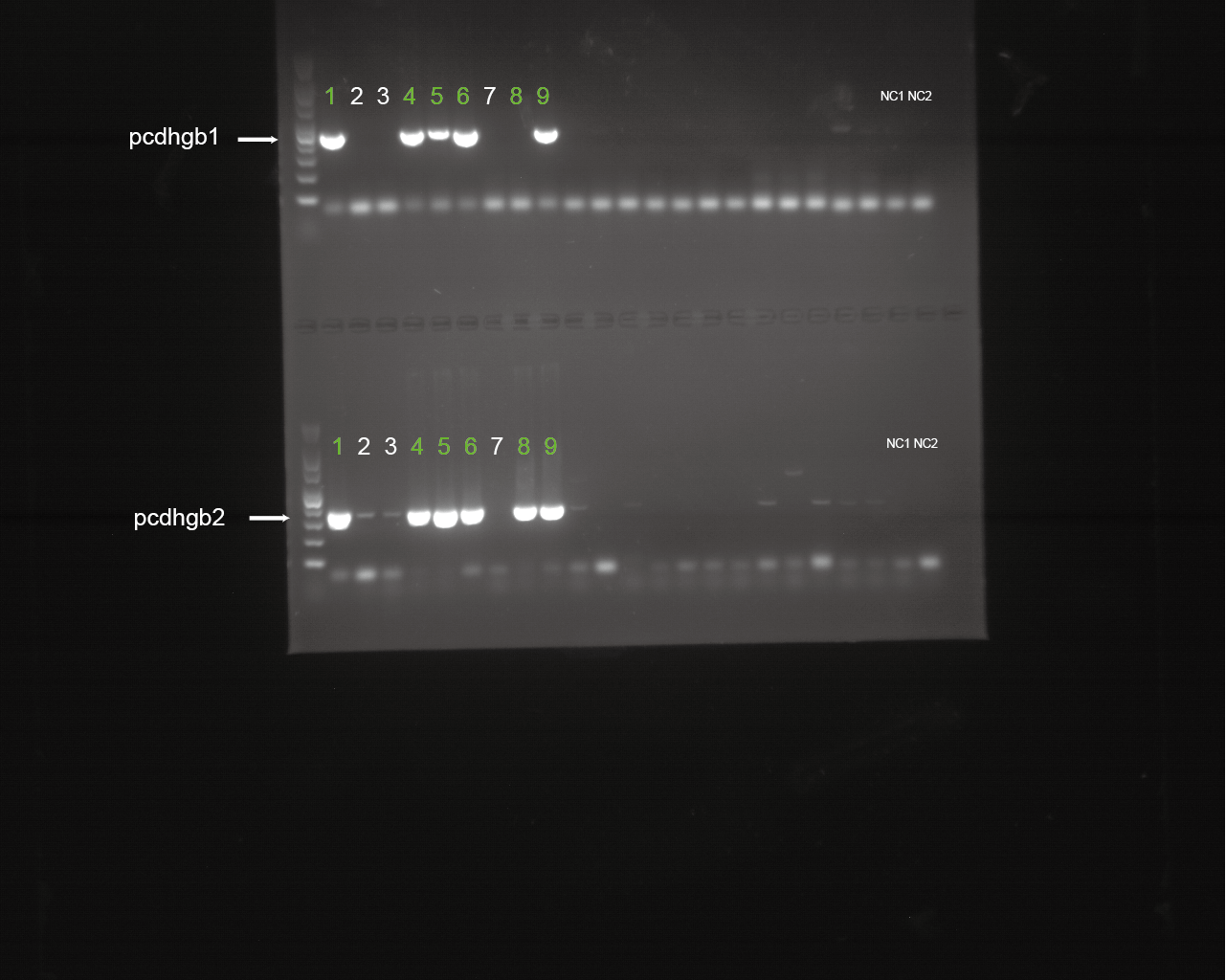

Supplement: Figure 3—source data 2. [file elife-89532-fig3-data2.zip › Figure 3-Source Data 2/E1/labelled image/gb1 gb2 .tif]

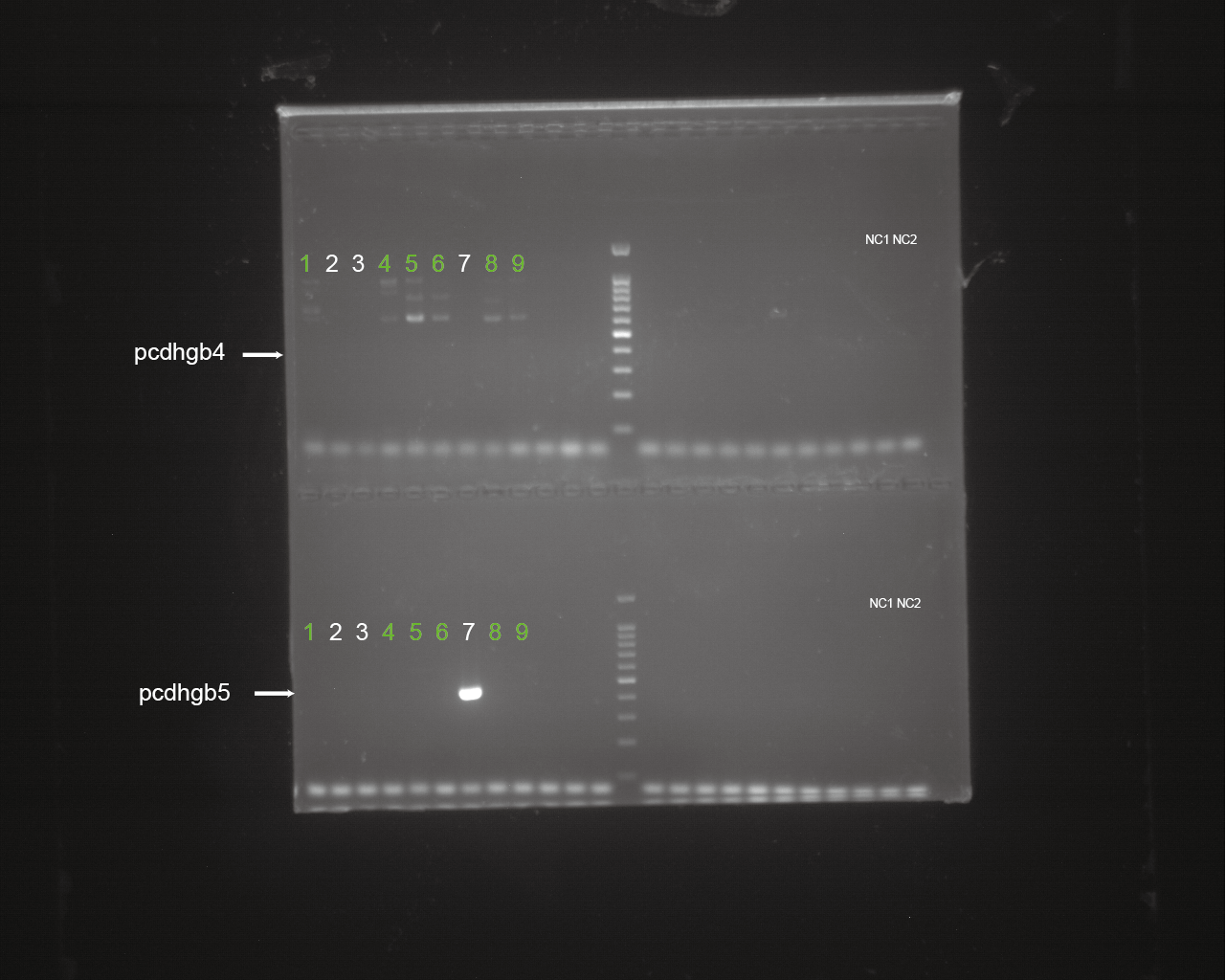

Supplement: Figure 3—source data 2. [file elife-89532-fig3-data2.zip › Figure 3-Source Data 2/E1/labelled image/gb4,gb5 .tif]

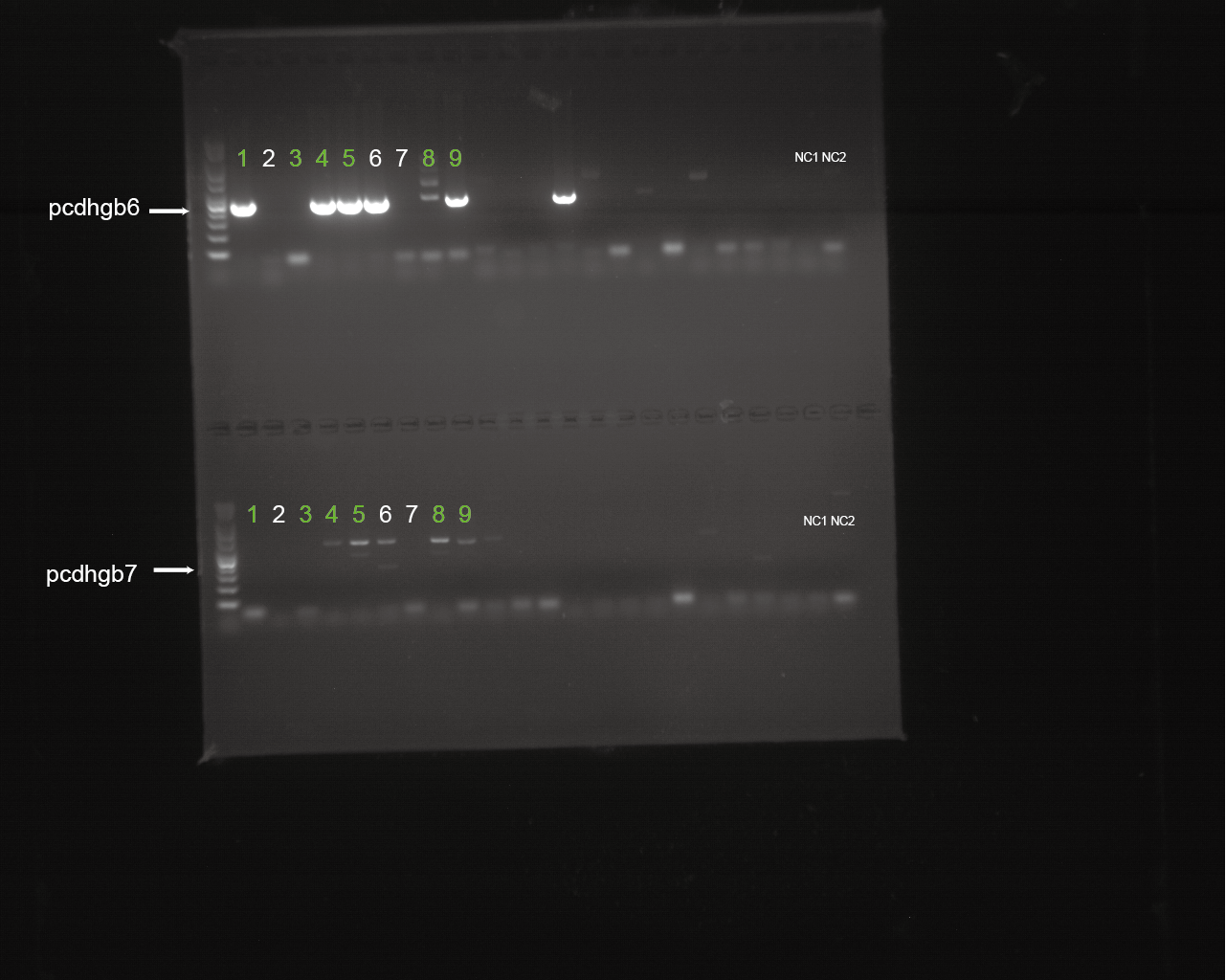

Supplement: Figure 3—source data 2. [file elife-89532-fig3-data2.zip › Figure 3-Source Data 2/E1/labelled image/gb6 gb7.tif]

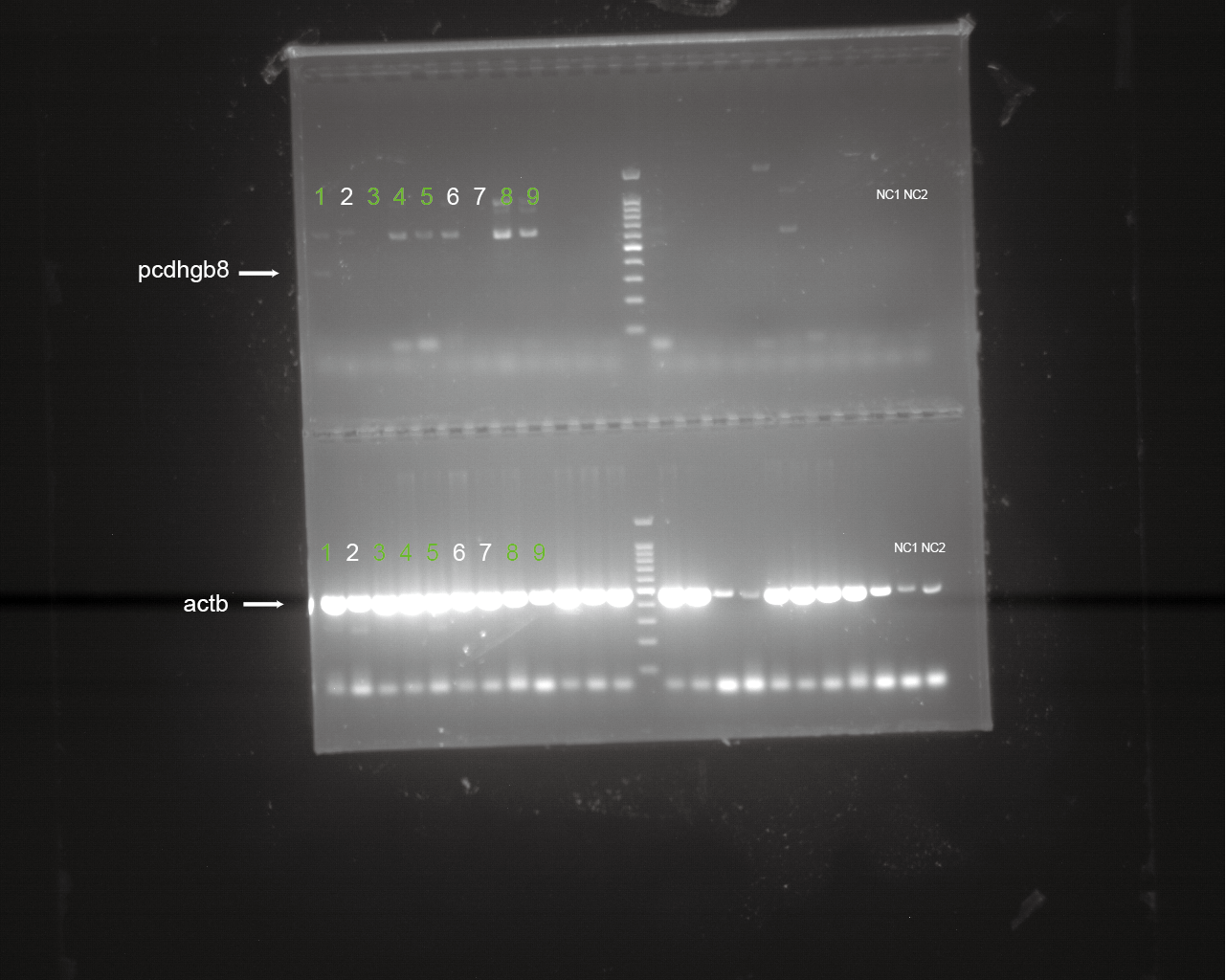

Supplement: Figure 3—source data 2. [file elife-89532-fig3-data2.zip › Figure 3-Source Data 2/E1/labelled image/gb8,actb (2).tif]

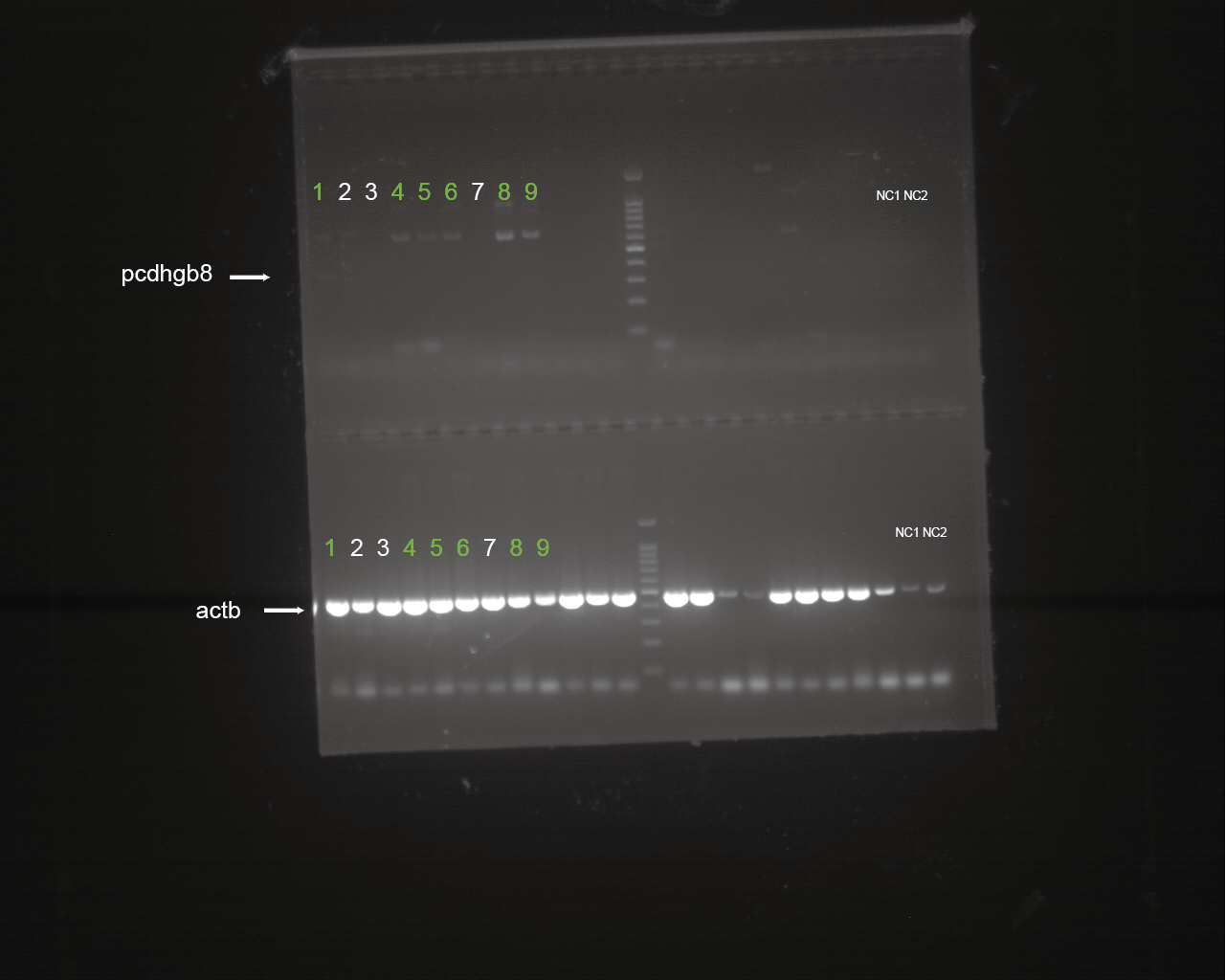

Supplement: Figure 3—source data 2. [file elife-89532-fig3-data2.zip › Figure 3-Source Data 2/E1/labelled image/gb8,actb .tif]

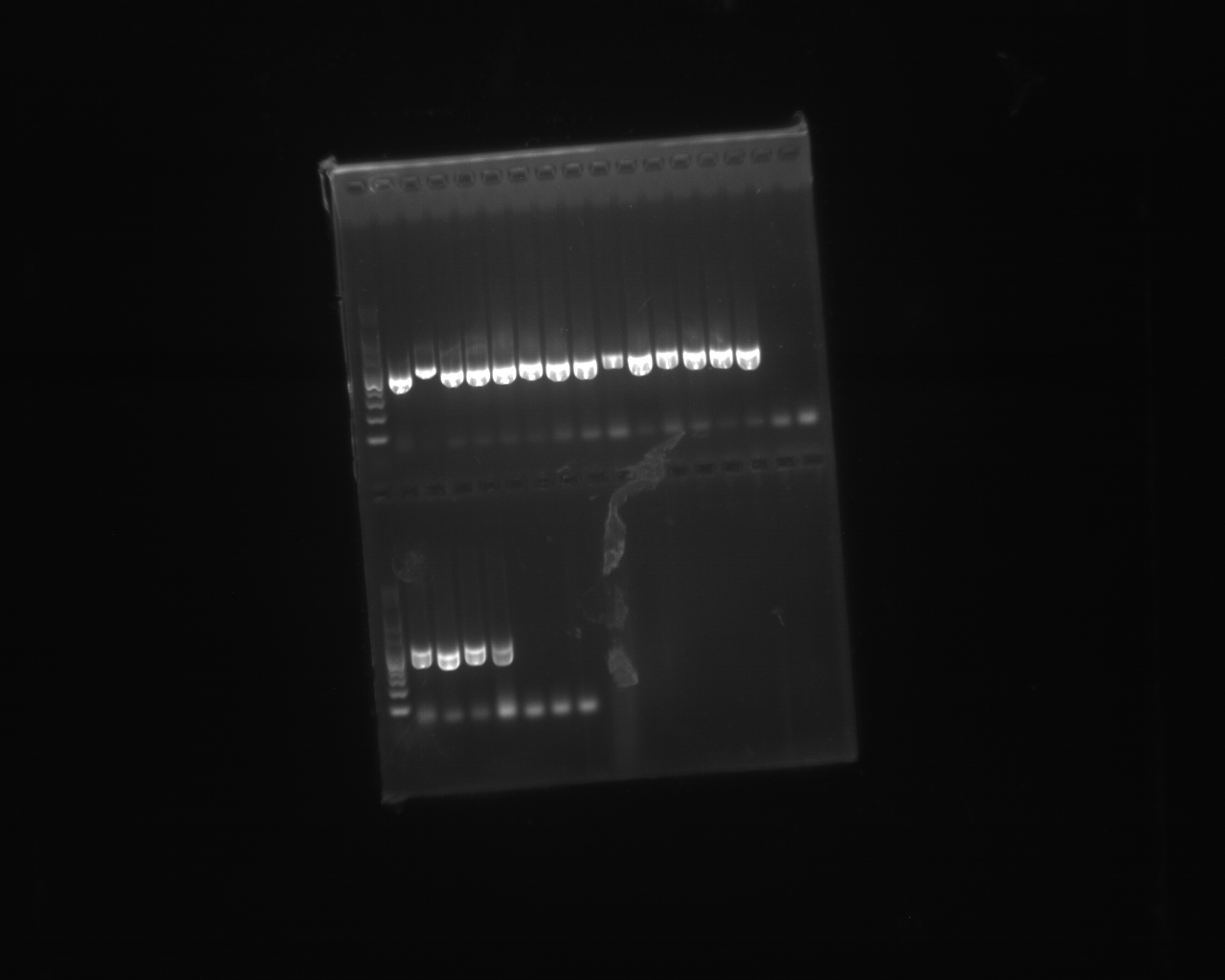

Supplement: Figure 3—source data 2. [file elife-89532-fig3-data2.zip › Figure 3-Source Data 2/E1/Row image/actin confirm .Tif]

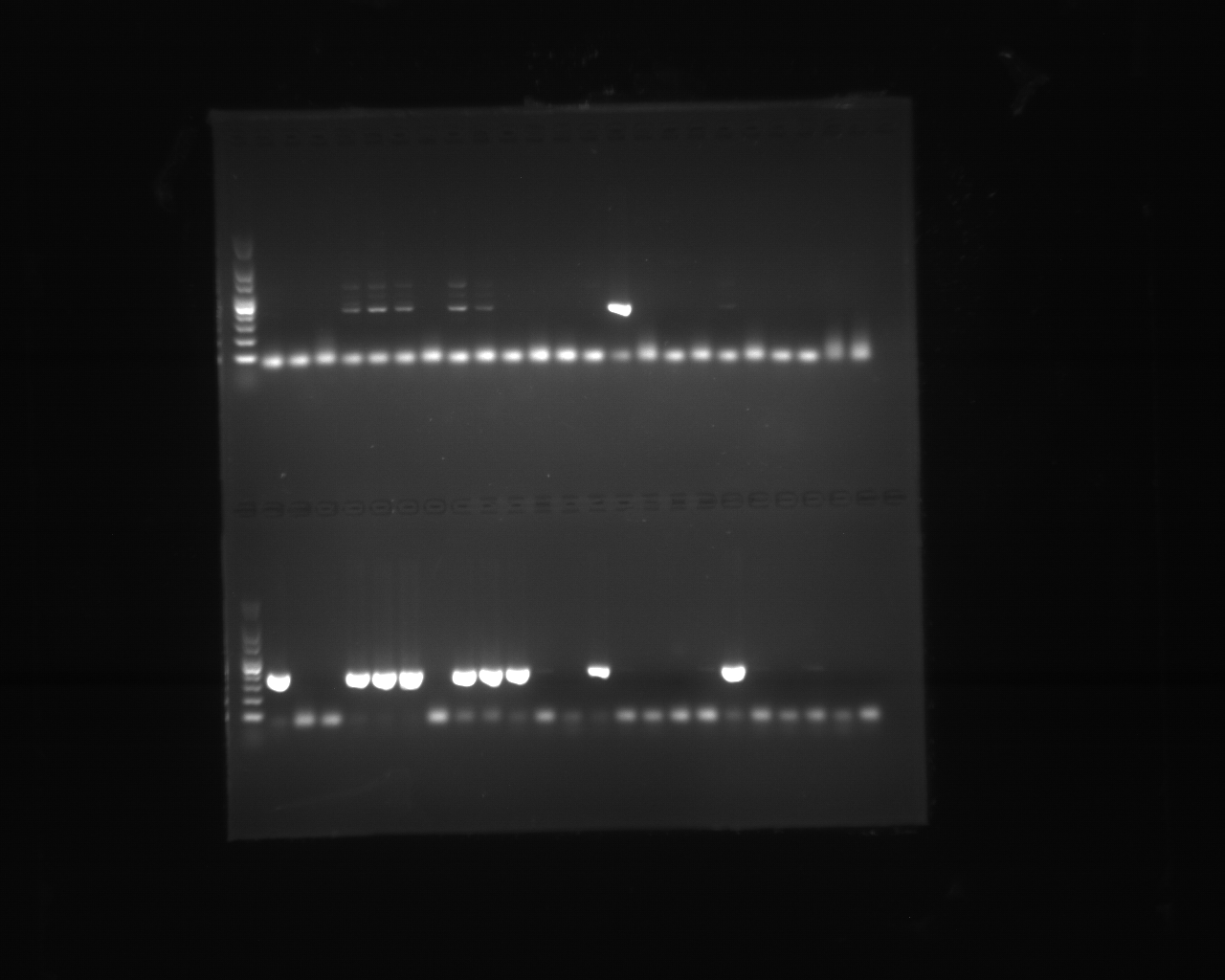

Supplement: Figure 3—source data 2. [file elife-89532-fig3-data2.zip › Figure 3-Source Data 2/E1/Row image/ga1 g2 .Tif]

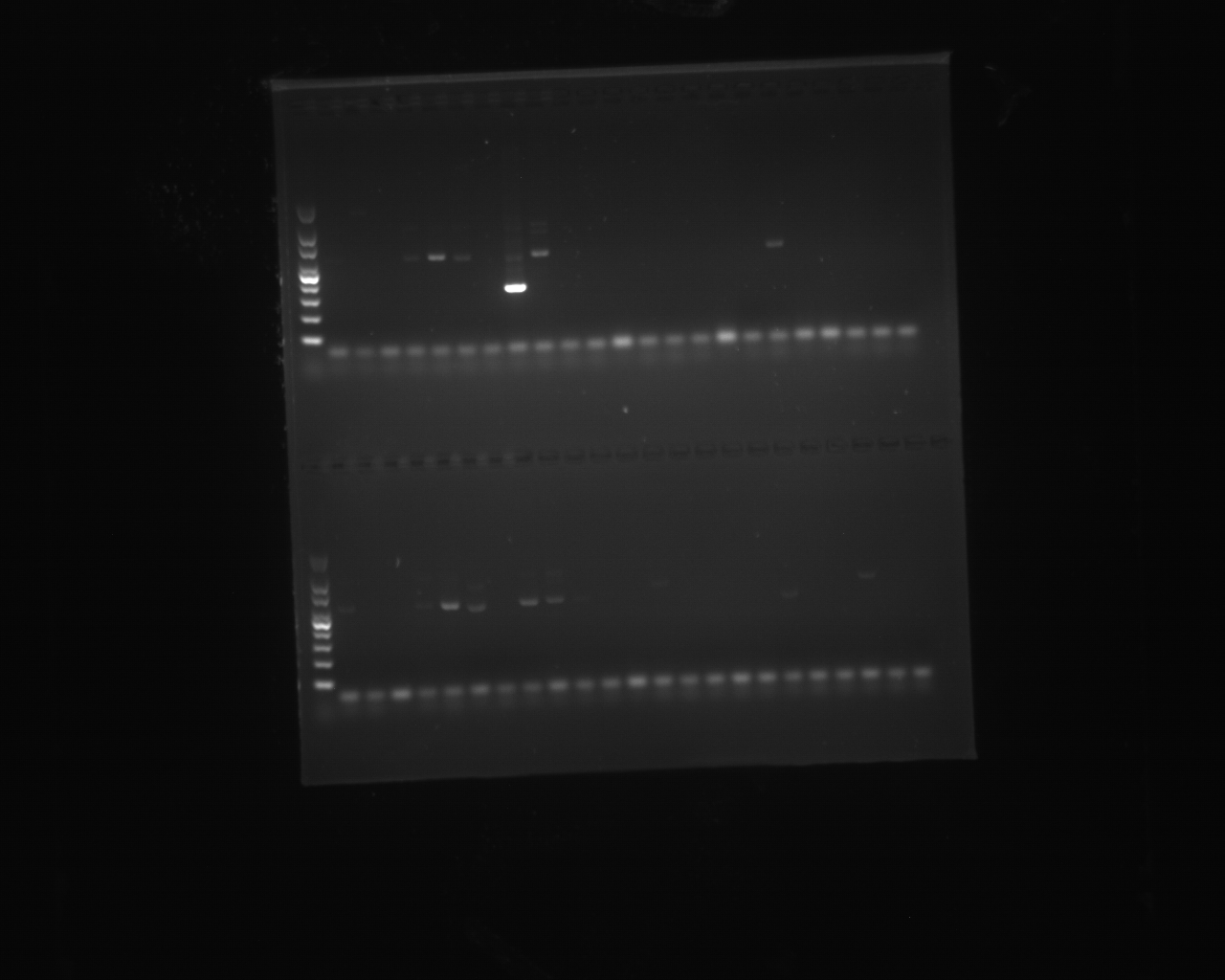

Supplement: Figure 3—source data 2. [file elife-89532-fig3-data2.zip › Figure 3-Source Data 2/E1/Row image/ga11,ga12 .Tif]

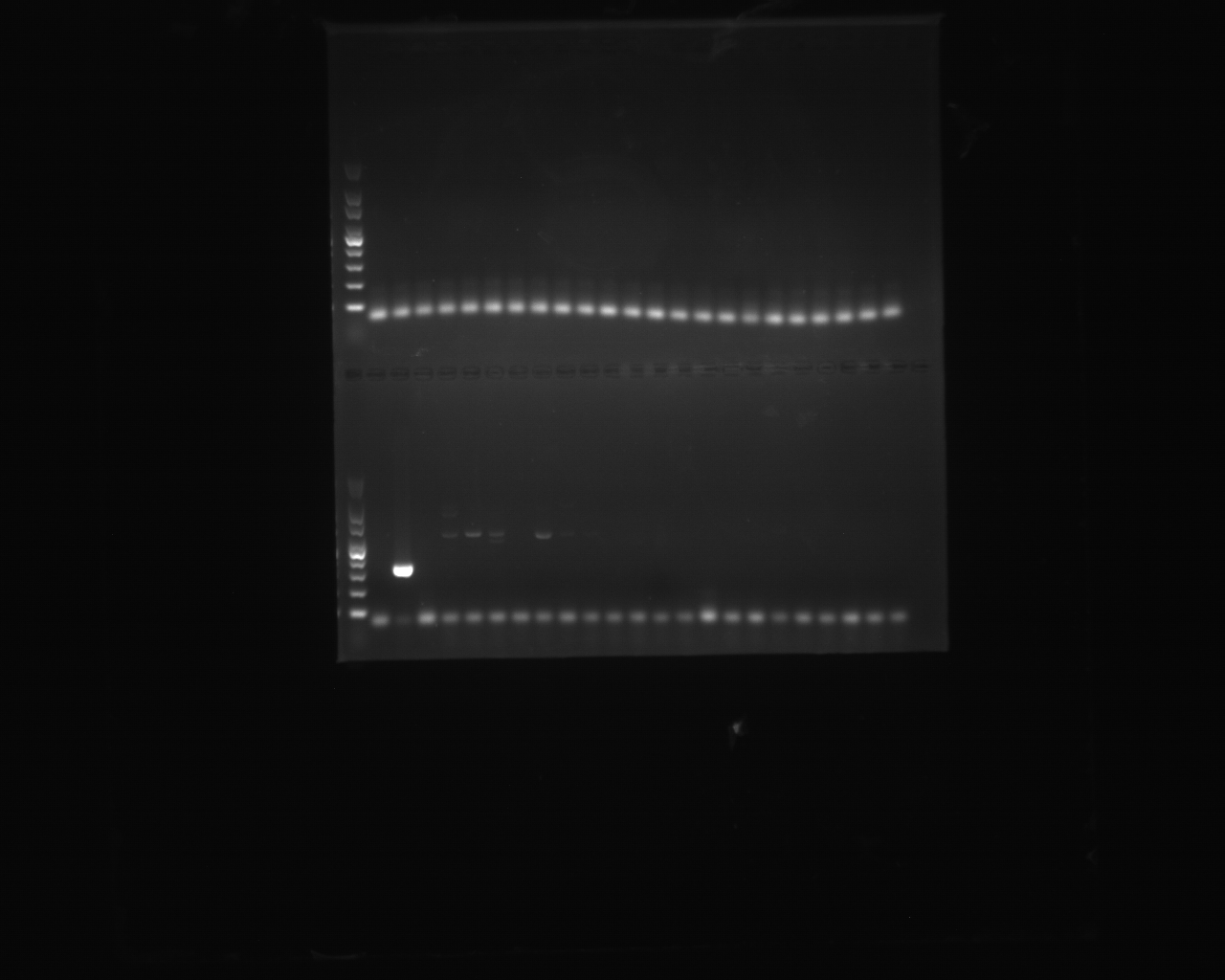

Supplement: Figure 3—source data 2. [file elife-89532-fig3-data2.zip › Figure 3-Source Data 2/E1/Row image/ga3,ga4 .Tif]

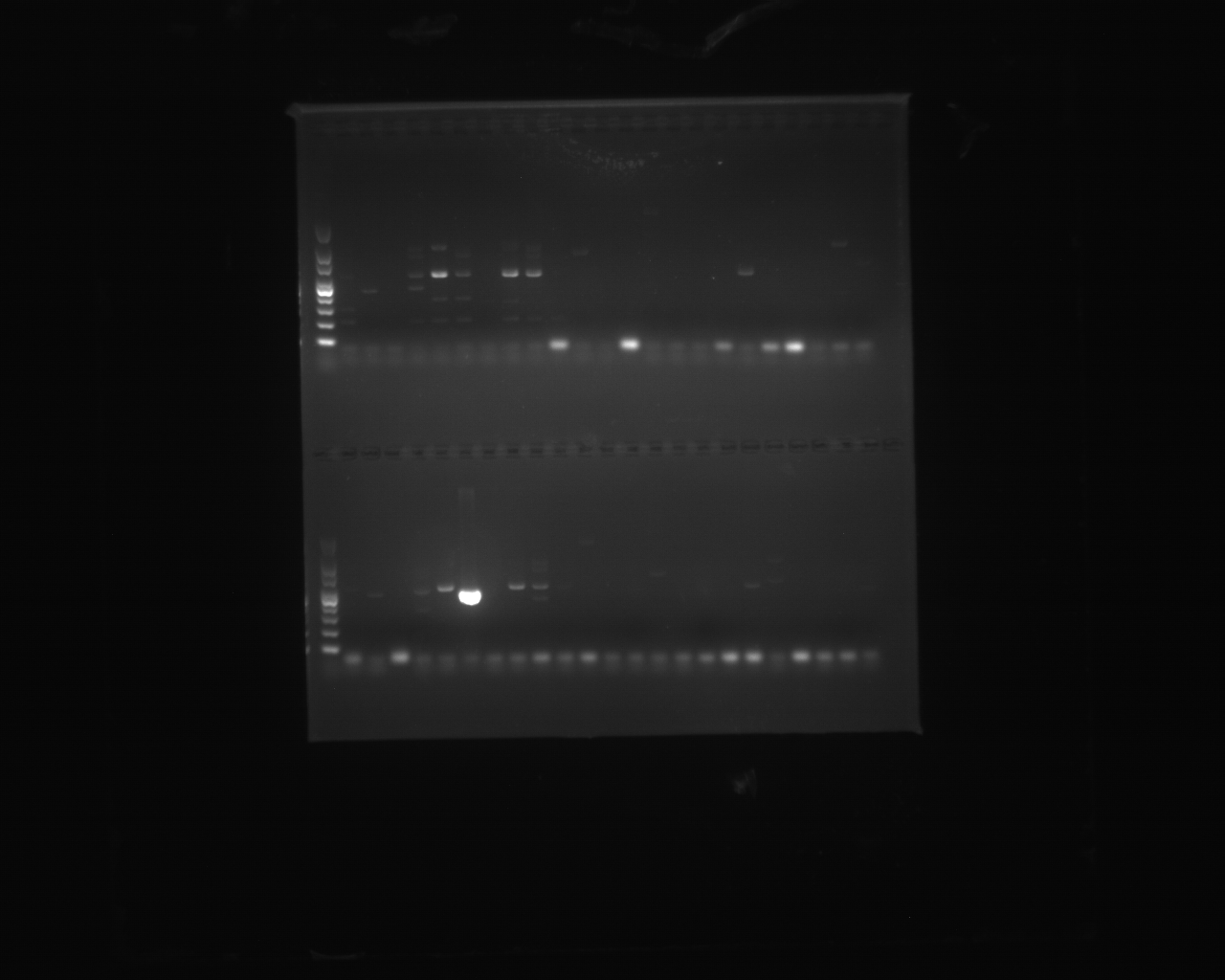

Supplement: Figure 3—source data 2. [file elife-89532-fig3-data2.zip › Figure 3-Source Data 2/E1/Row image/ga5,ga6 .Tif]

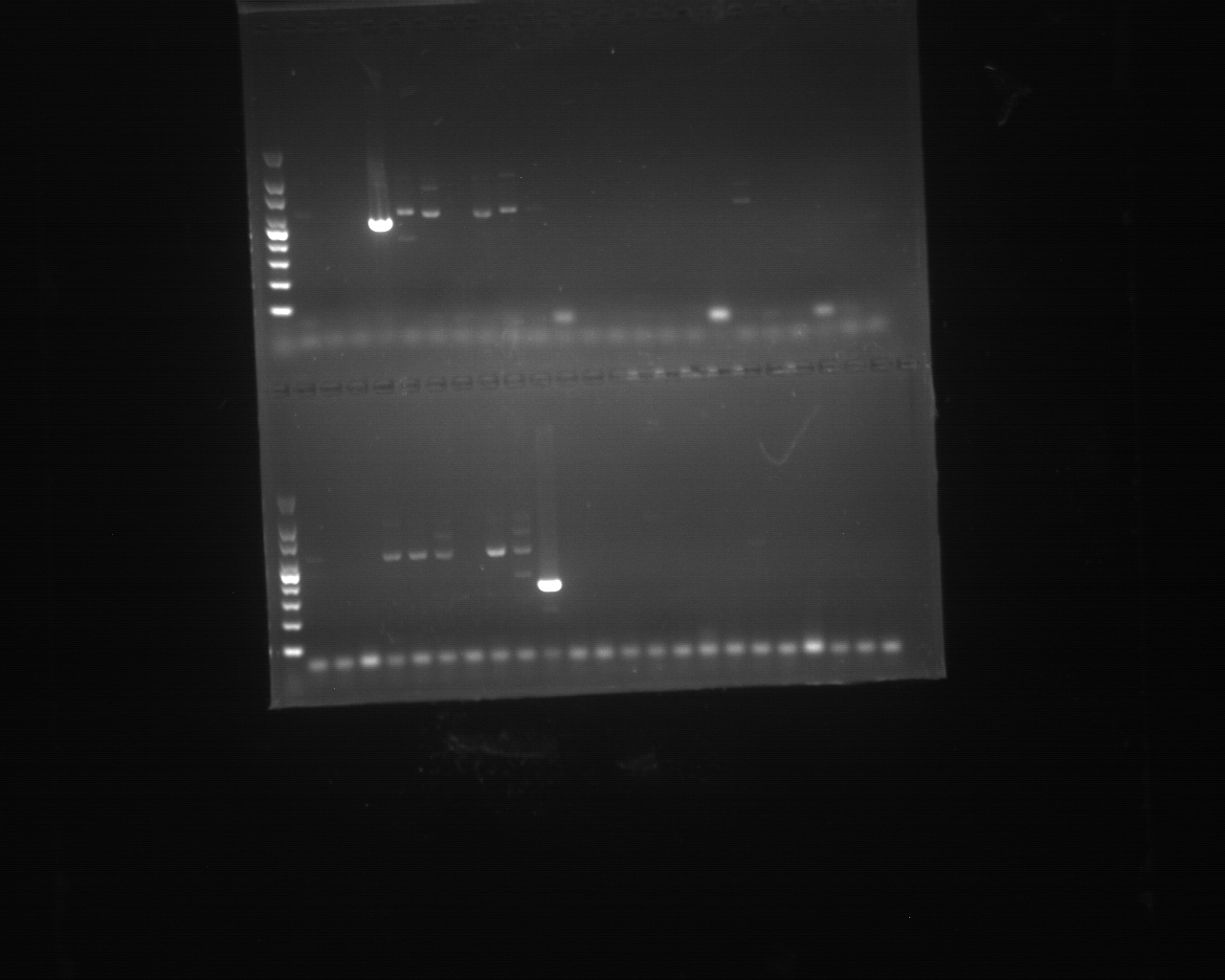

Supplement: Figure 3—source data 2. [file elife-89532-fig3-data2.zip › Figure 3-Source Data 2/E1/Row image/ga7,ga9 .Tif]

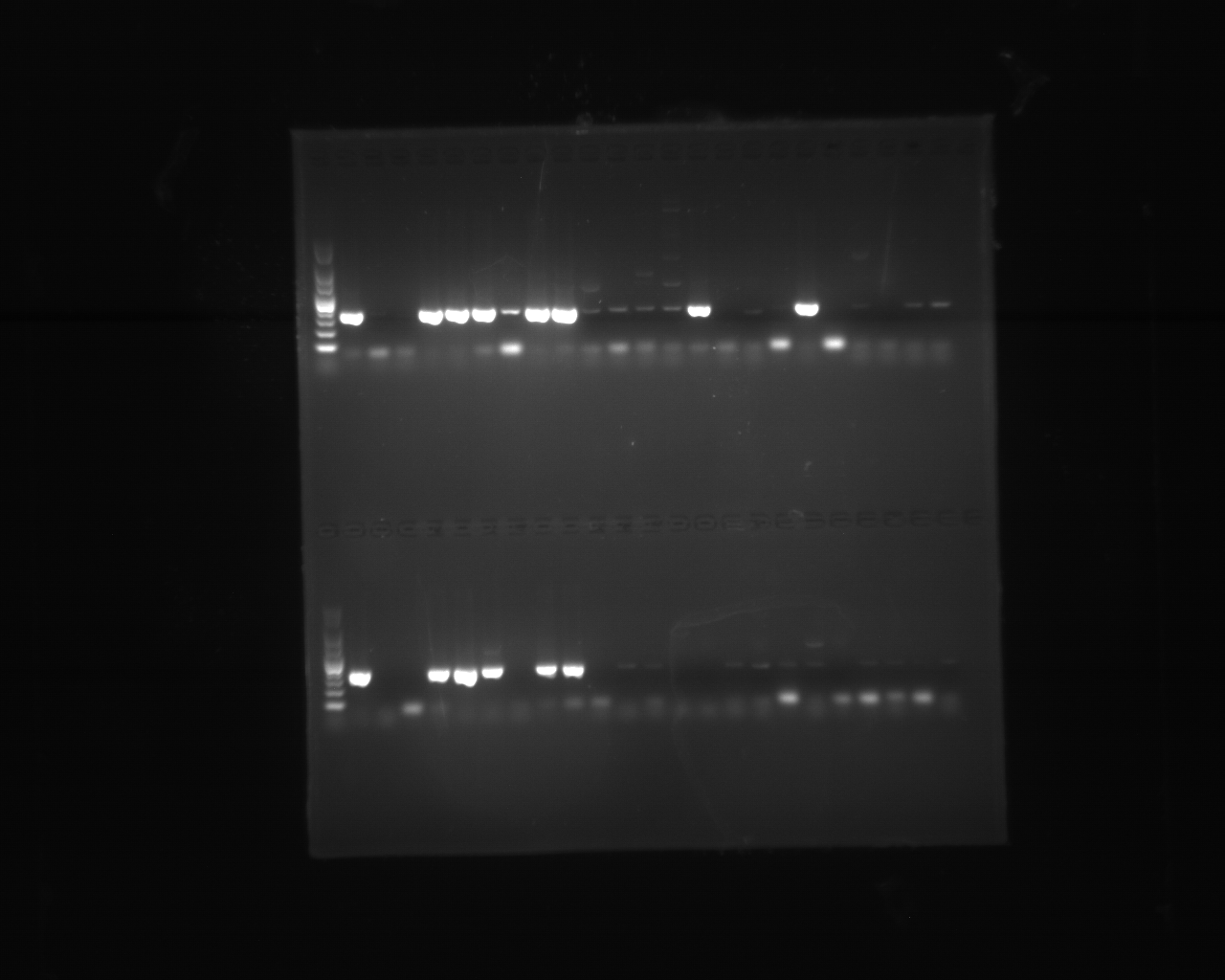

Supplement: Figure 3—source data 2. [file elife-89532-fig3-data2.zip › Figure 3-Source Data 2/E1/Row image/ga8 g10 .Tif]

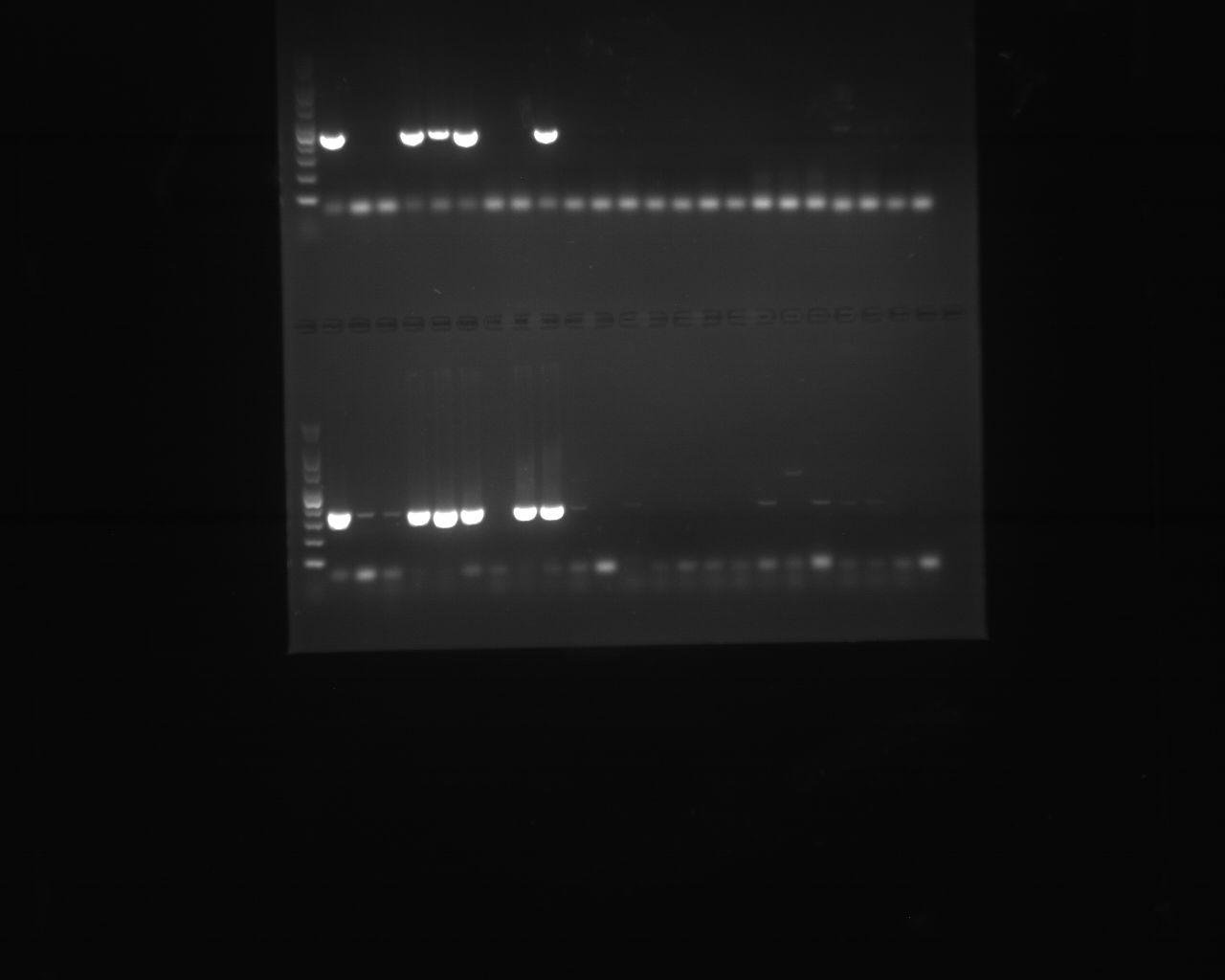

Supplement: Figure 3—source data 2. [file elife-89532-fig3-data2.zip › Figure 3-Source Data 2/E1/Row image/gb1 gb2 .Tif]

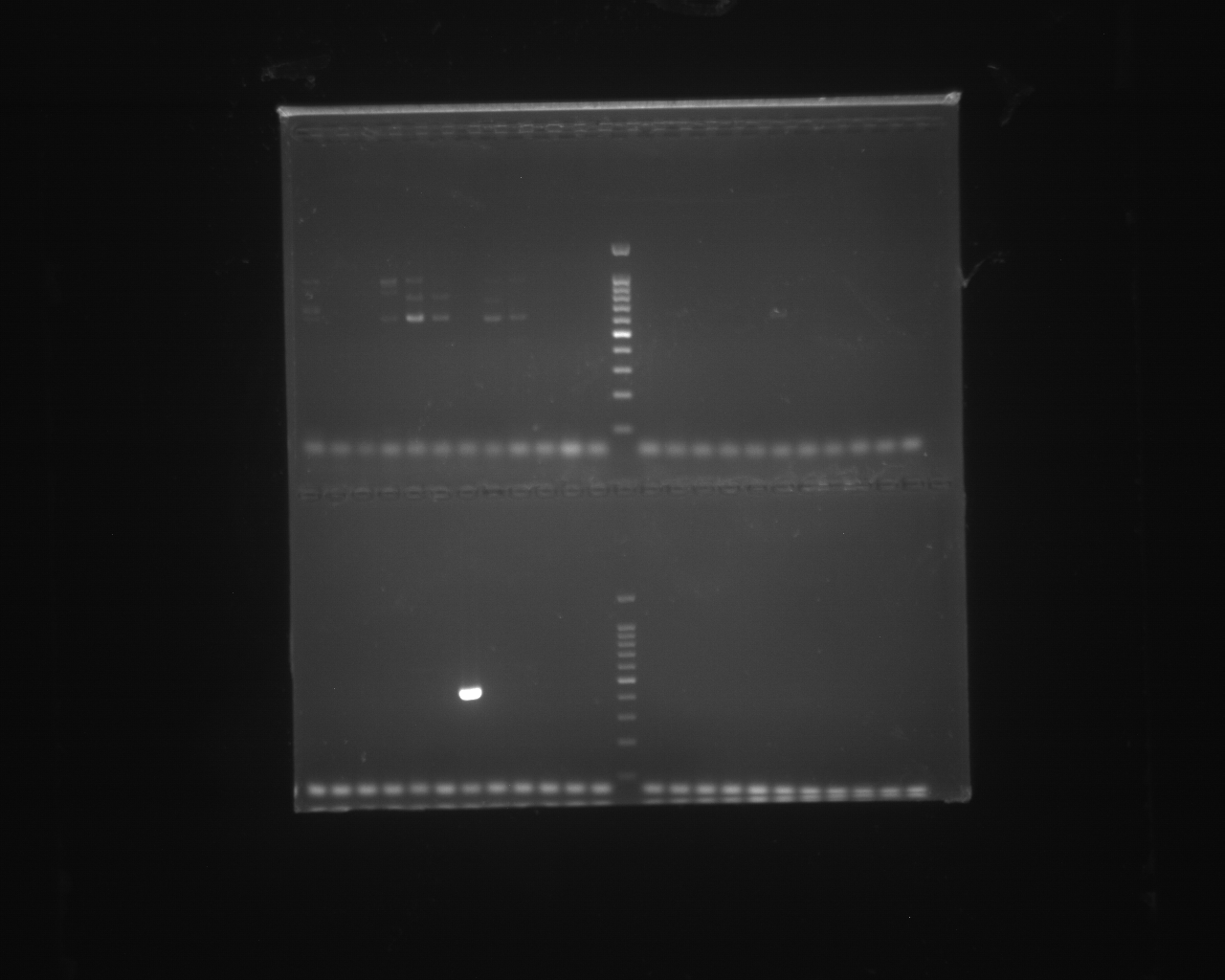

Supplement: Figure 3—source data 2. [file elife-89532-fig3-data2.zip › Figure 3-Source Data 2/E1/Row image/gb4,gb5 .Tif]

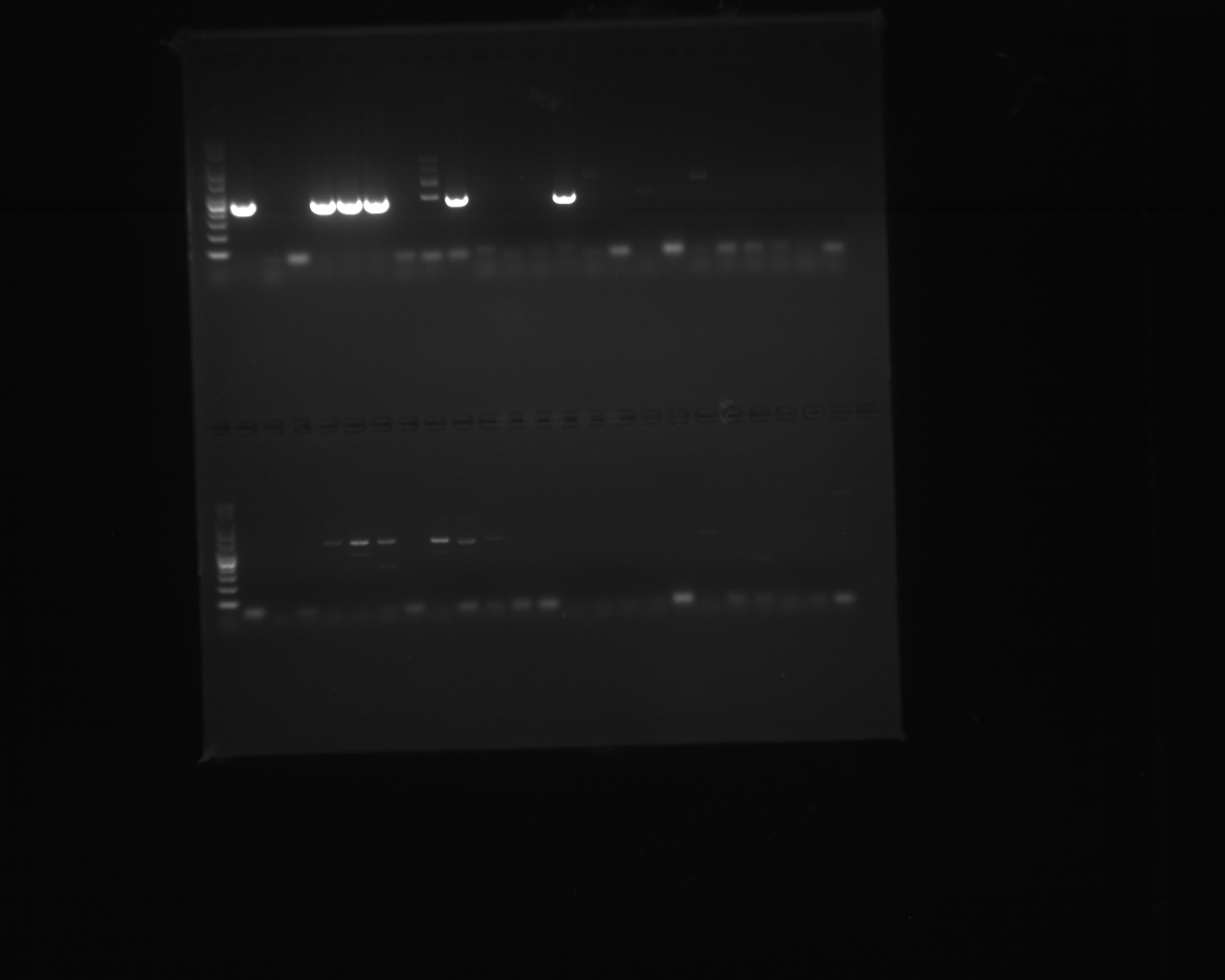

Supplement: Figure 3—source data 2. [file elife-89532-fig3-data2.zip › Figure 3-Source Data 2/E1/Row image/gb6 gb7.Tif]

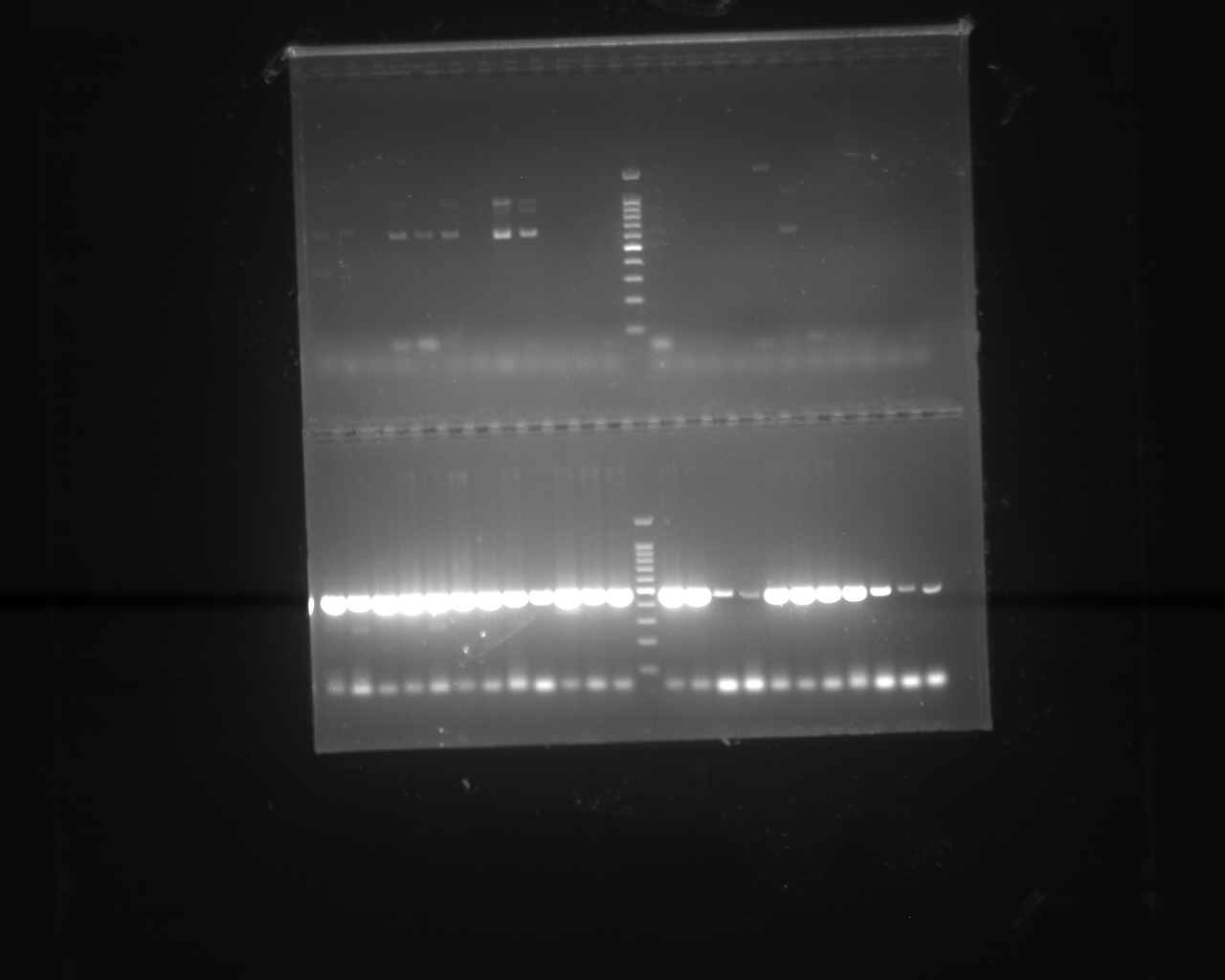

Supplement: Figure 3—source data 2. [file elife-89532-fig3-data2.zip › Figure 3-Source Data 2/E1/Row image/gb8,actb (2).Tif]

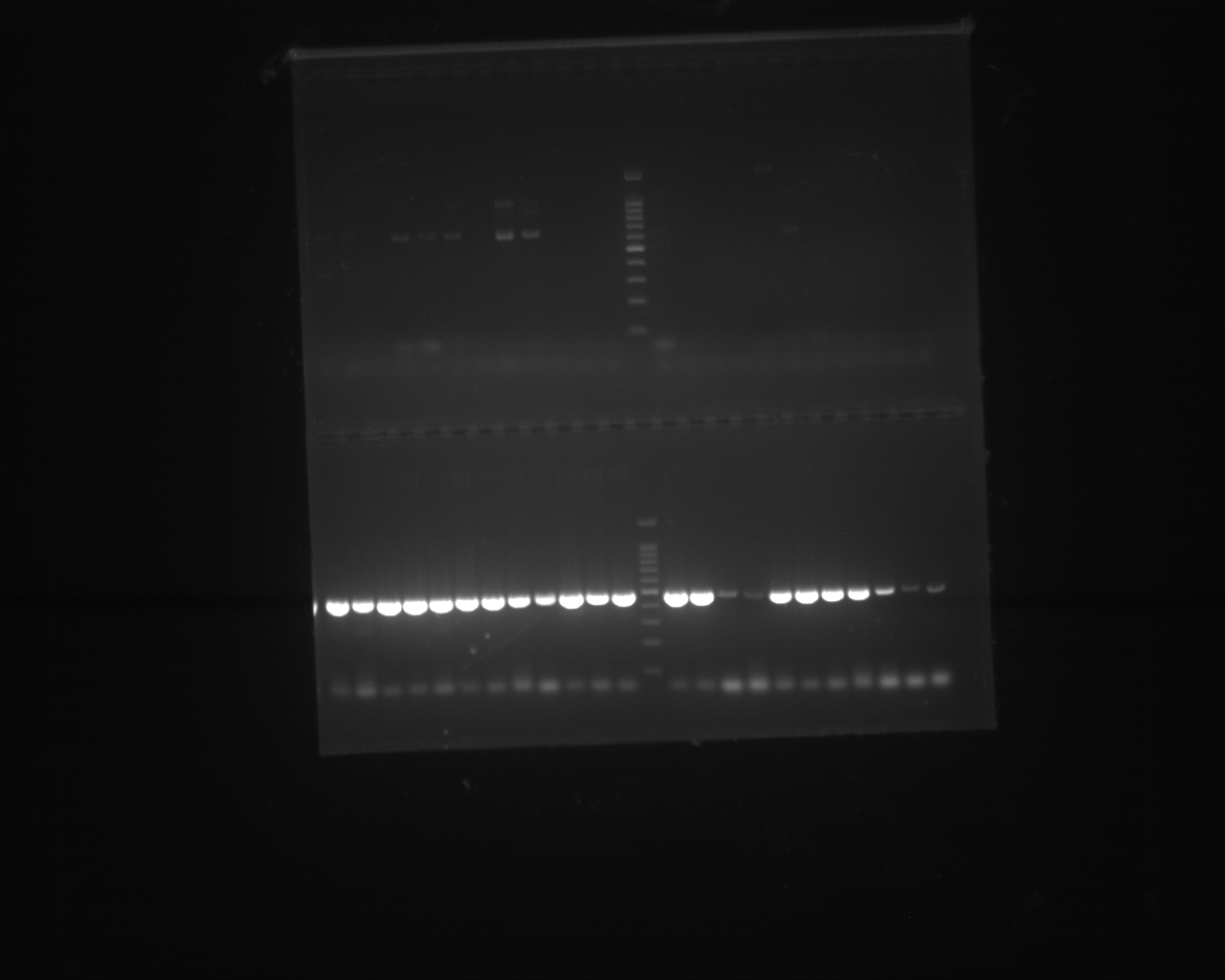

Supplement: Figure 3—source data 2. [file elife-89532-fig3-data2.zip › Figure 3-Source Data 2/E1/Row image/gb8,actb .Tif]

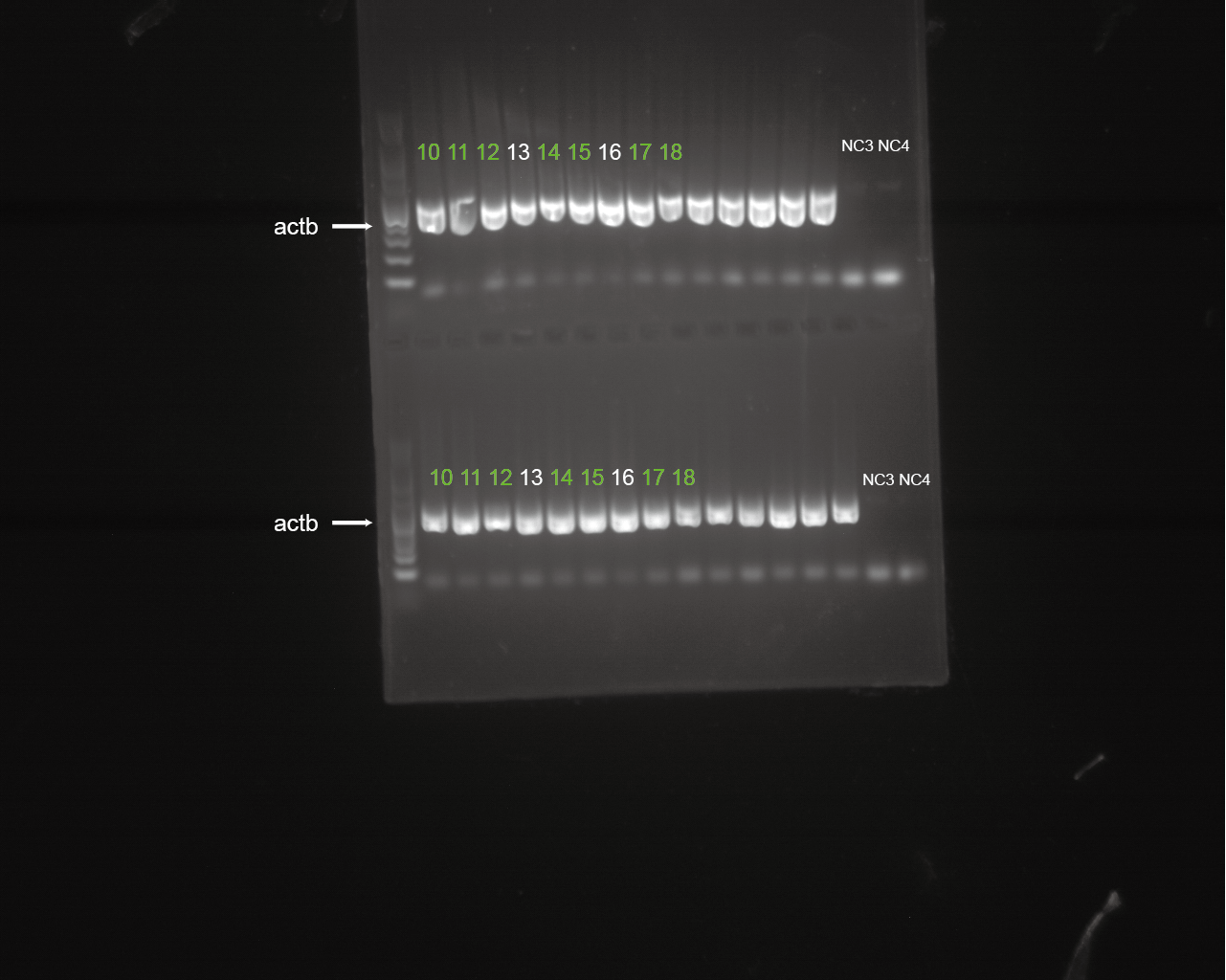

Supplement: Figure 3—source data 2. [file elife-89532-fig3-data2.zip › Figure 3-Source Data 2/E2/labelled image/actin confirm.tif]

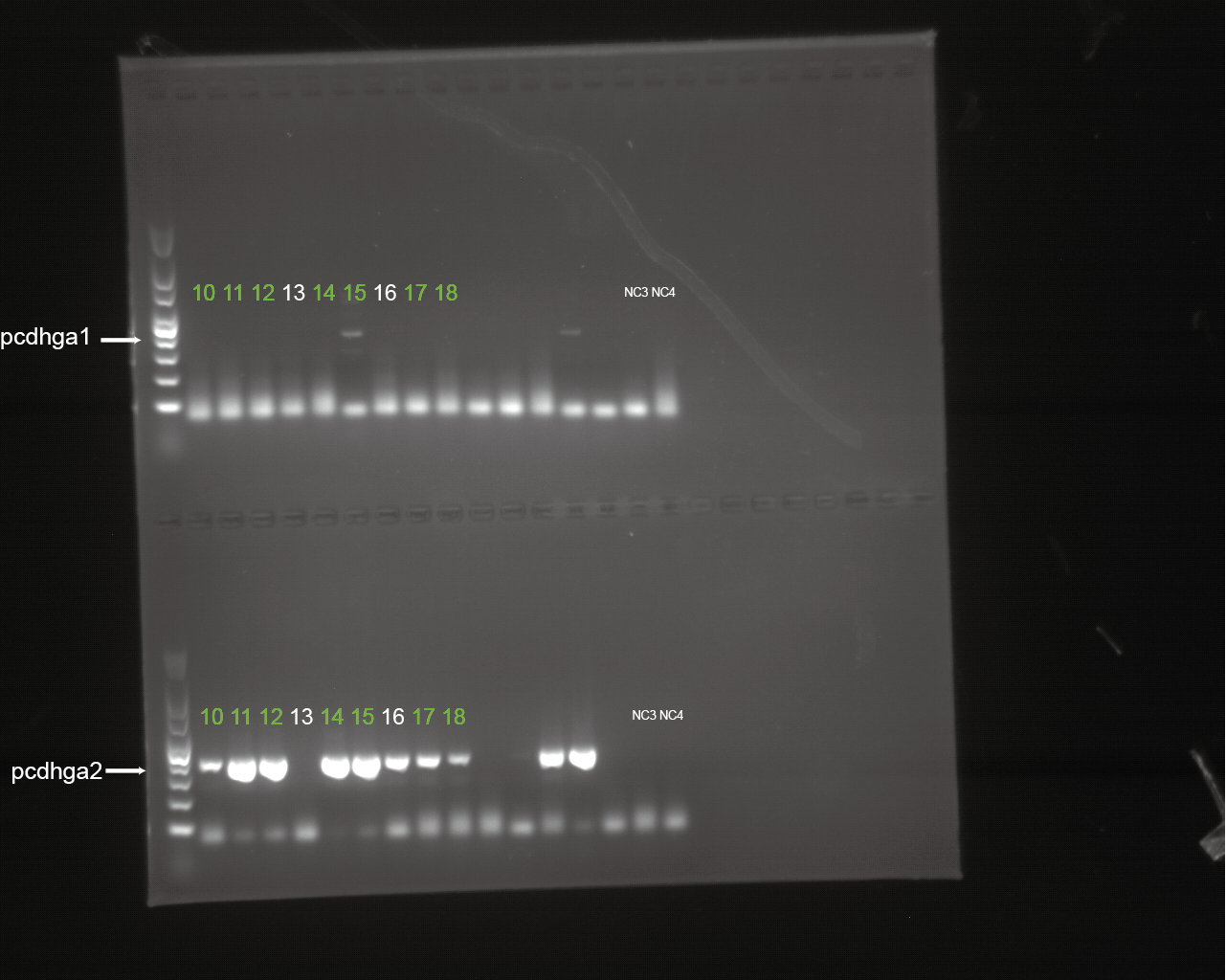

Supplement: Figure 3—source data 2. [file elife-89532-fig3-data2.zip › Figure 3-Source Data 2/E2/labelled image/ga1 ga2 .tif]

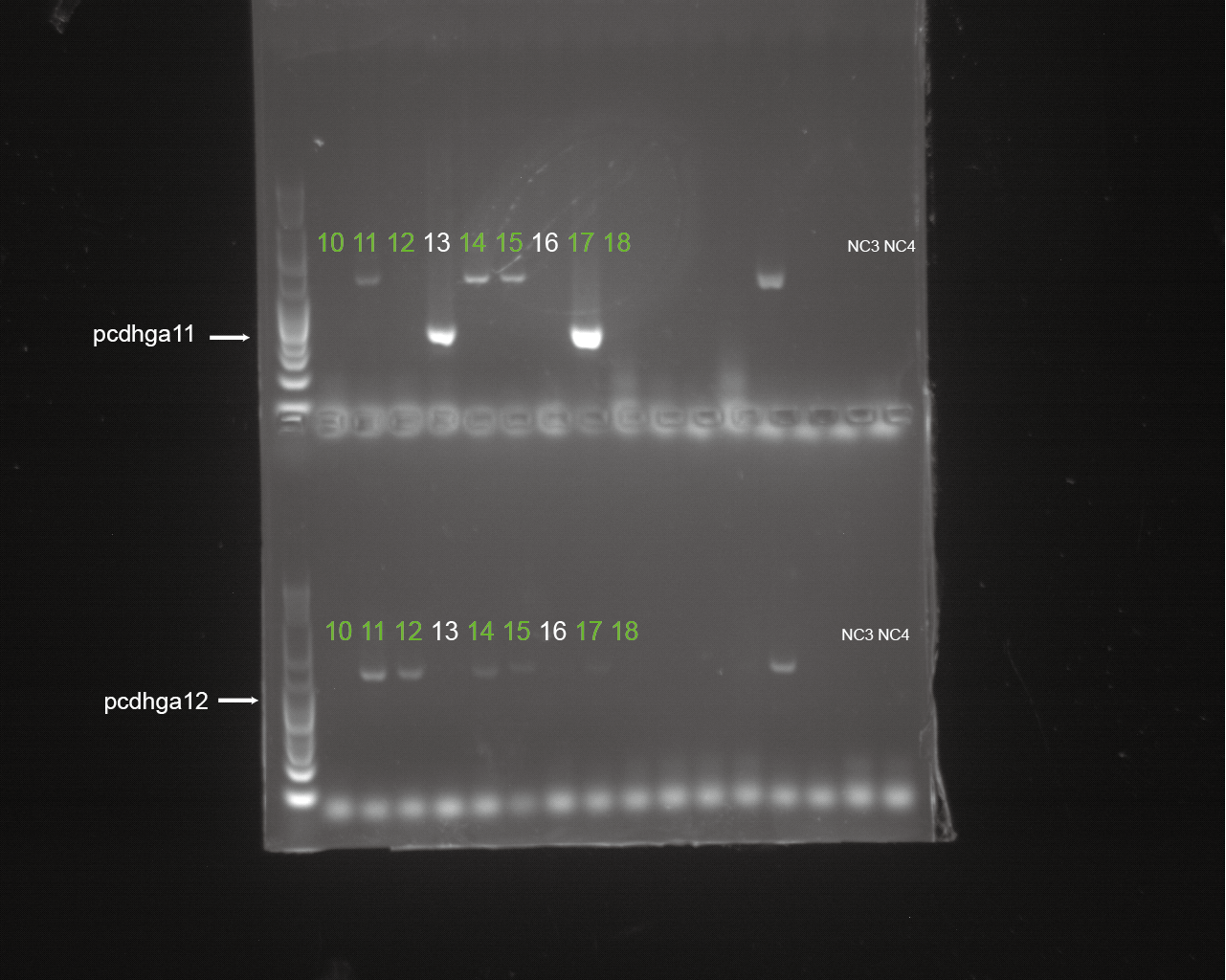

Supplement: Figure 3—source data 2. [file elife-89532-fig3-data2.zip › Figure 3-Source Data 2/E2/labelled image/ga11 ga12 .tif]

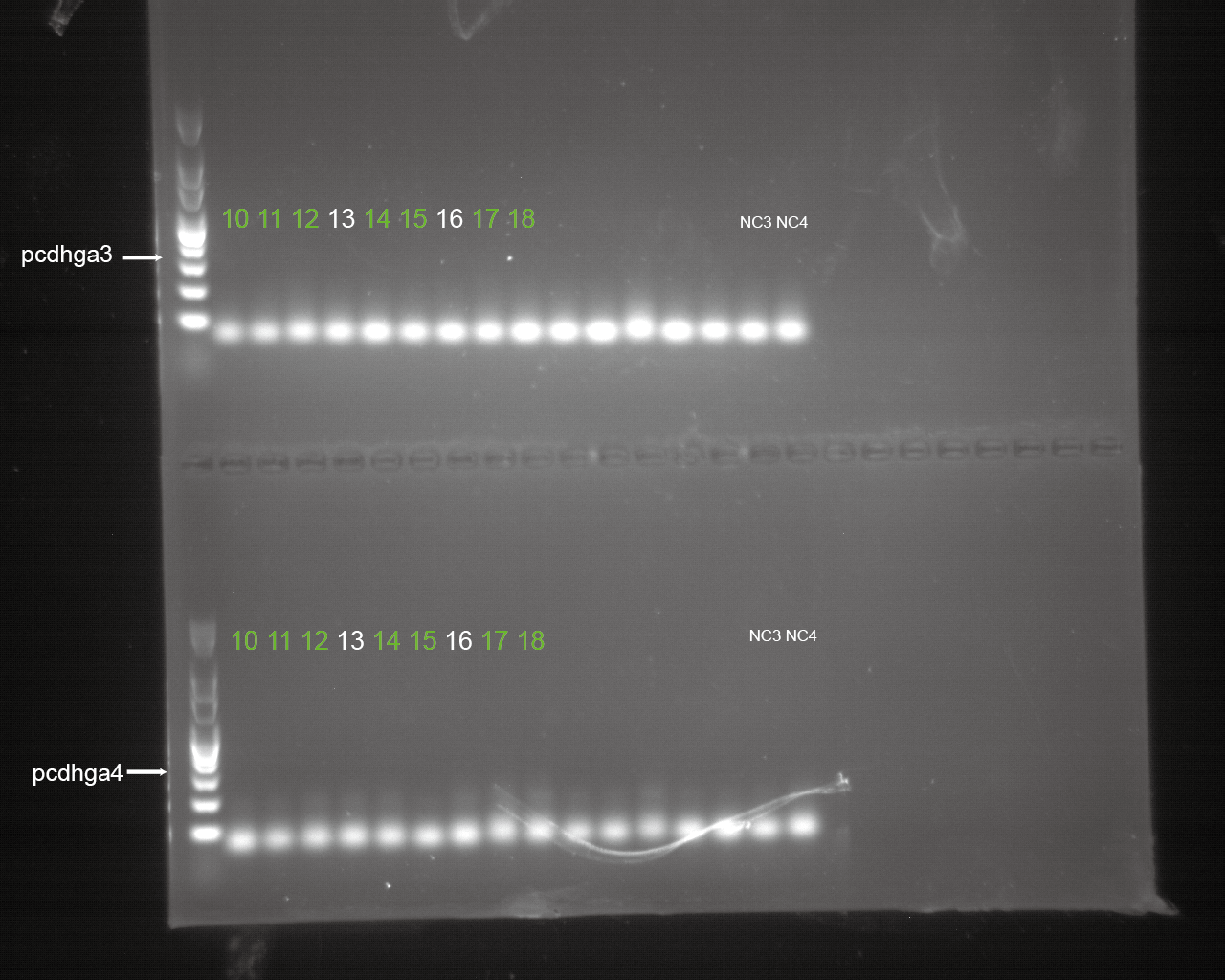

Supplement: Figure 3—source data 2. [file elife-89532-fig3-data2.zip › Figure 3-Source Data 2/E2/labelled image/ga3 ga4 .tif]

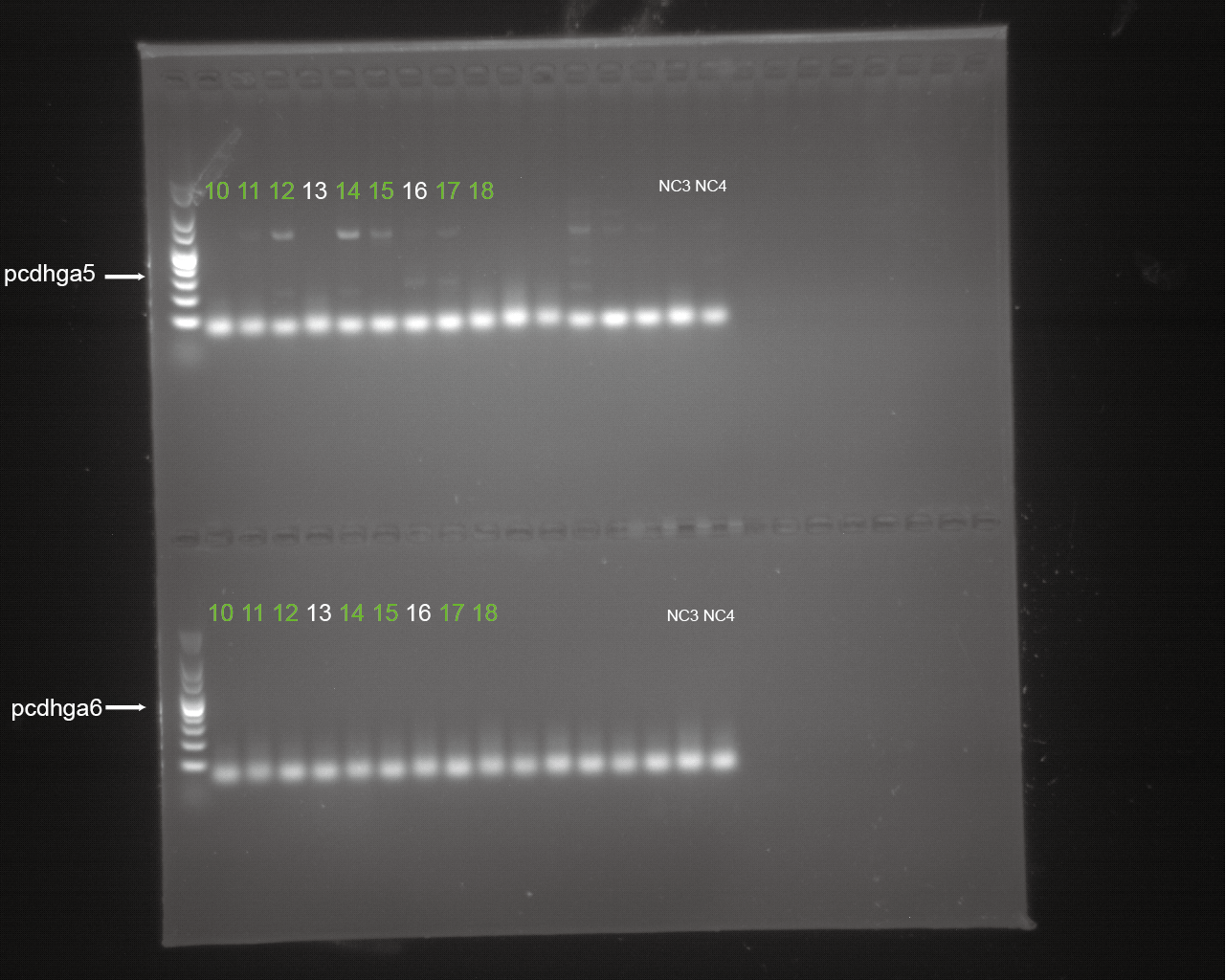

Supplement: Figure 3—source data 2. [file elife-89532-fig3-data2.zip › Figure 3-Source Data 2/E2/labelled image/ga5 ga6 .tif]

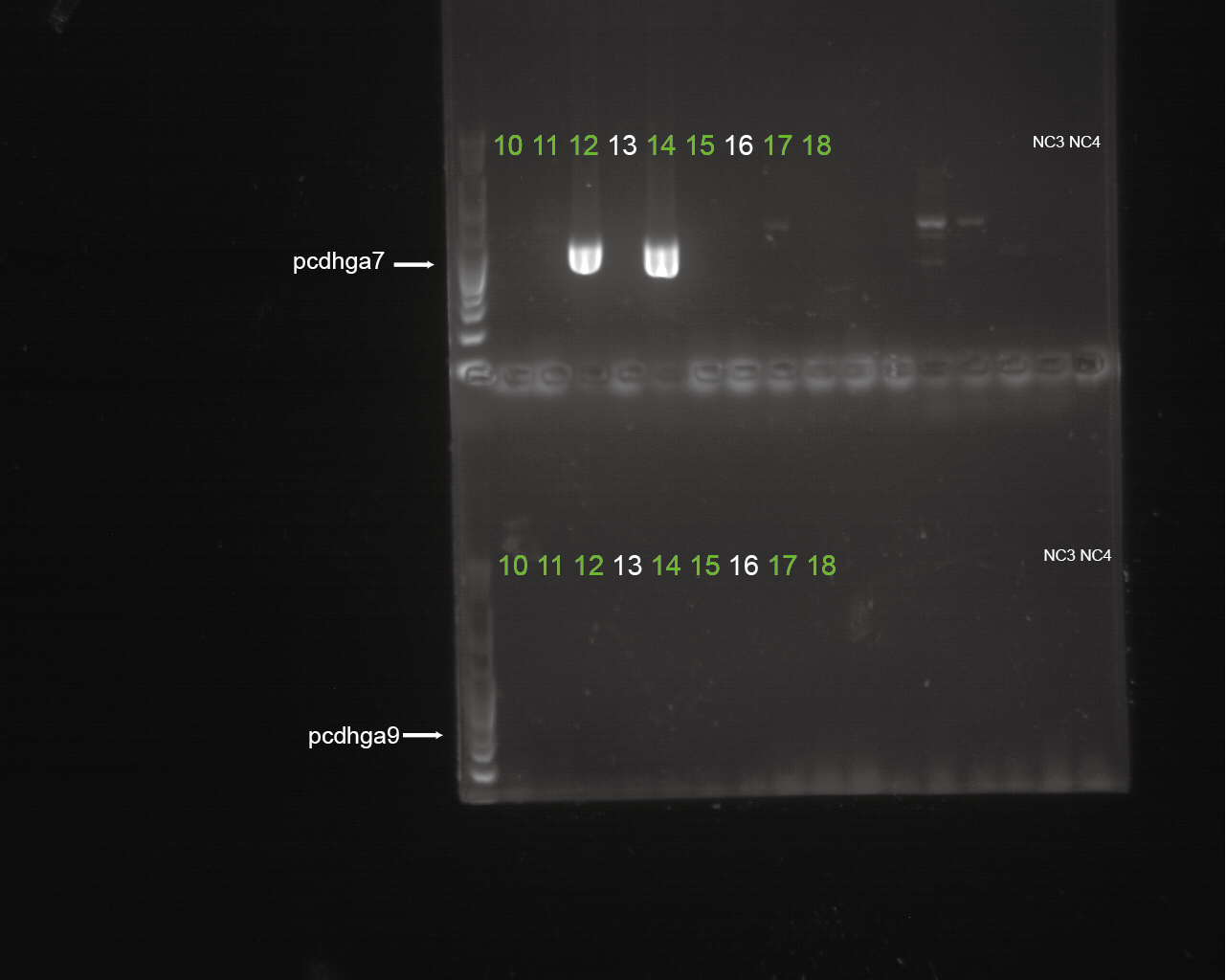

Supplement: Figure 3—source data 2. [file elife-89532-fig3-data2.zip › Figure 3-Source Data 2/E2/labelled image/ga7 ga9 .tif]

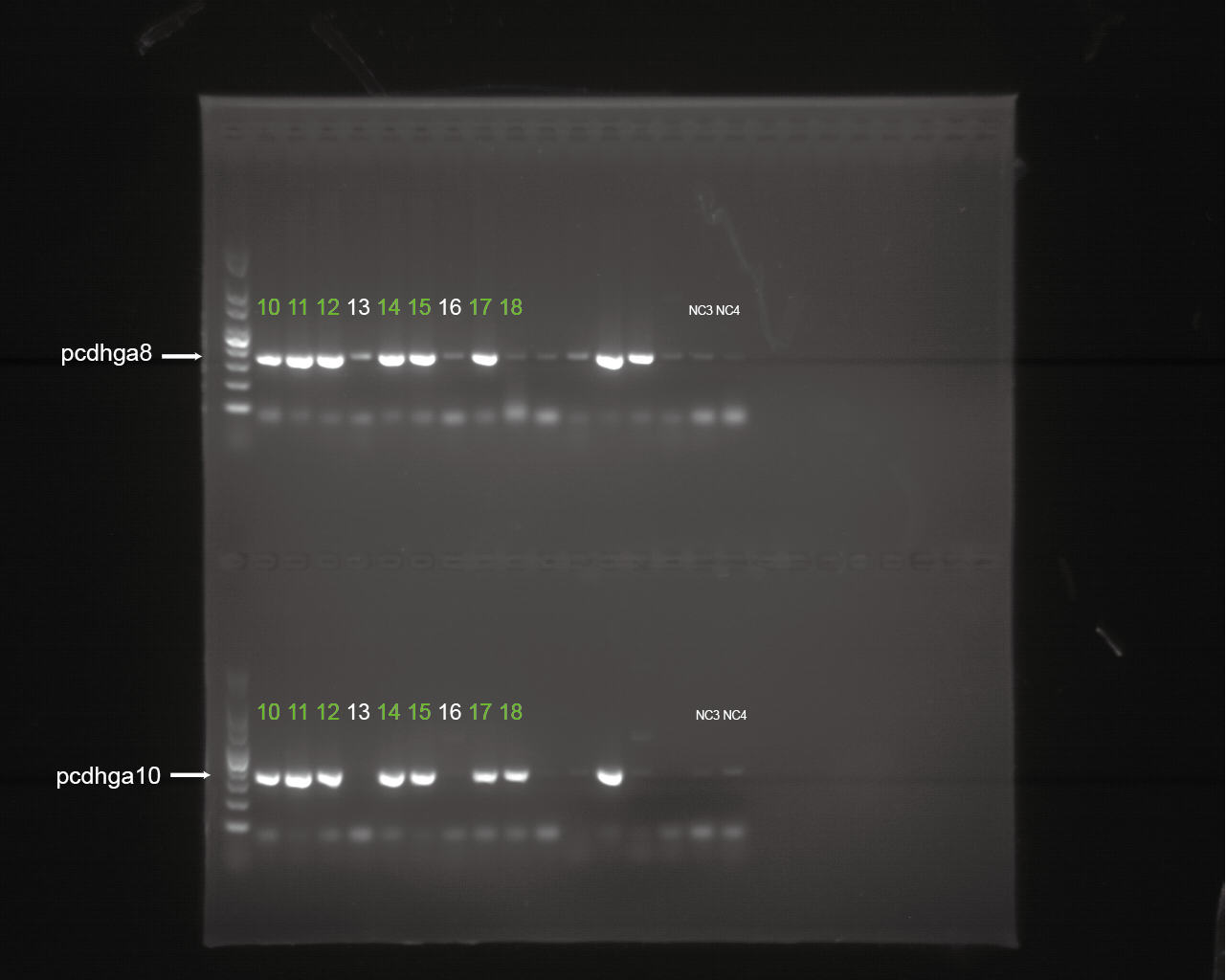

Supplement: Figure 3—source data 2. [file elife-89532-fig3-data2.zip › Figure 3-Source Data 2/E2/labelled image/ga8 ga10 .tif]

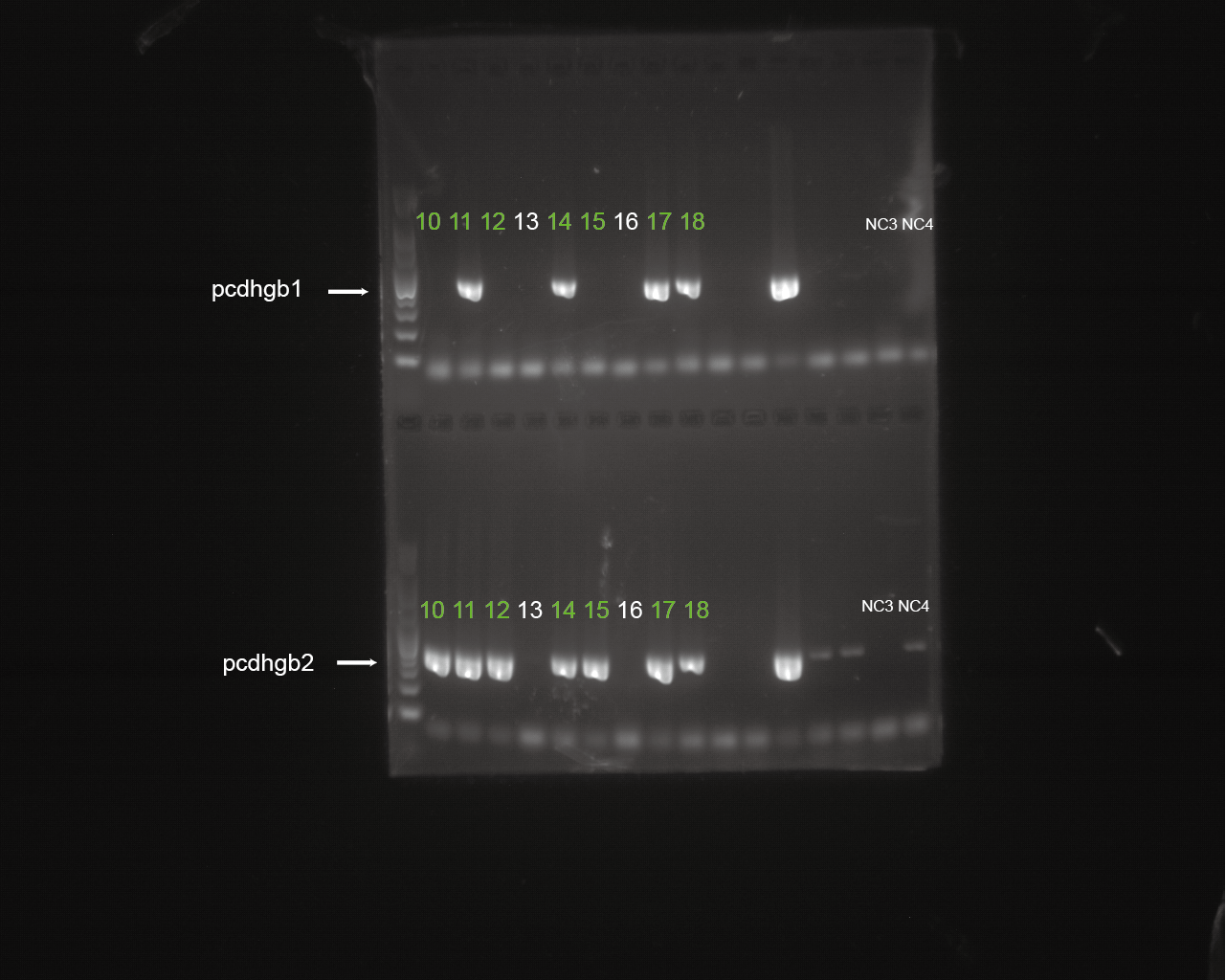

Supplement: Figure 3—source data 2. [file elife-89532-fig3-data2.zip › Figure 3-Source Data 2/E2/labelled image/gb1 b2 .tif]

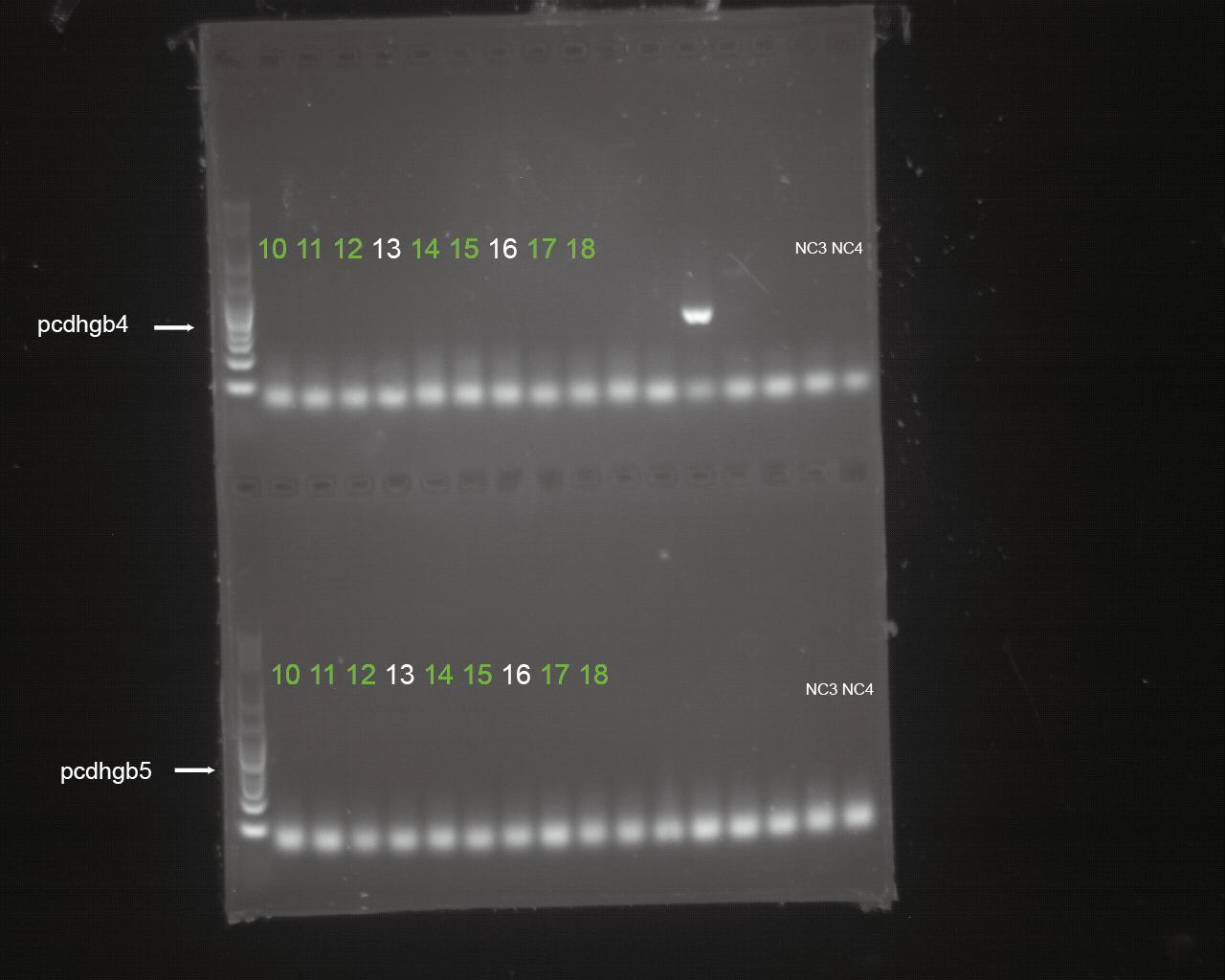

Supplement: Figure 3—source data 2. [file elife-89532-fig3-data2.zip › Figure 3-Source Data 2/E2/labelled image/gb4 gb5 .tif]

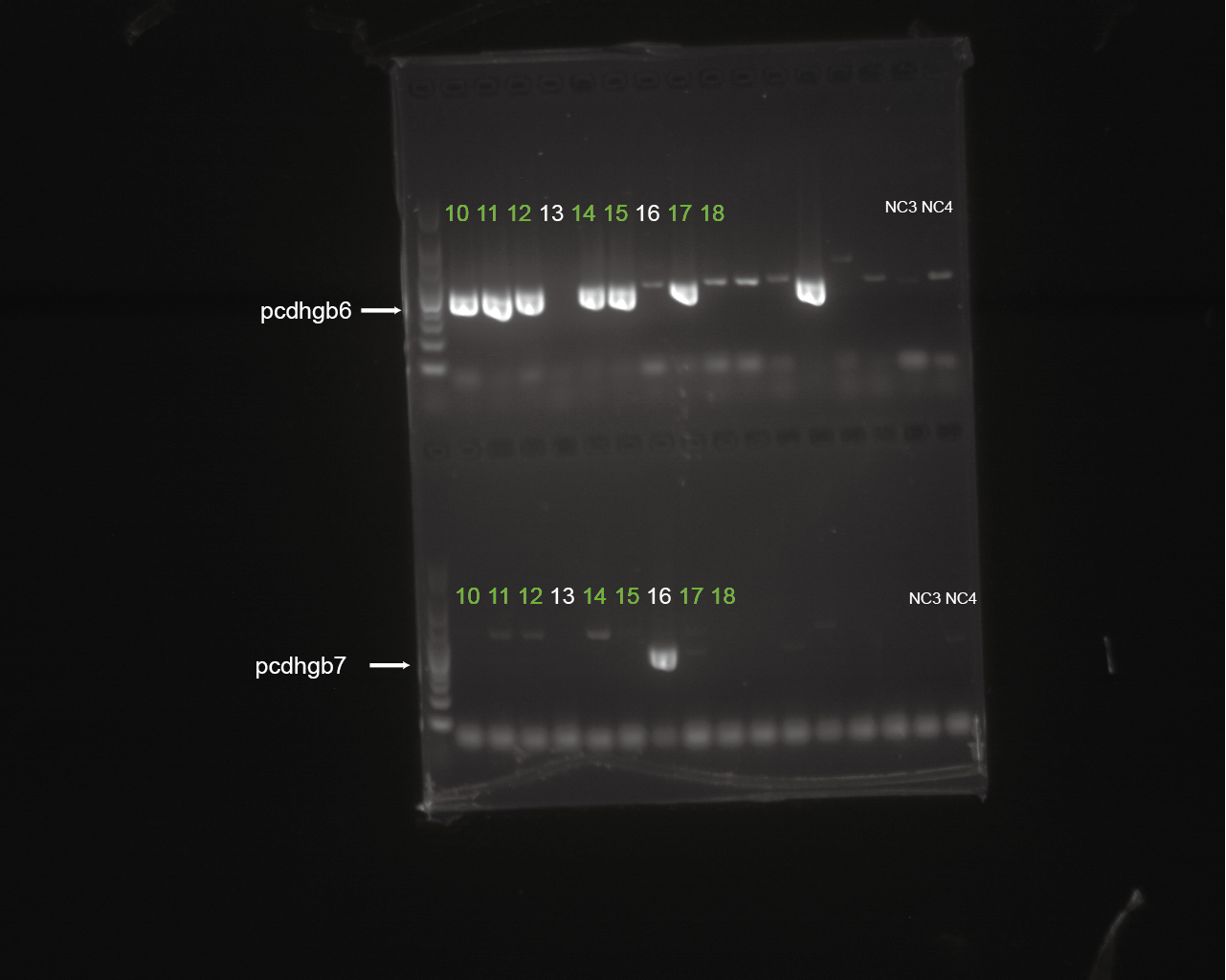

Supplement: Figure 3—source data 2. [file elife-89532-fig3-data2.zip › Figure 3-Source Data 2/E2/labelled image/gb6 b7 .tif]

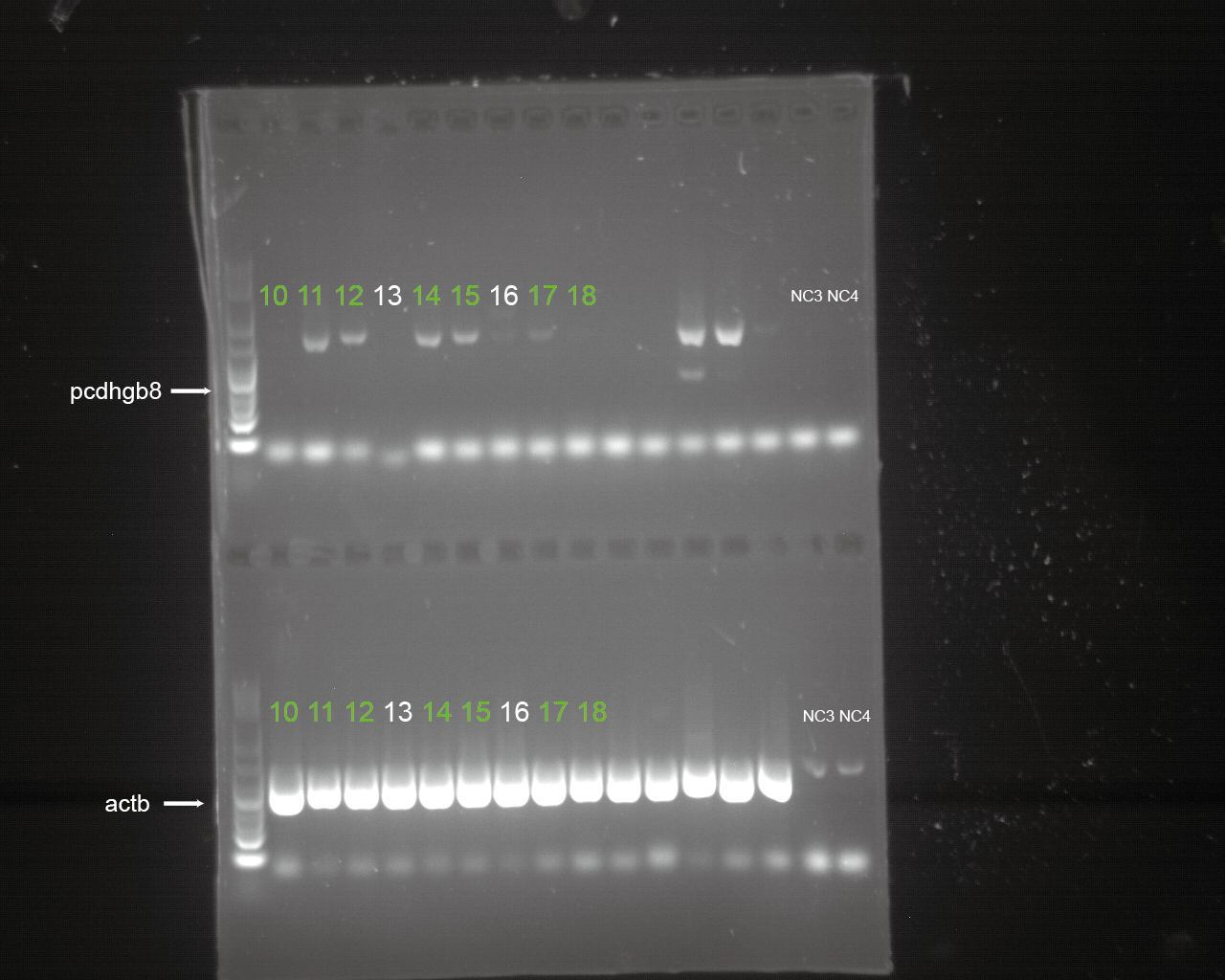

Supplement: Figure 3—source data 2. [file elife-89532-fig3-data2.zip › Figure 3-Source Data 2/E2/labelled image/gb8 actin (2).tif]

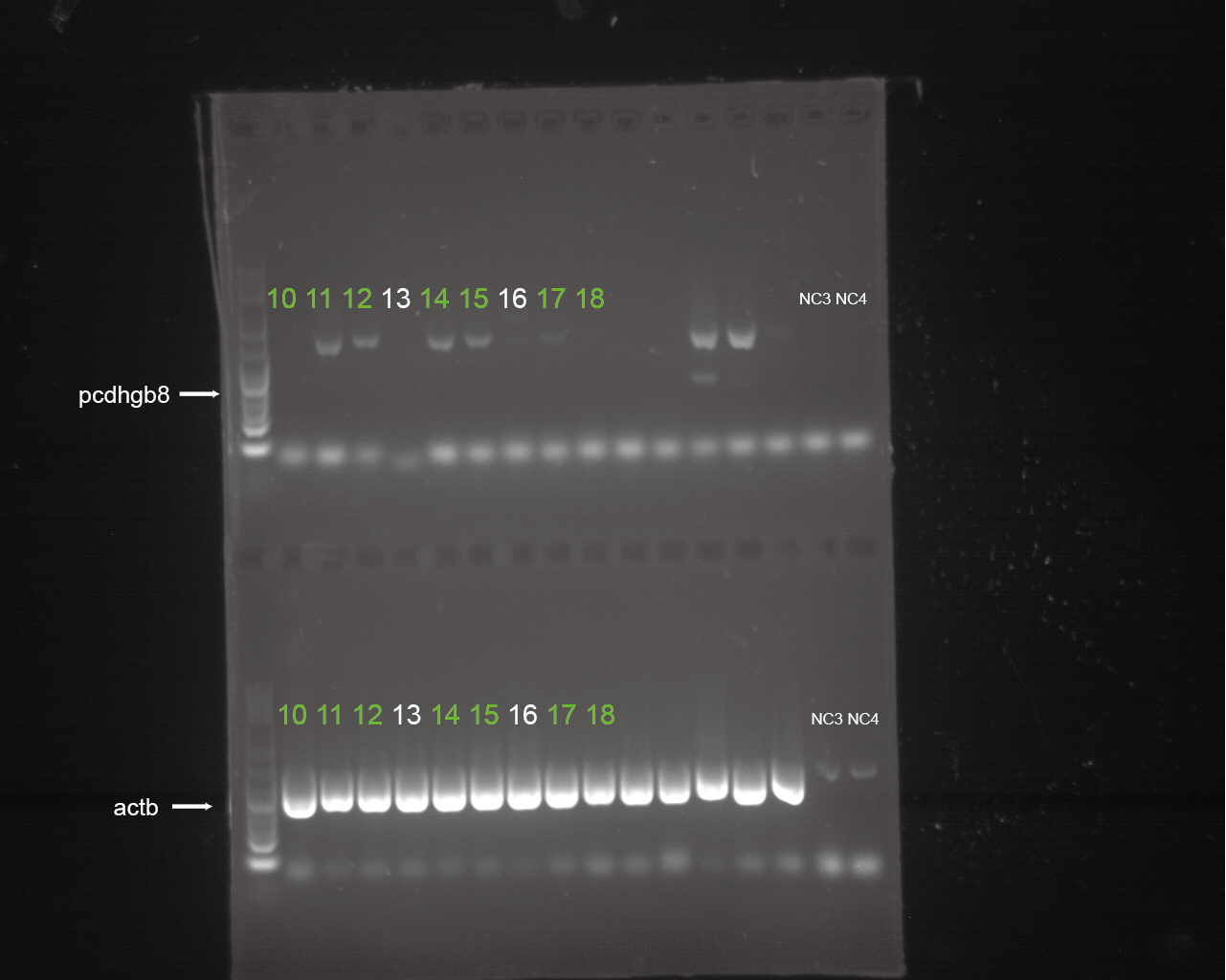

Supplement: Figure 3—source data 2. [file elife-89532-fig3-data2.zip › Figure 3-Source Data 2/E2/labelled image/gb8 actin .tif]

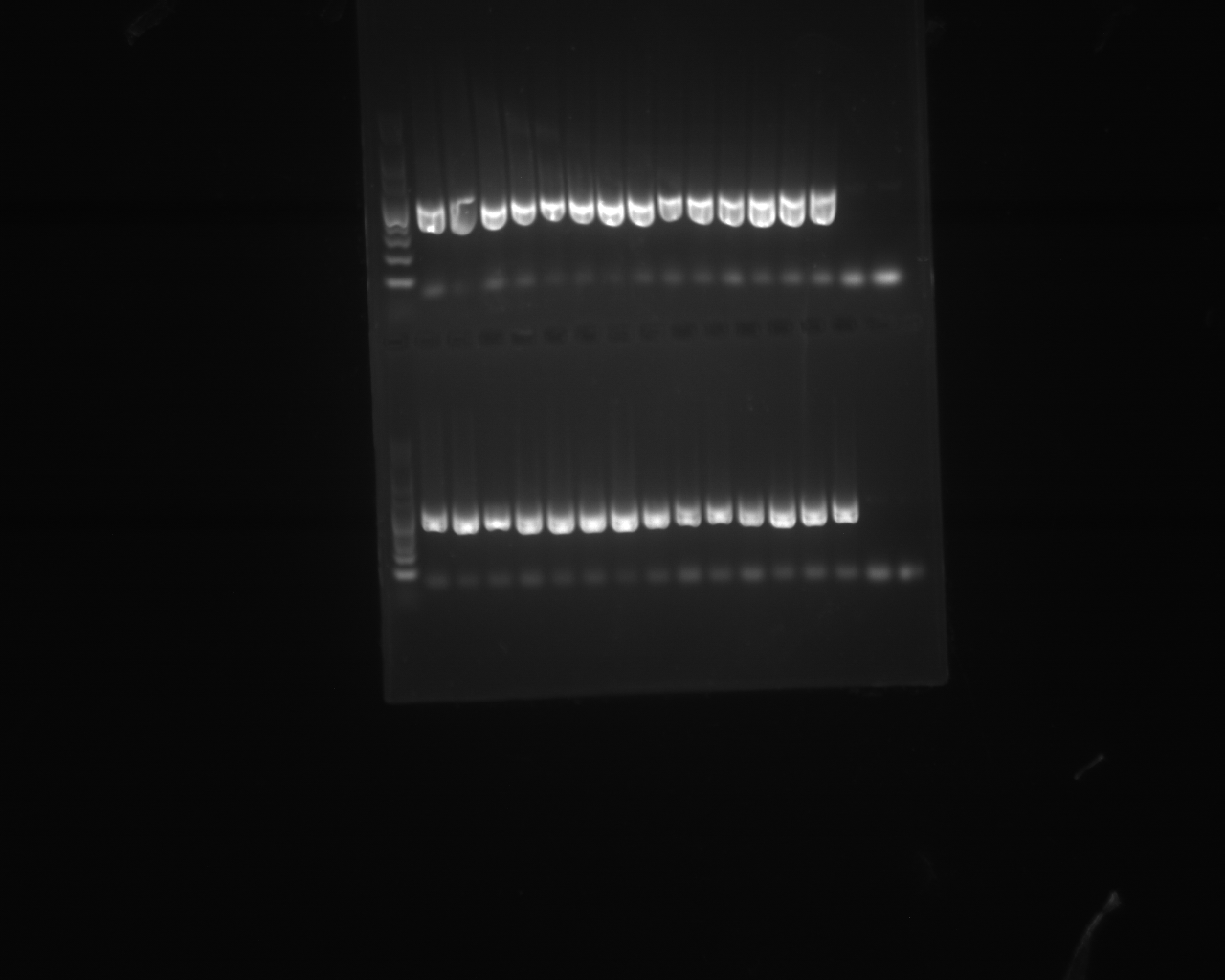

Supplement: Figure 3—source data 2. [file elife-89532-fig3-data2.zip › Figure 3-Source Data 2/E2/Row image/actin confirm.Tif]

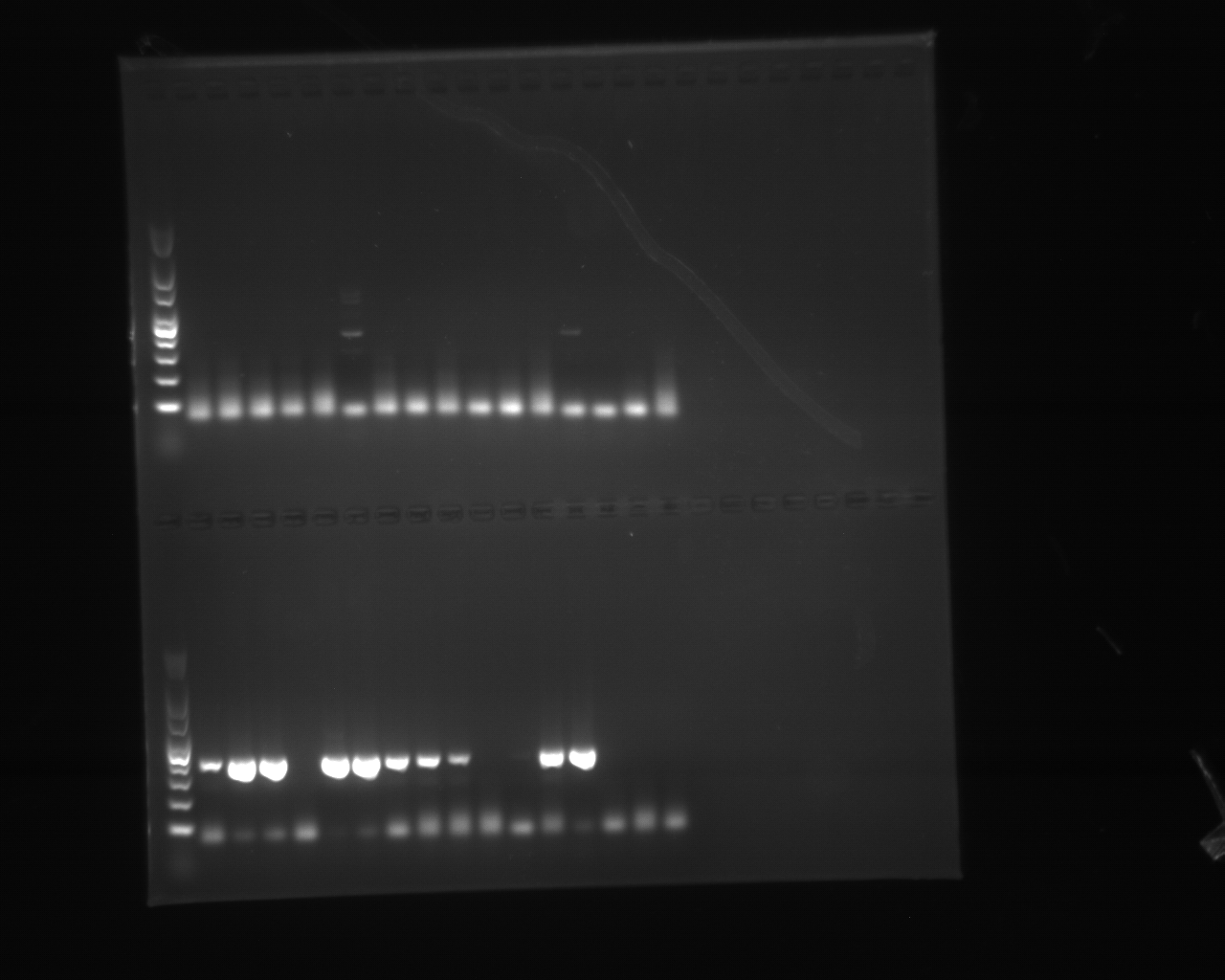

Supplement: Figure 3—source data 2. [file elife-89532-fig3-data2.zip › Figure 3-Source Data 2/E2/Row image/ga1 ga2 .Tif]

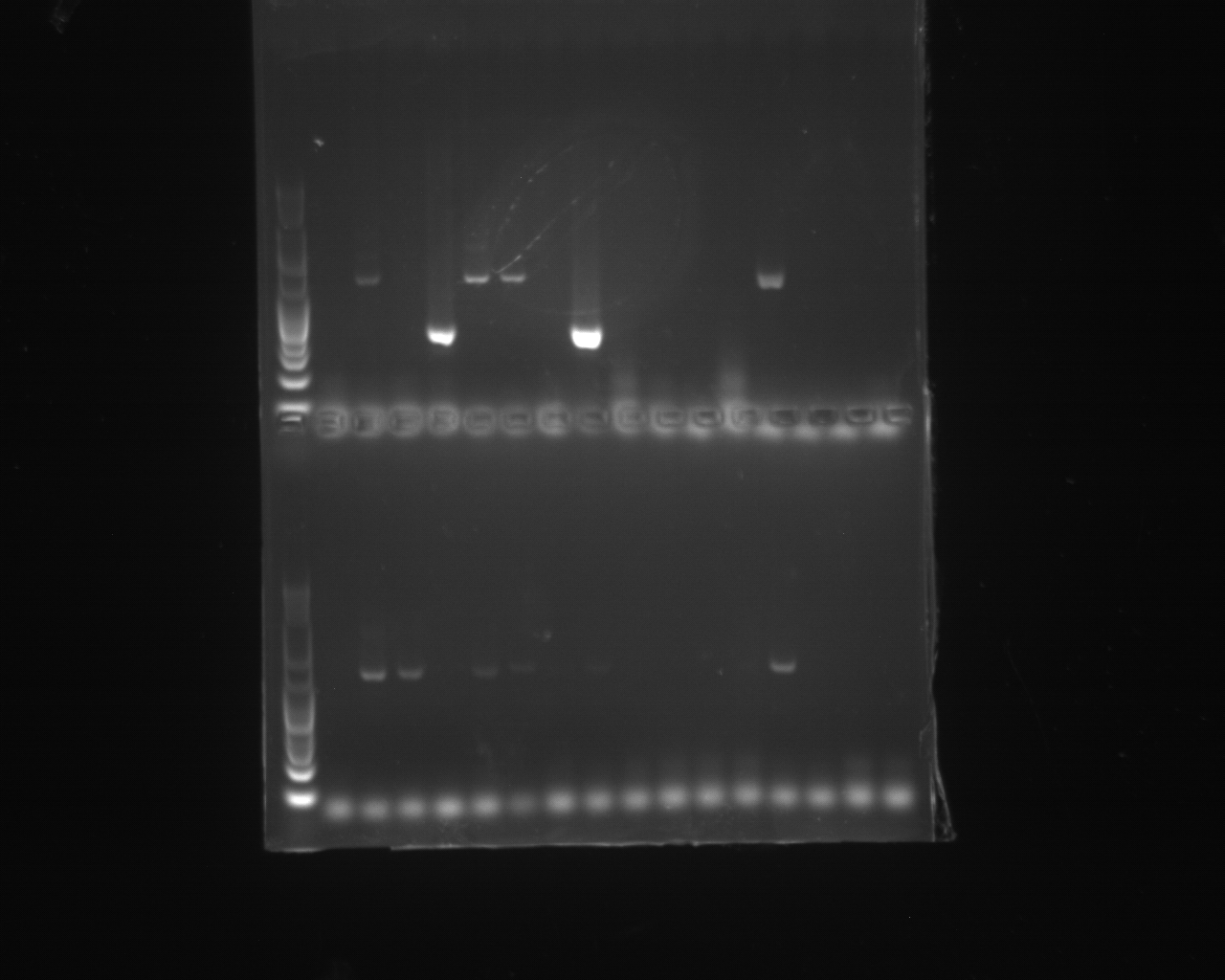

Supplement: Figure 3—source data 2. [file elife-89532-fig3-data2.zip › Figure 3-Source Data 2/E2/Row image/ga11 ga12 .Tif]

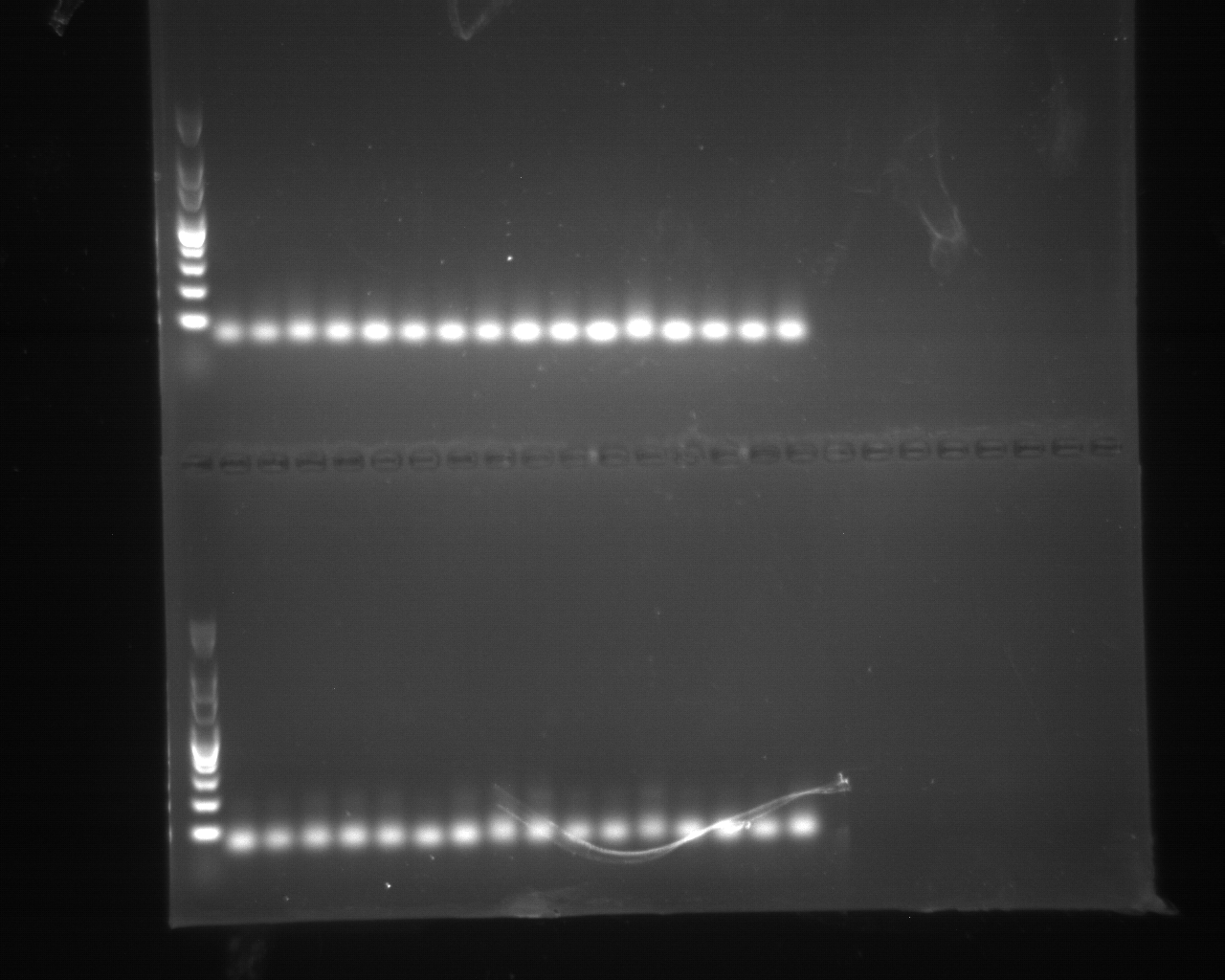

Supplement: Figure 3—source data 2. [file elife-89532-fig3-data2.zip › Figure 3-Source Data 2/E2/Row image/ga3 ga4 .Tif]

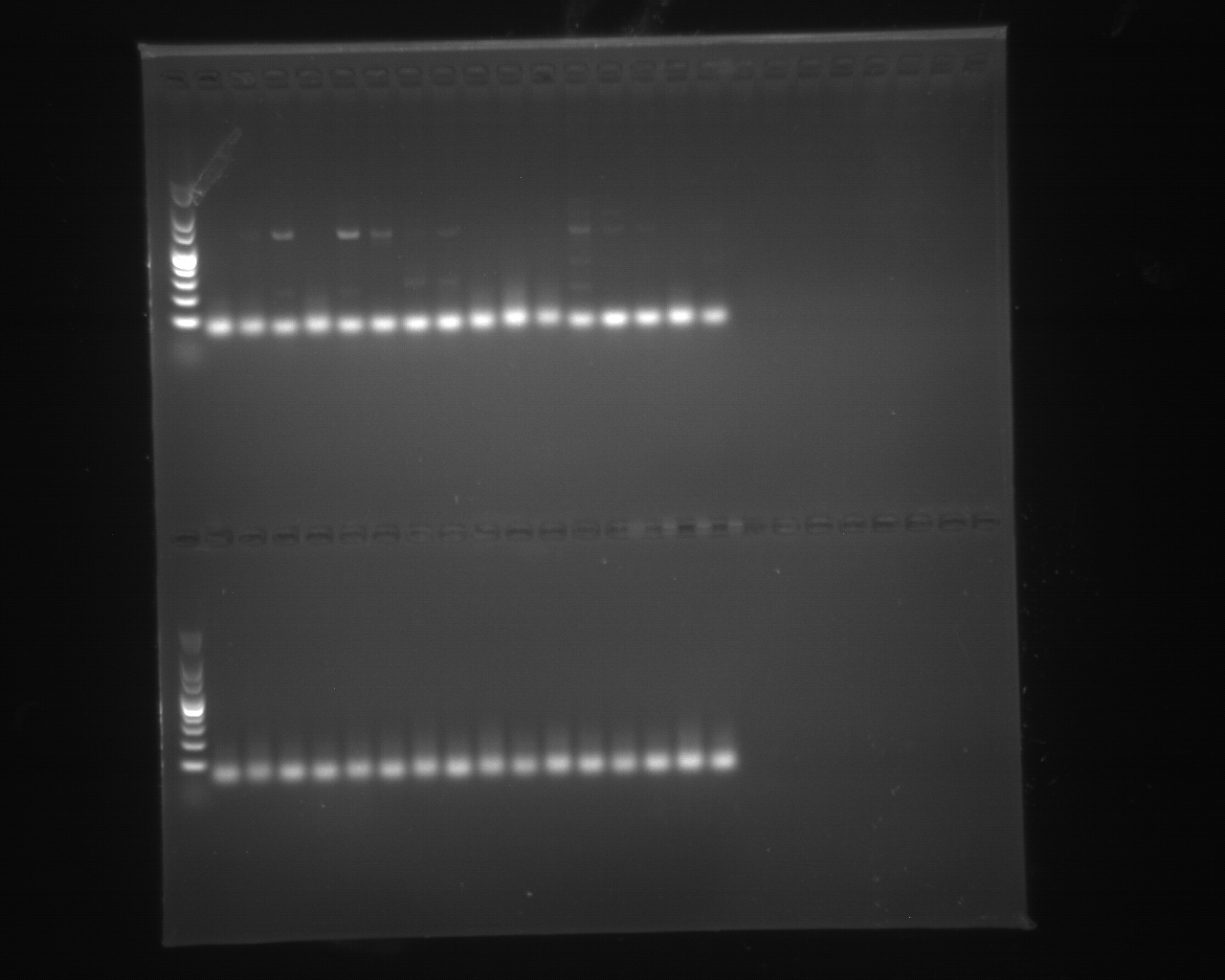

Supplement: Figure 3—source data 2. [file elife-89532-fig3-data2.zip › Figure 3-Source Data 2/E2/Row image/ga5 ga6 .Tif]

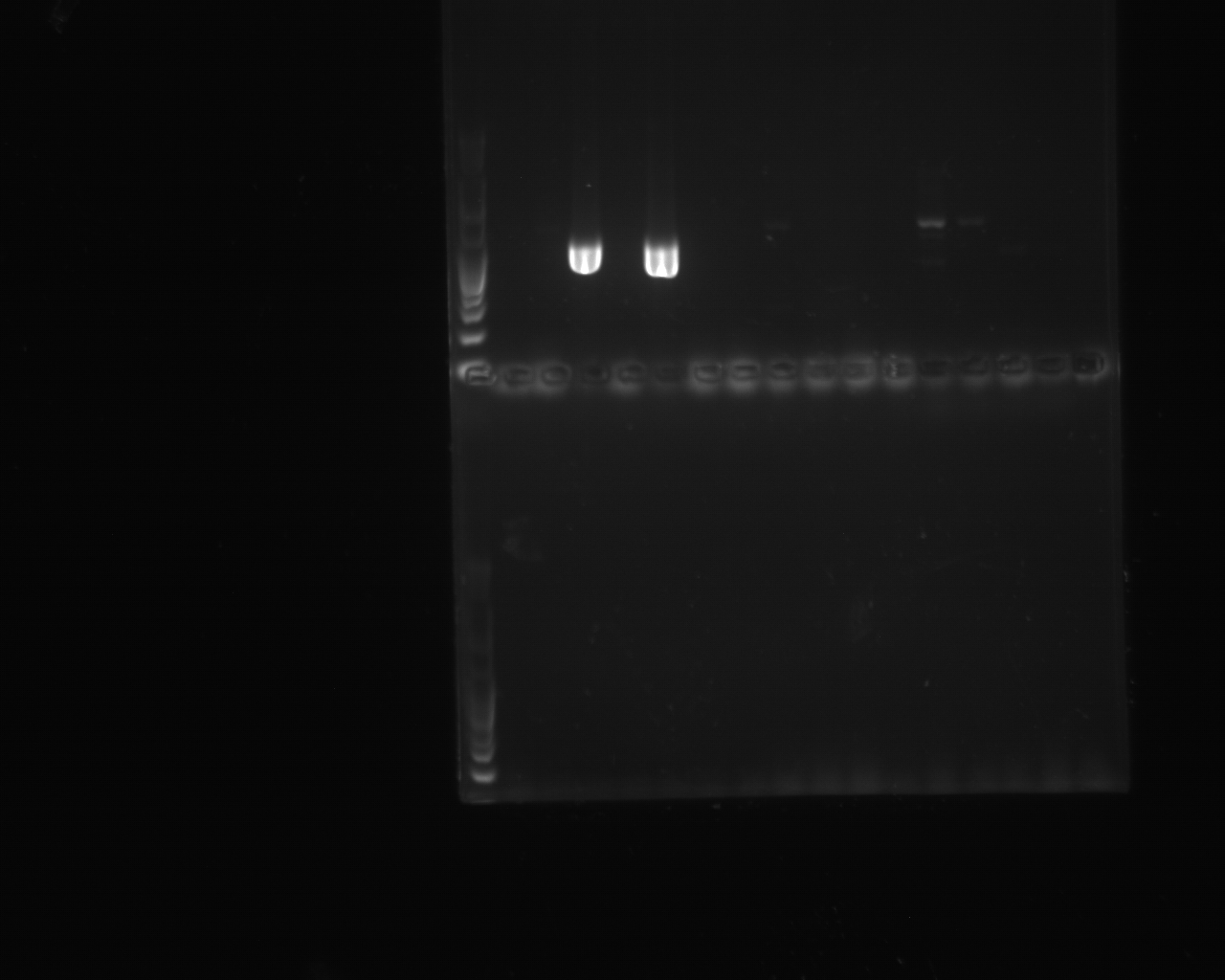

Supplement: Figure 3—source data 2. [file elife-89532-fig3-data2.zip › Figure 3-Source Data 2/E2/Row image/ga7 ga9 .Tif]

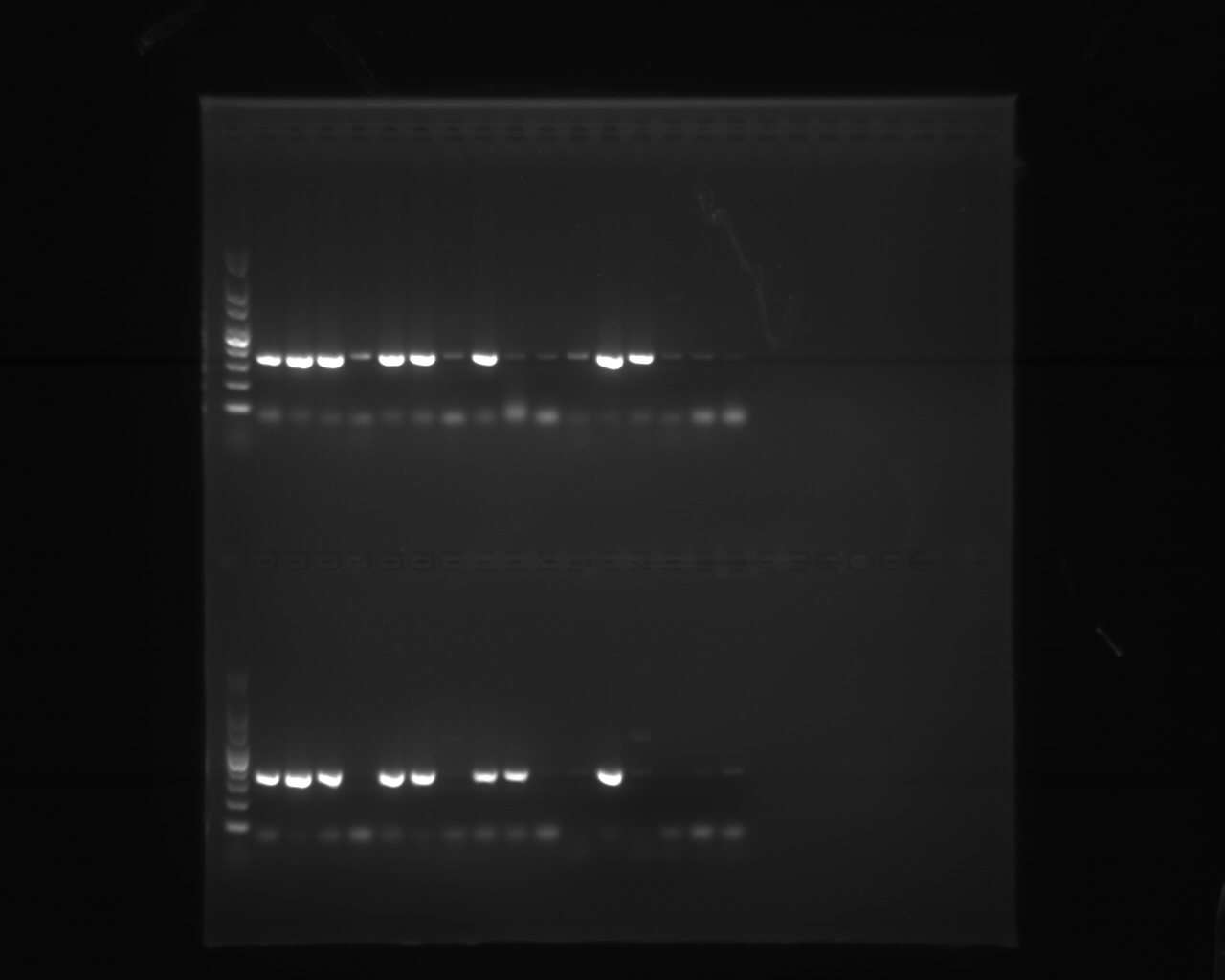

Supplement: Figure 3—source data 2. [file elife-89532-fig3-data2.zip › Figure 3-Source Data 2/E2/Row image/ga8 ga10 .Tif]

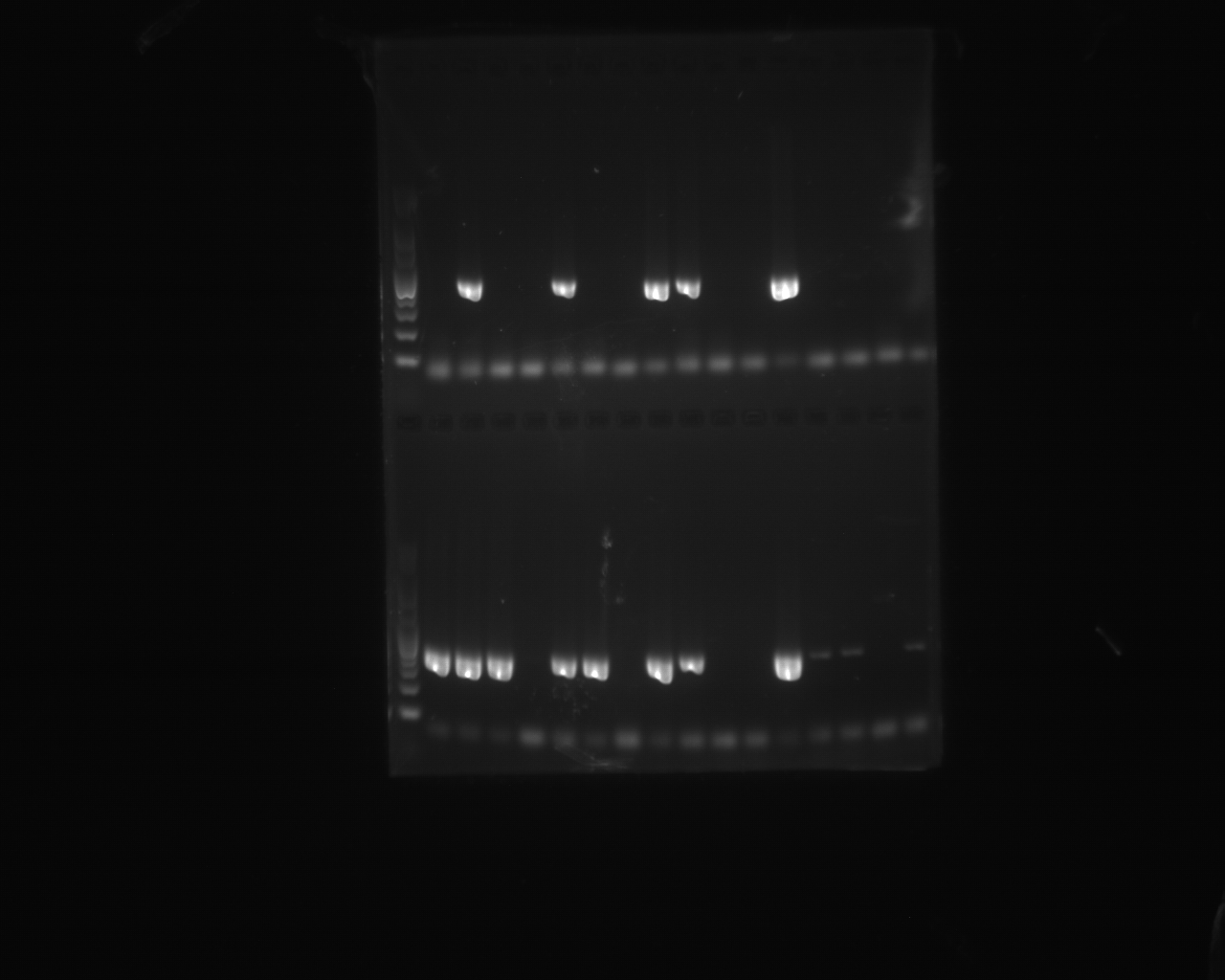

Supplement: Figure 3—source data 2. [file elife-89532-fig3-data2.zip › Figure 3-Source Data 2/E2/Row image/gb1 b2 .Tif]

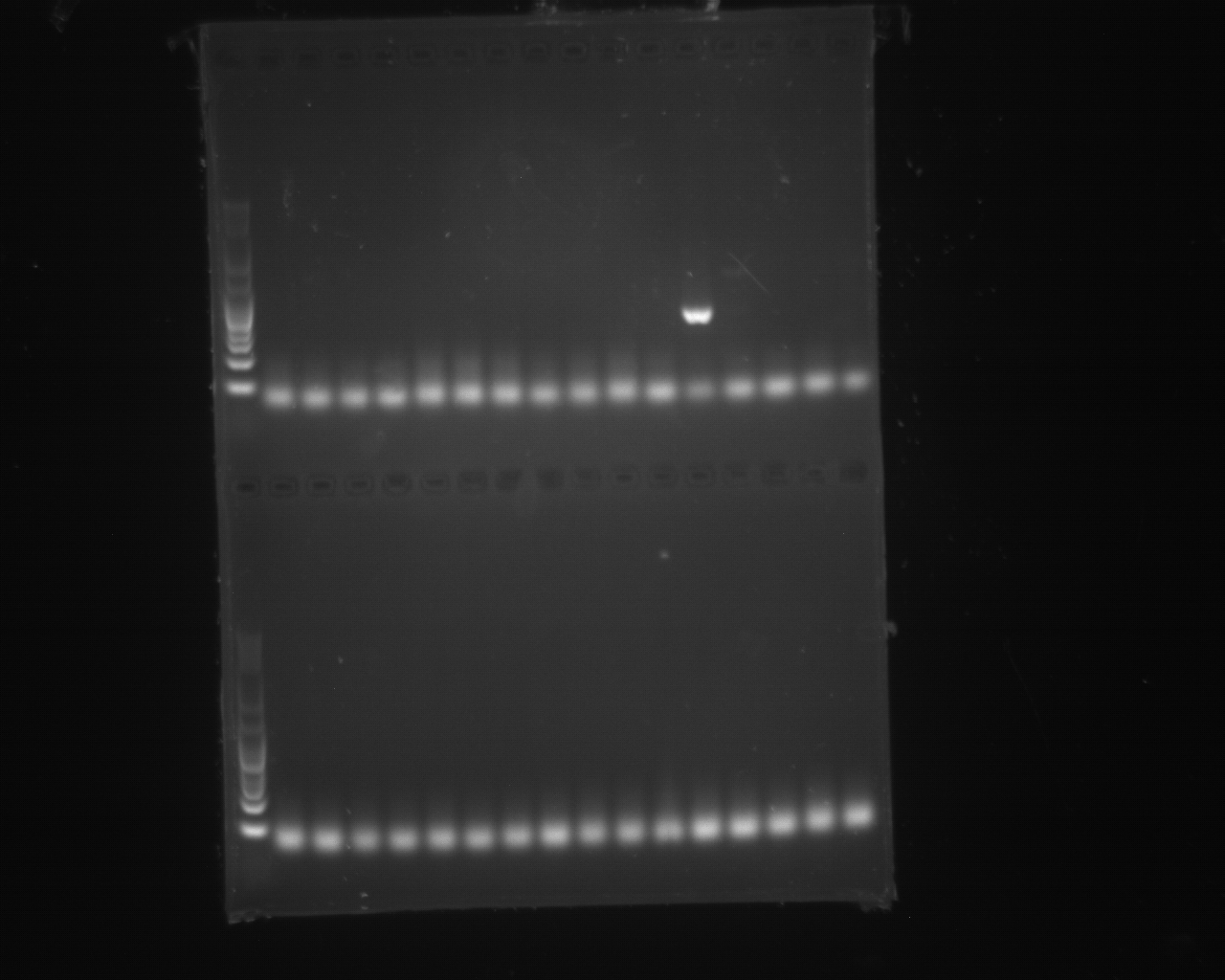

Supplement: Figure 3—source data 2. [file elife-89532-fig3-data2.zip › Figure 3-Source Data 2/E2/Row image/gb4 gb5 .Tif]

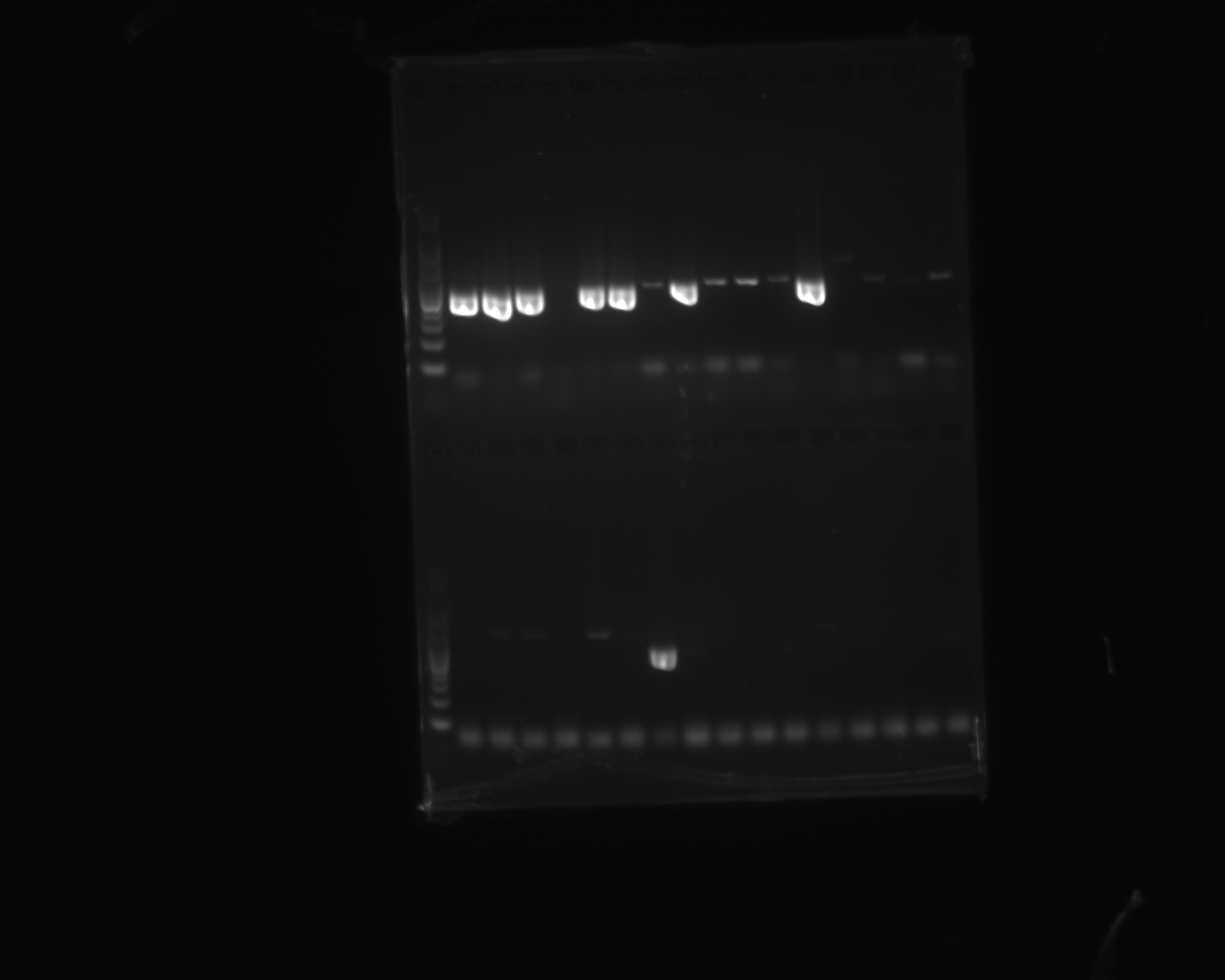

Supplement: Figure 3—source data 2. [file elife-89532-fig3-data2.zip › Figure 3-Source Data 2/E2/Row image/gb6 b7 .Tif]

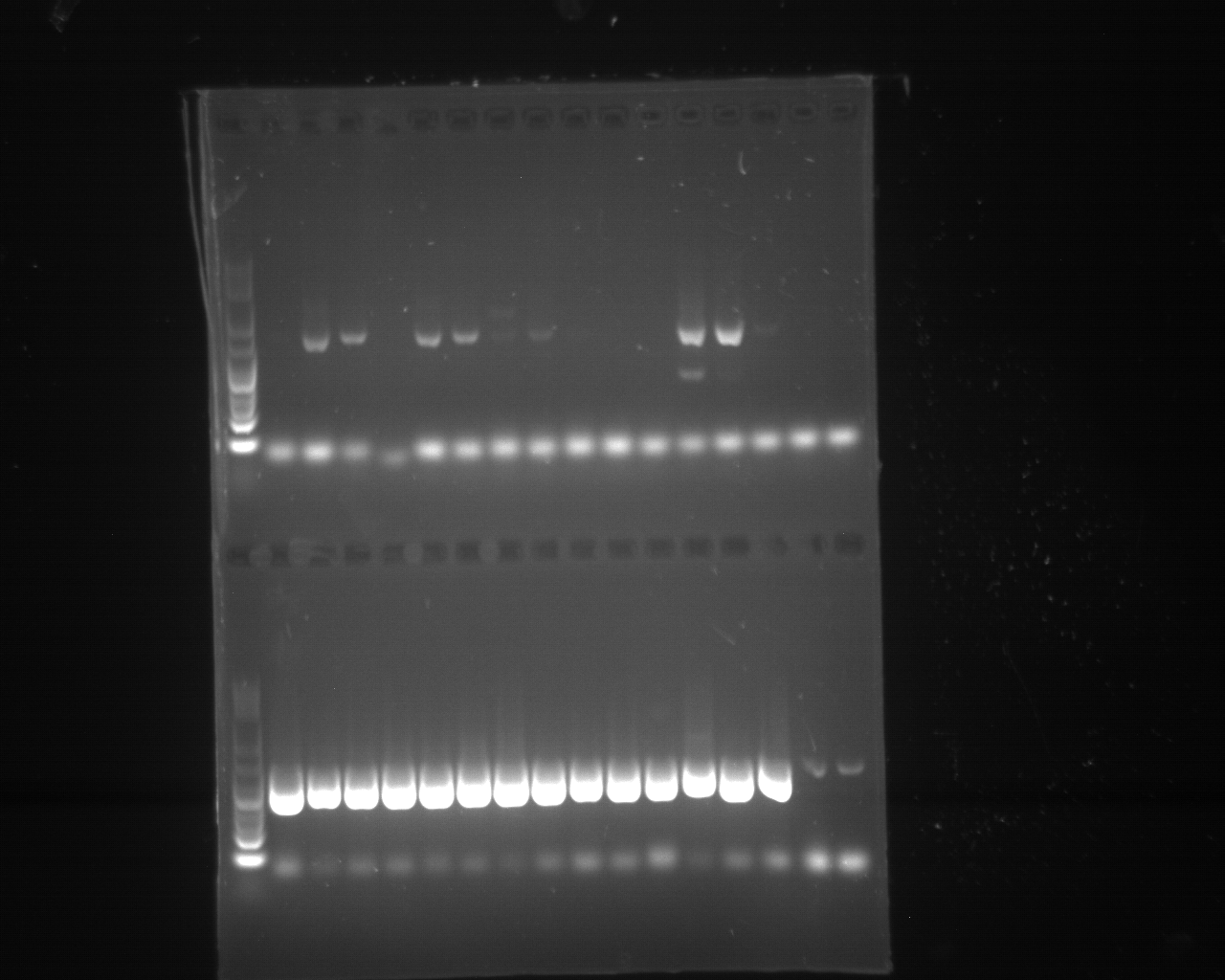

Supplement: Figure 3—source data 2. [file elife-89532-fig3-data2.zip › Figure 3-Source Data 2/E2/Row image/gb8 actin (2).Tif]

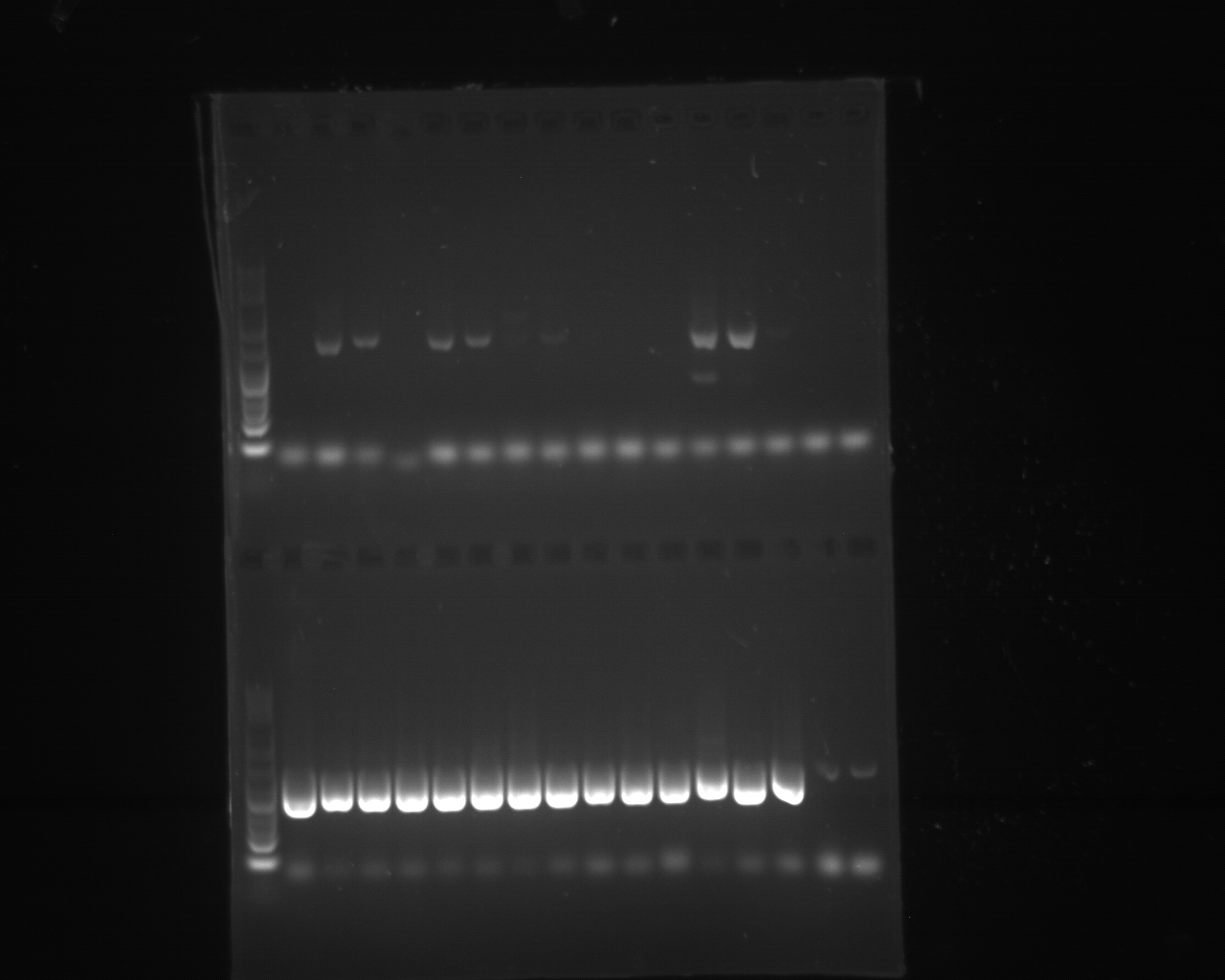

Supplement: Figure 3—source data 2. [file elife-89532-fig3-data2.zip › Figure 3-Source Data 2/E2/Row image/gb8 actin .Tif]

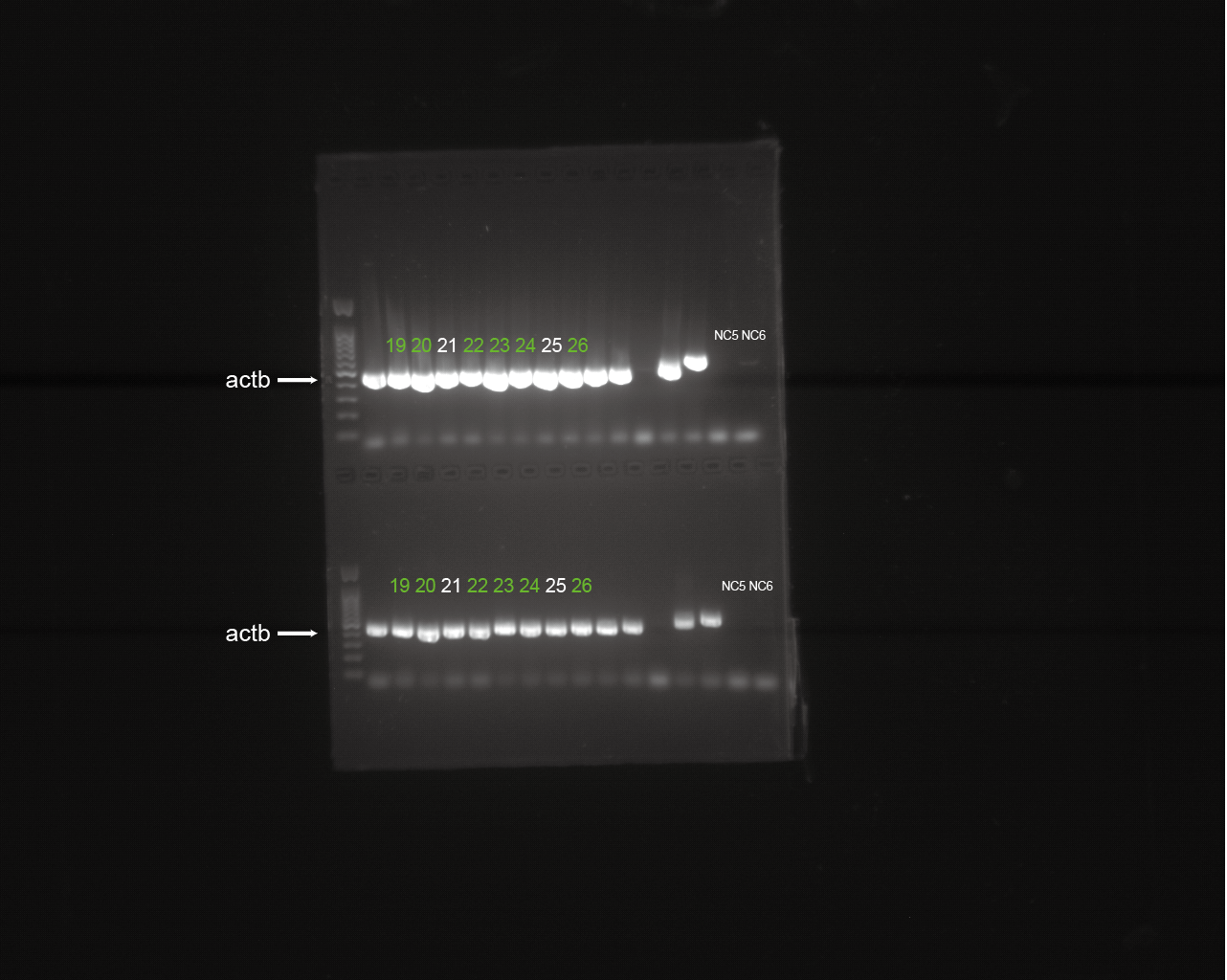

Supplement: Figure 3—source data 2. [file elife-89532-fig3-data2.zip › Figure 3-Source Data 2/E3/labelled image/actin confirm.tif]

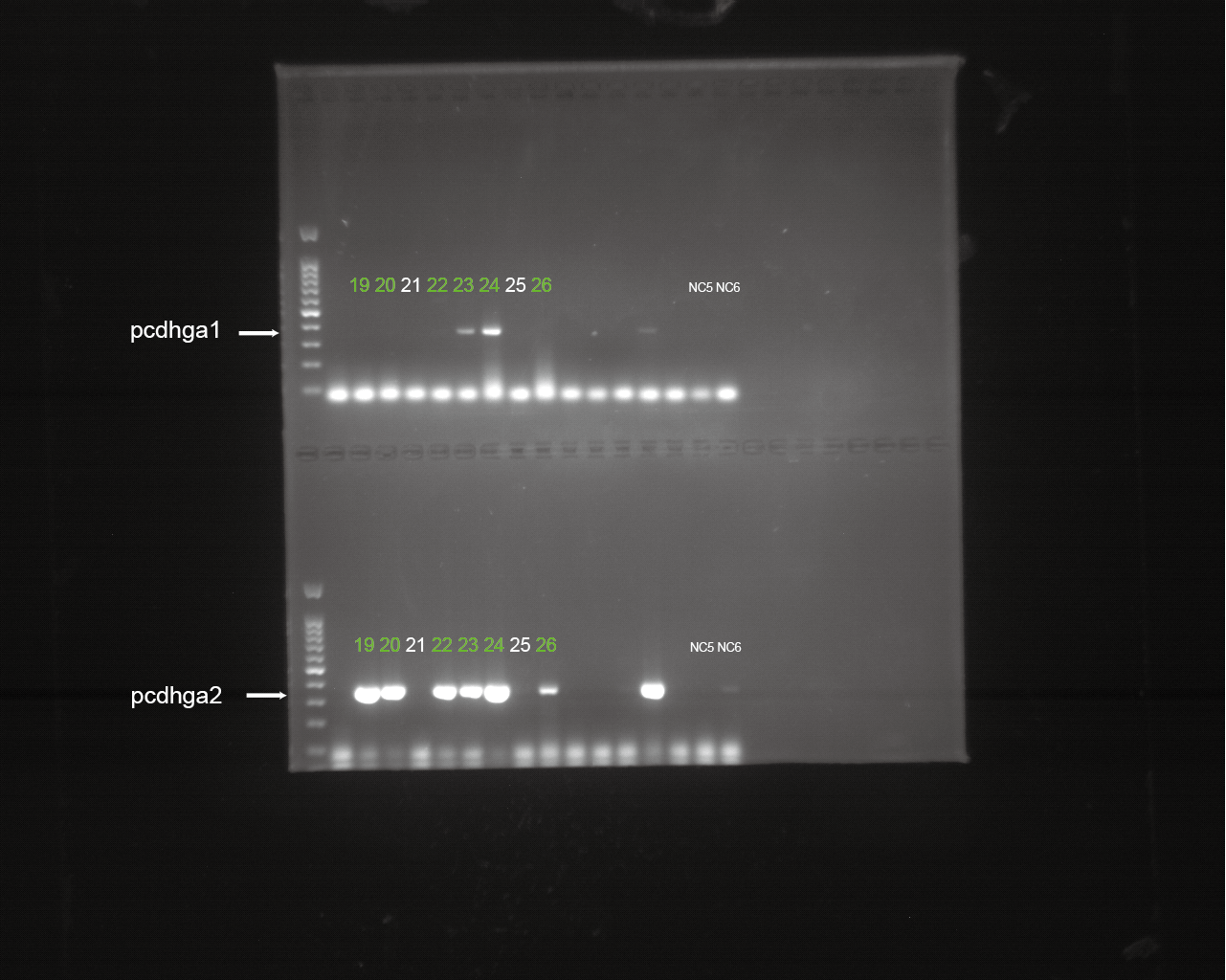

Supplement: Figure 3—source data 2. [file elife-89532-fig3-data2.zip › Figure 3-Source Data 2/E3/labelled image/GA1 GA2 .tif]

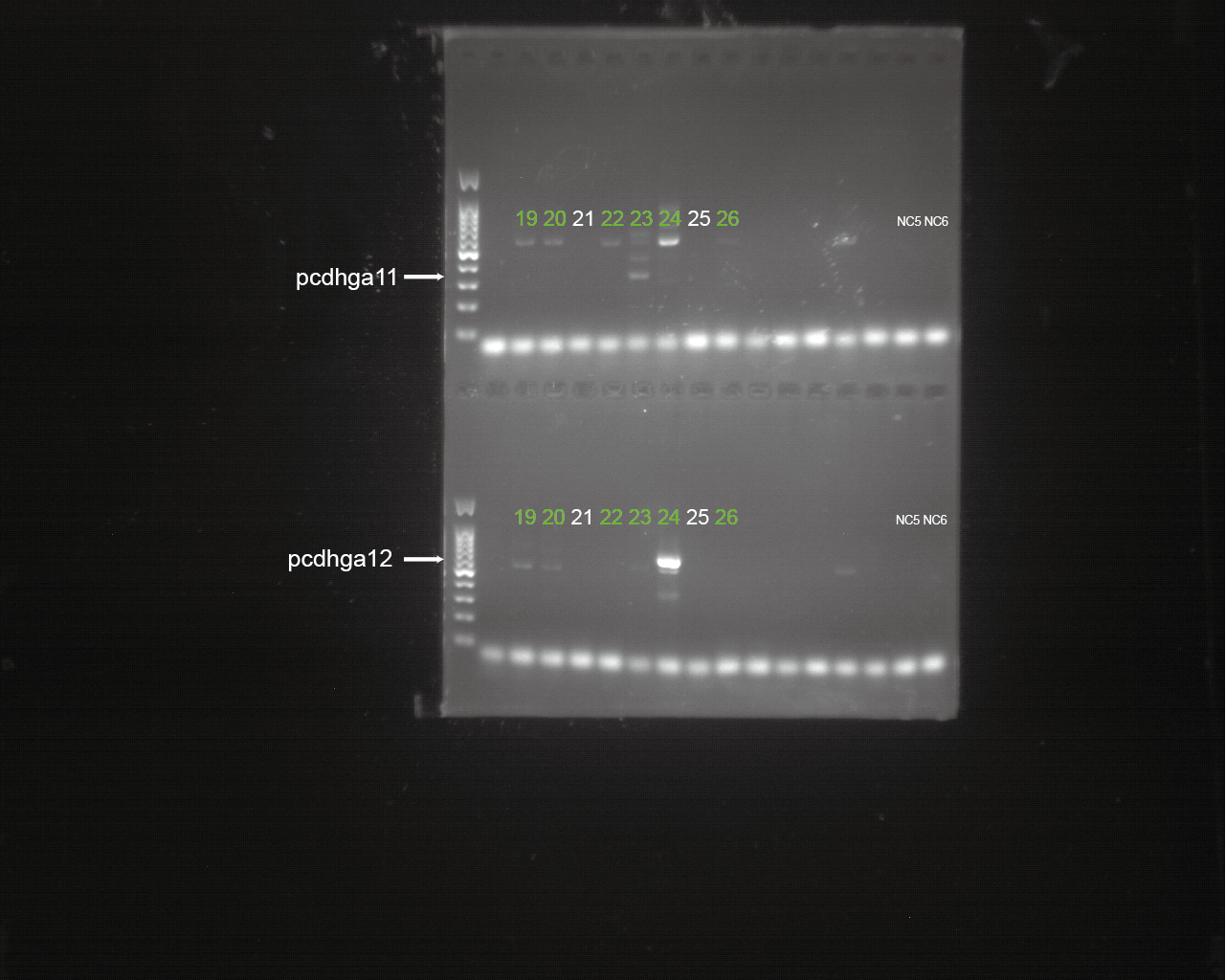

Supplement: Figure 3—source data 2. [file elife-89532-fig3-data2.zip › Figure 3-Source Data 2/E3/labelled image/Ga11 Ga12 .tif]

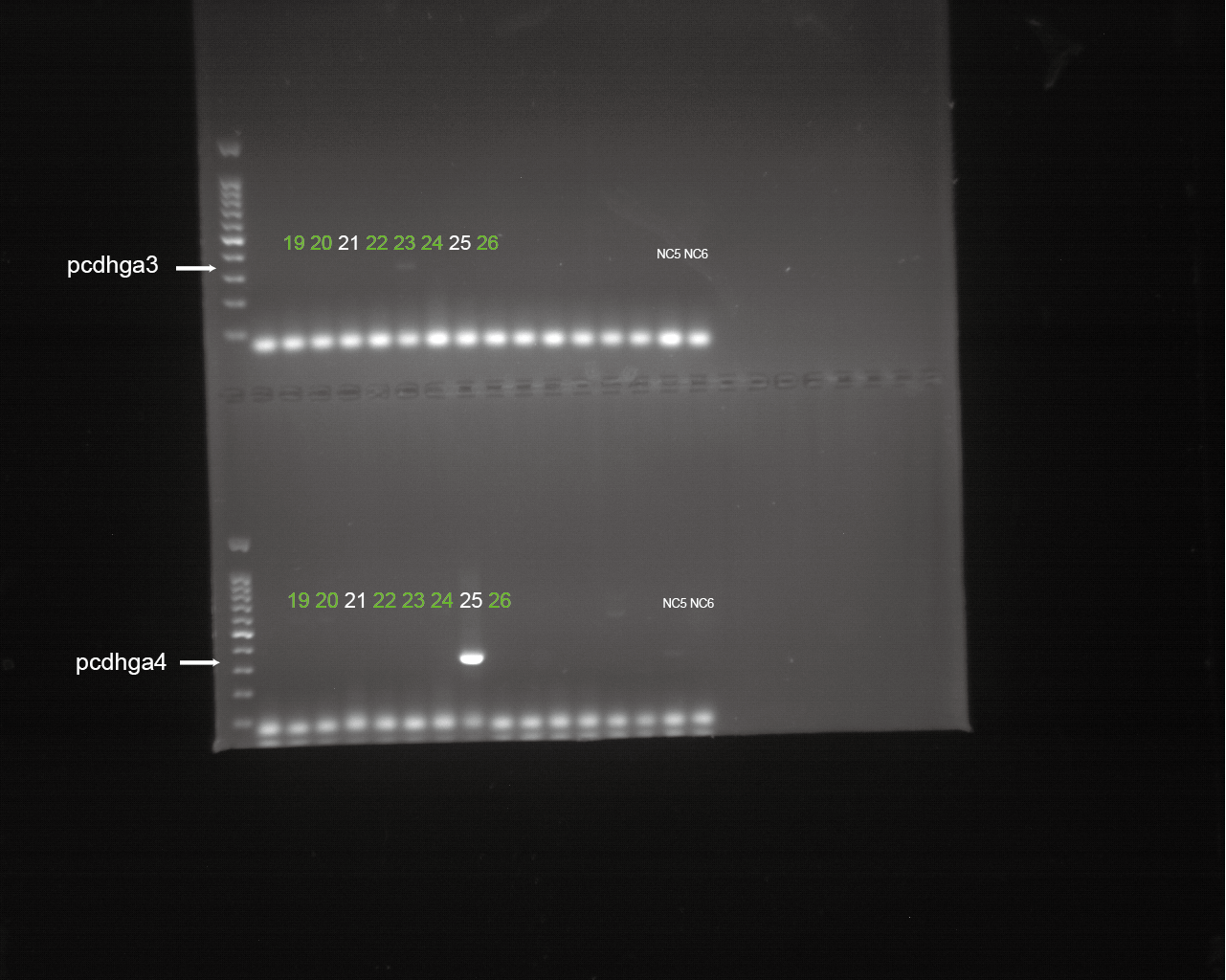

Supplement: Figure 3—source data 2. [file elife-89532-fig3-data2.zip › Figure 3-Source Data 2/E3/labelled image/Ga3 Ga4 .tif]

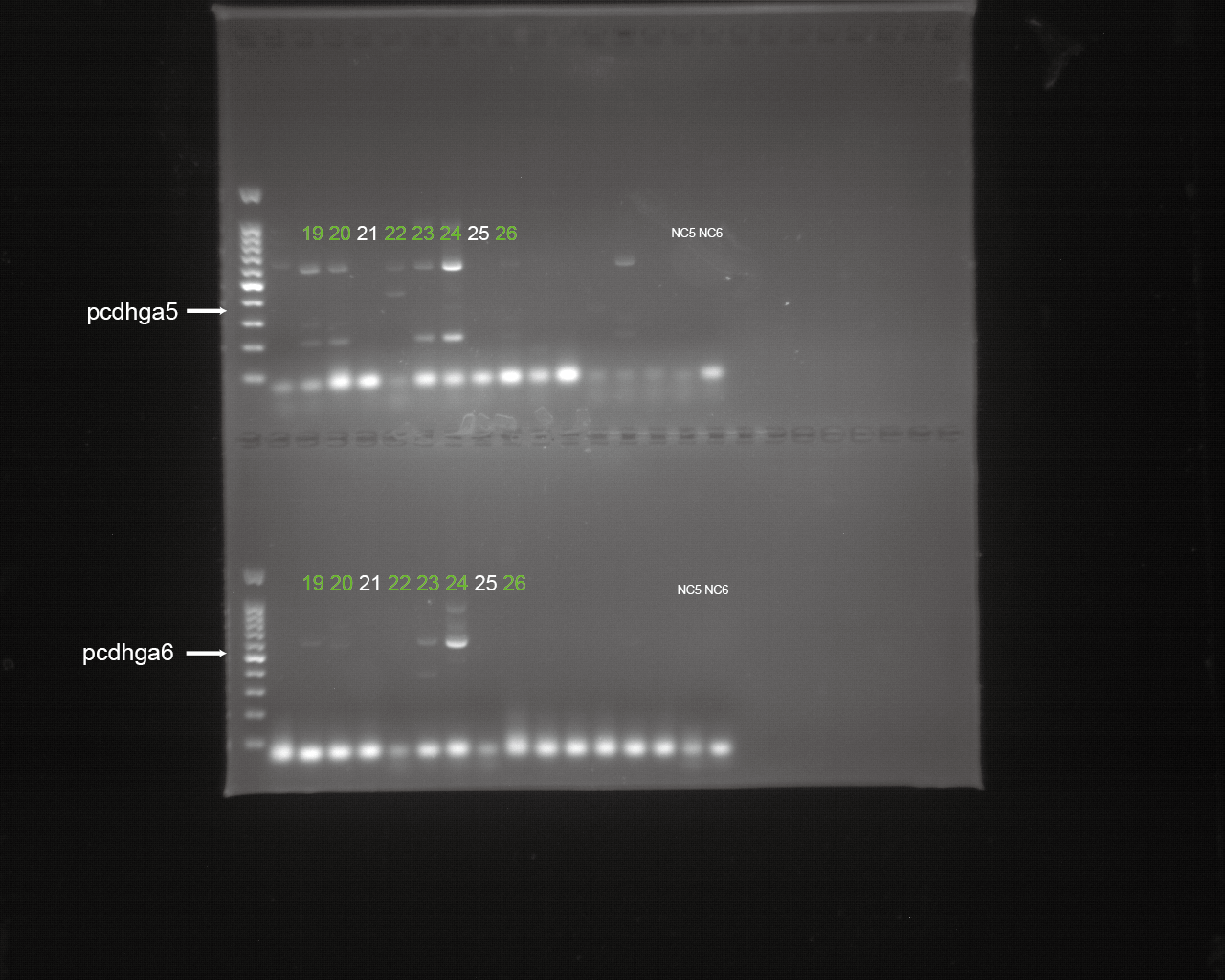

Supplement: Figure 3—source data 2. [file elife-89532-fig3-data2.zip › Figure 3-Source Data 2/E3/labelled image/Ga5 Ga6 .tif]

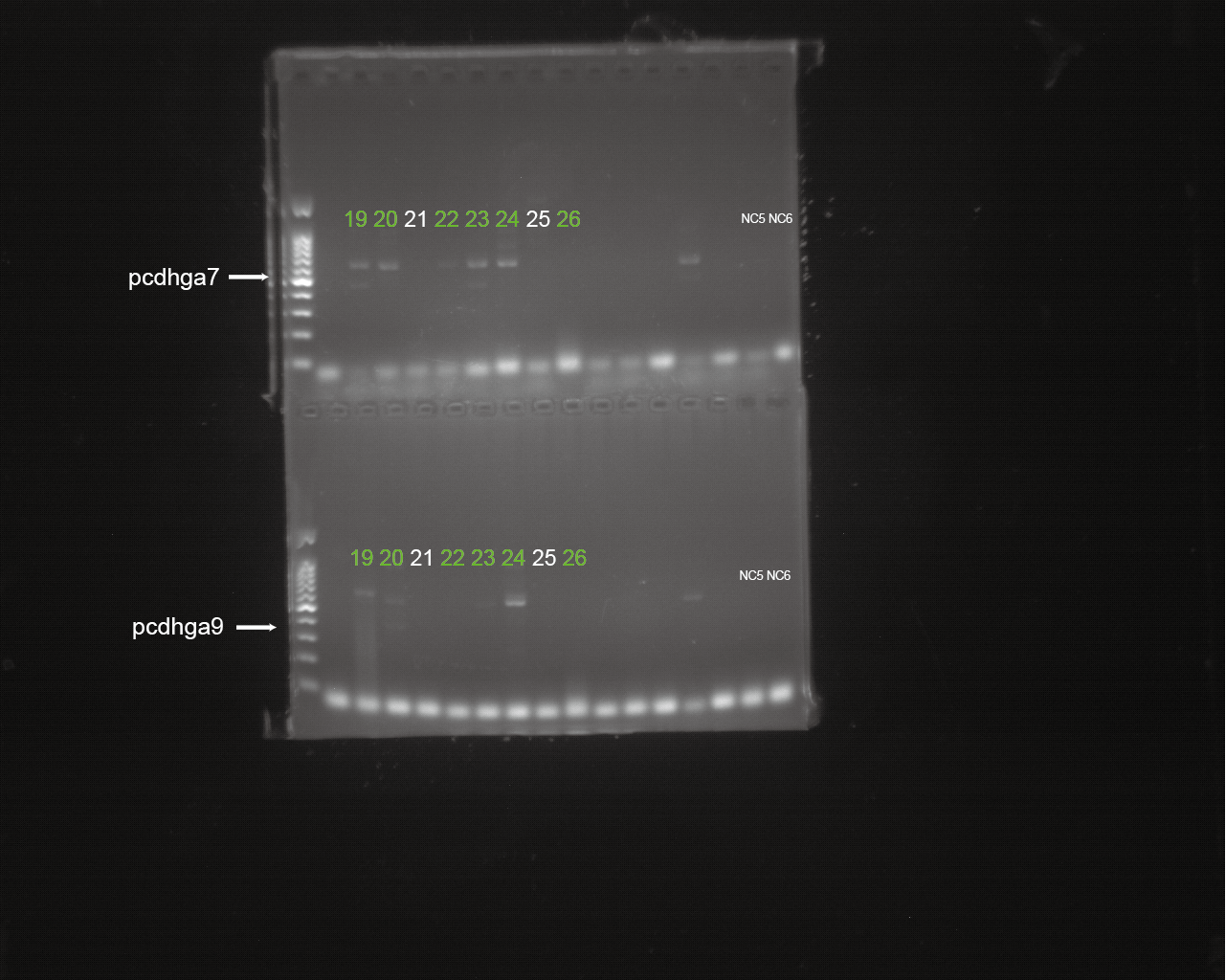

Supplement: Figure 3—source data 2. [file elife-89532-fig3-data2.zip › Figure 3-Source Data 2/E3/labelled image/Ga7 Ga9 .tif]

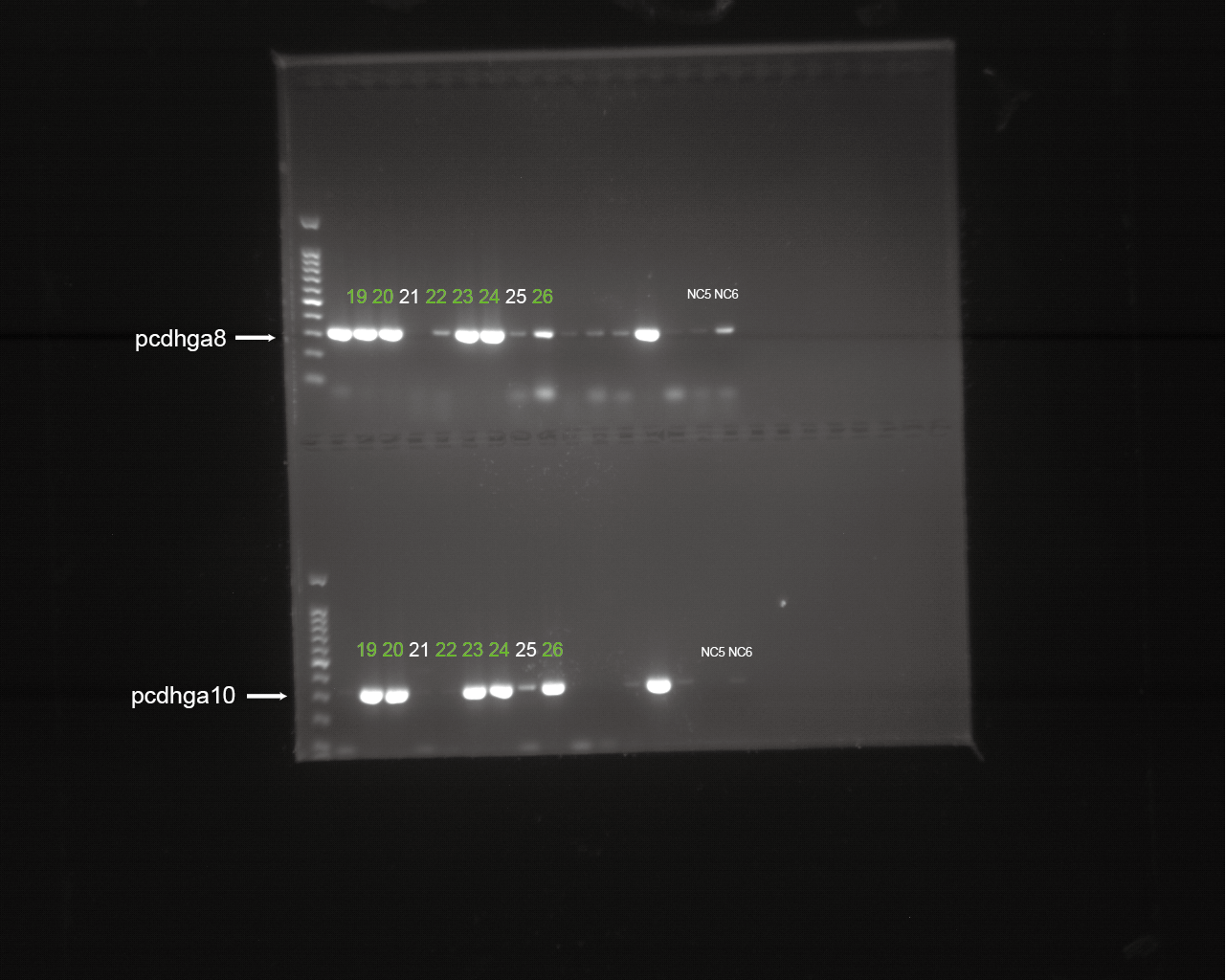

Supplement: Figure 3—source data 2. [file elife-89532-fig3-data2.zip › Figure 3-Source Data 2/E3/labelled image/GA8 GA10 .tif]

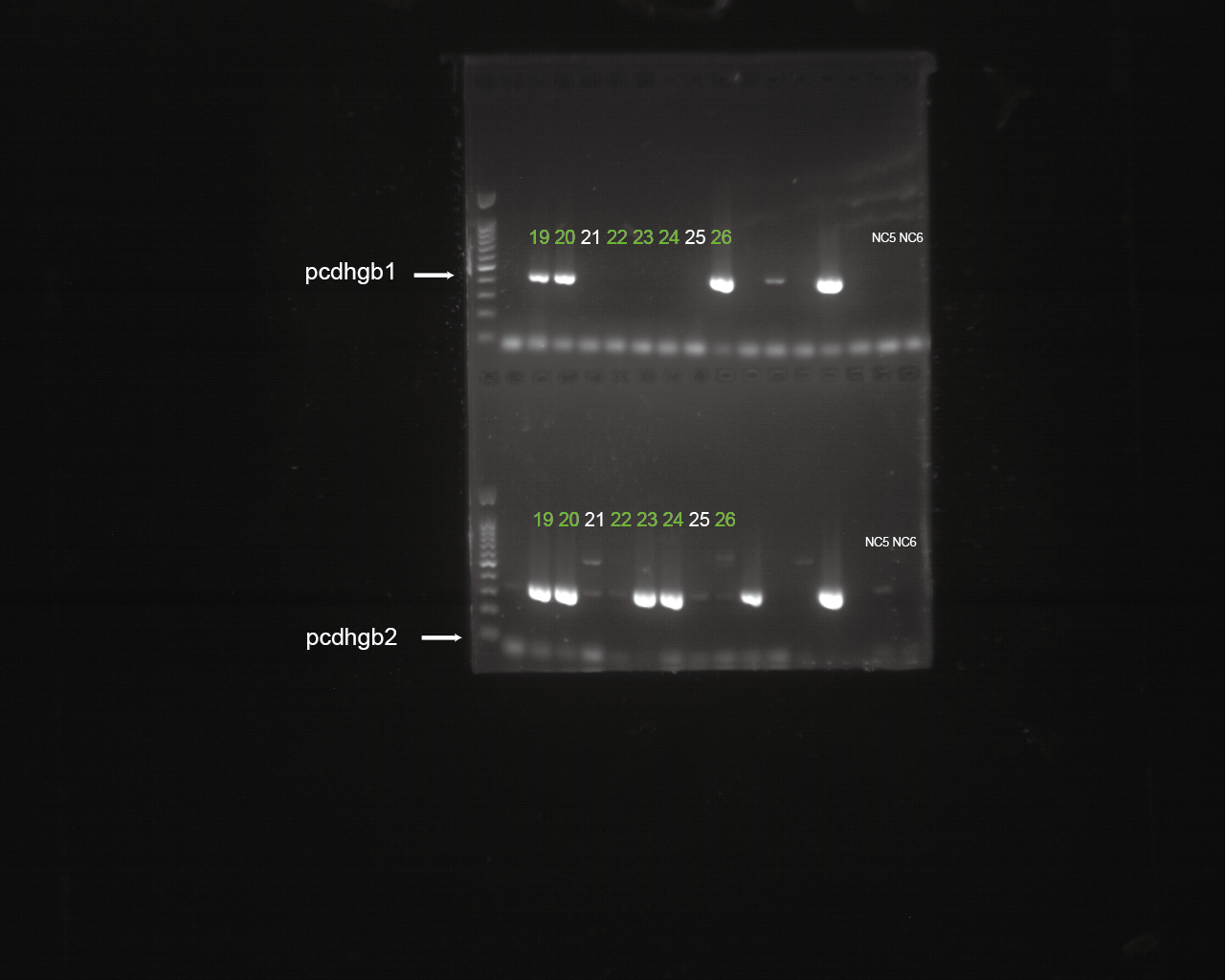

Supplement: Figure 3—source data 2. [file elife-89532-fig3-data2.zip › Figure 3-Source Data 2/E3/labelled image/GB1 GB2 .tif]

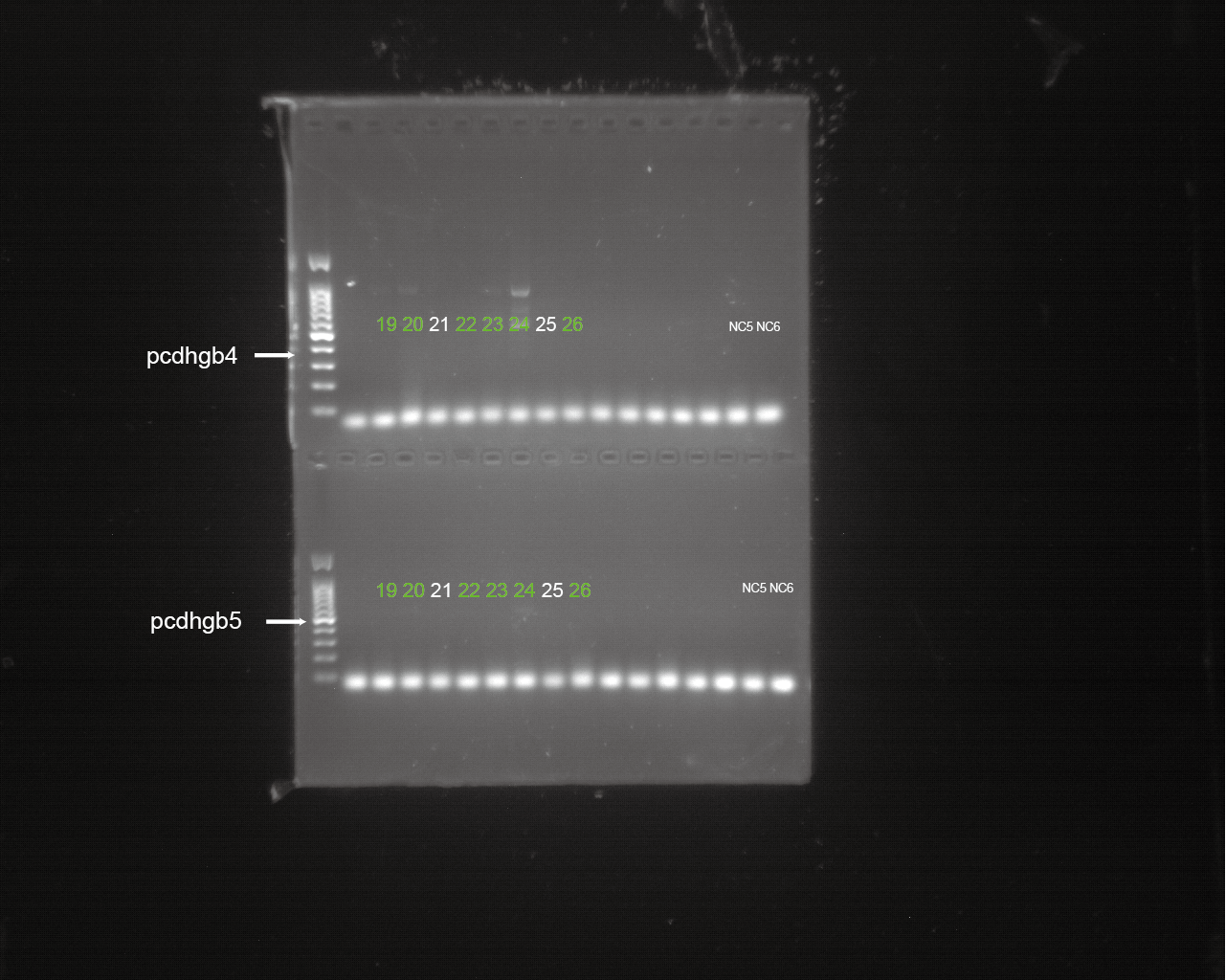

Supplement: Figure 3—source data 2. [file elife-89532-fig3-data2.zip › Figure 3-Source Data 2/E3/labelled image/Gb4 Gb5 .tif]

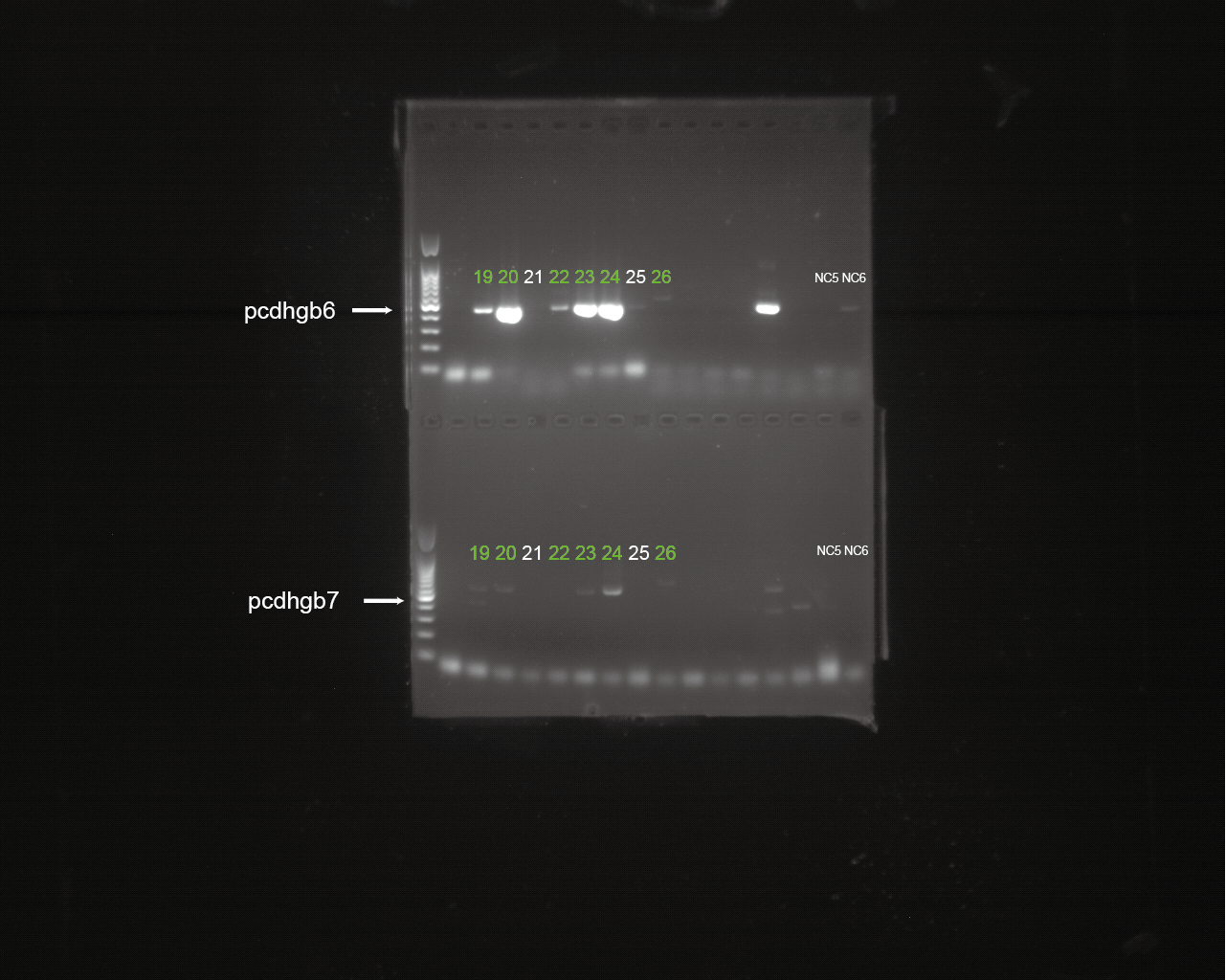

Supplement: Figure 3—source data 2. [file elife-89532-fig3-data2.zip › Figure 3-Source Data 2/E3/labelled image/GB6 GB7 .tif]

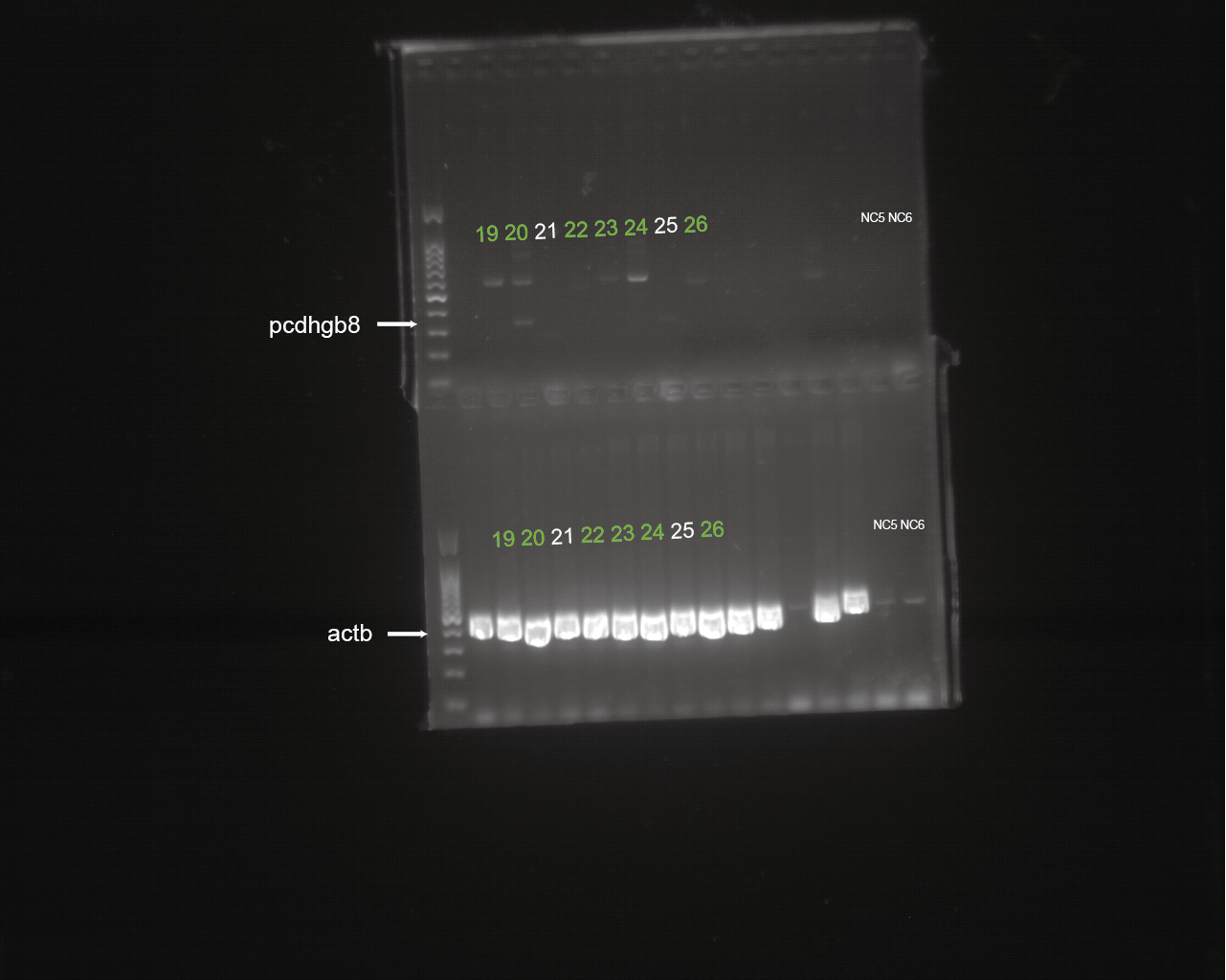

Supplement: Figure 3—source data 2. [file elife-89532-fig3-data2.zip › Figure 3-Source Data 2/E3/labelled image/Gb8 actin (2).tif]

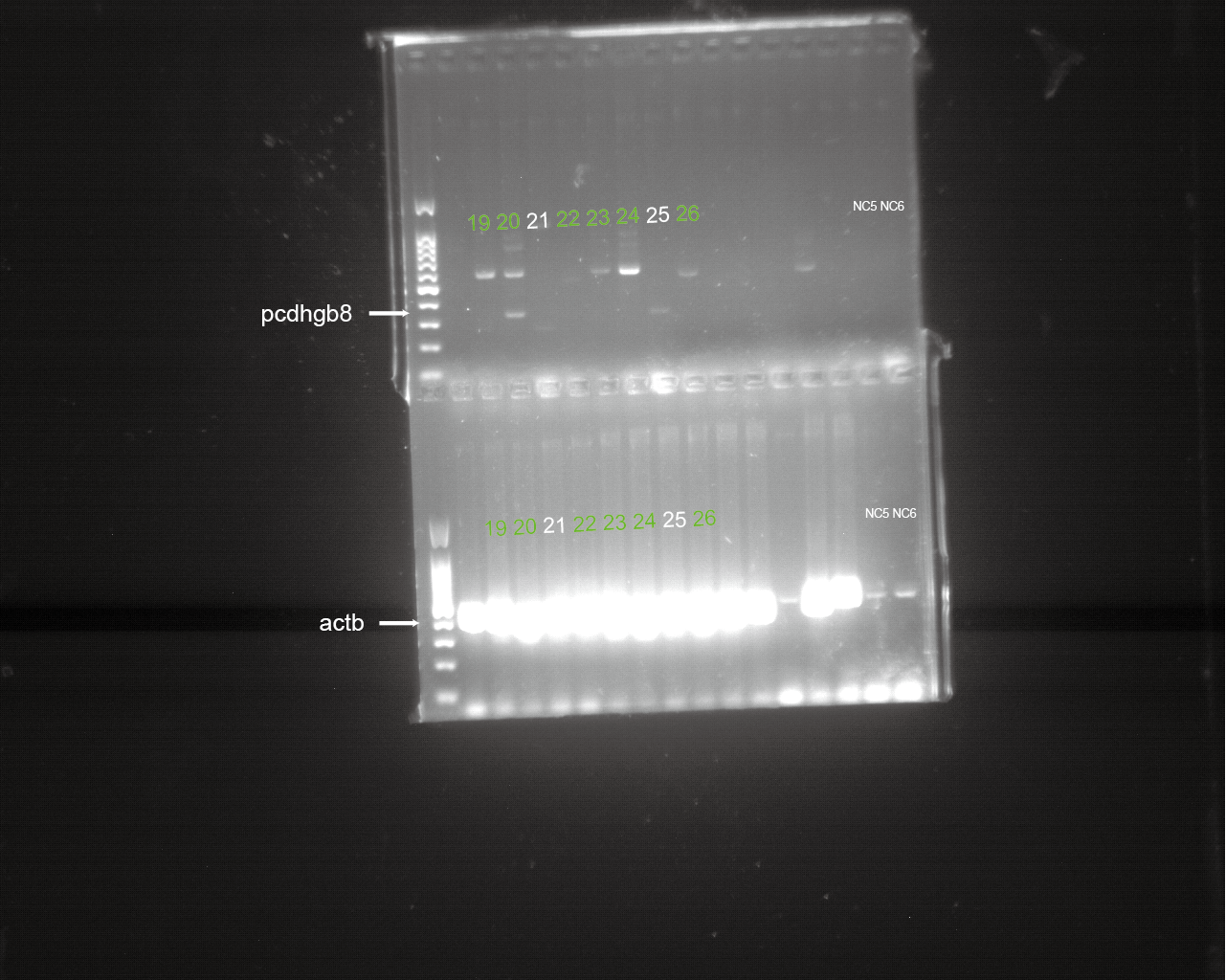

Supplement: Figure 3—source data 2. [file elife-89532-fig3-data2.zip › Figure 3-Source Data 2/E3/labelled image/Gb8 actin .tif]

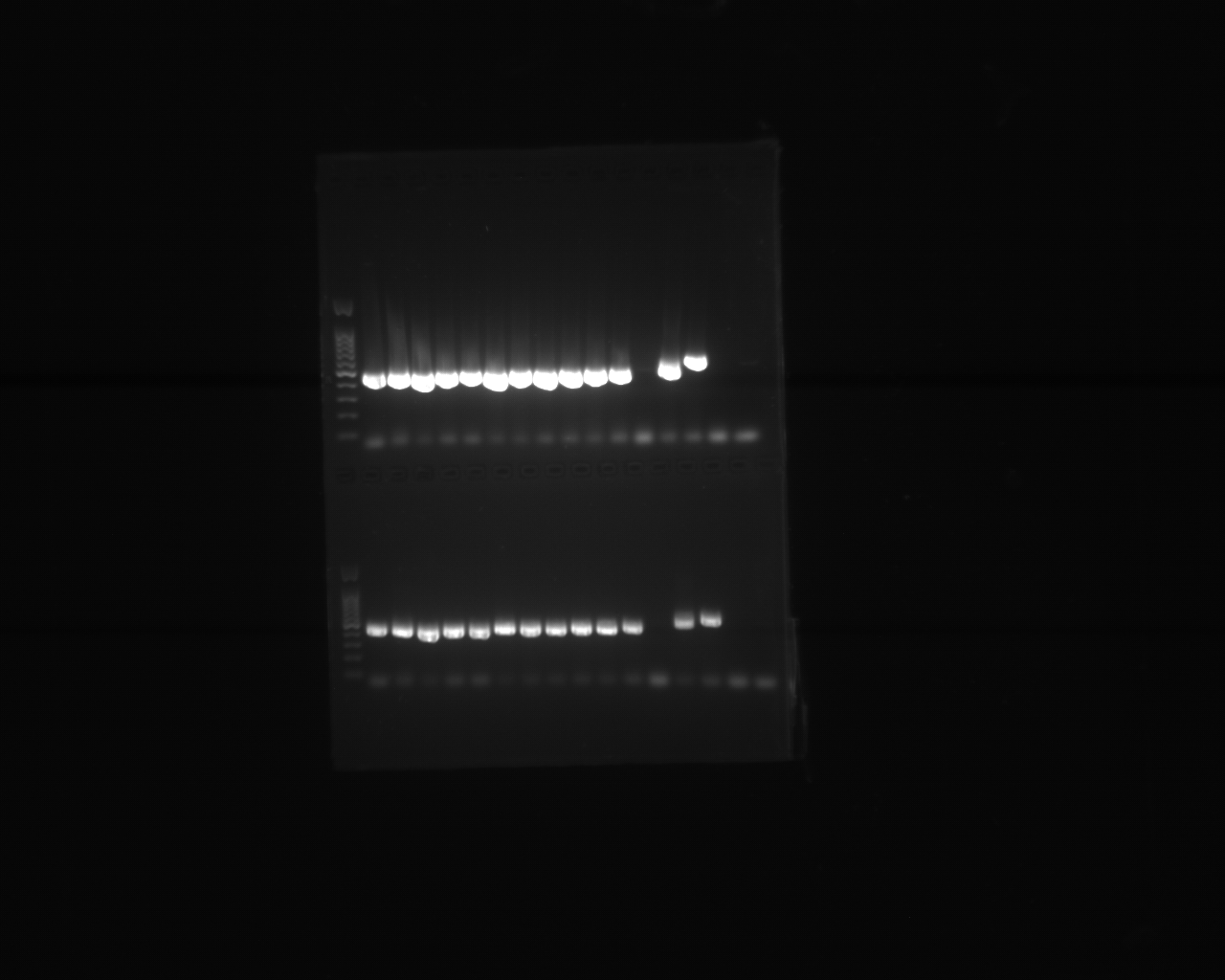

Supplement: Figure 3—source data 2. [file elife-89532-fig3-data2.zip › Figure 3-Source Data 2/E3/Row image/actin confirm.Tif]

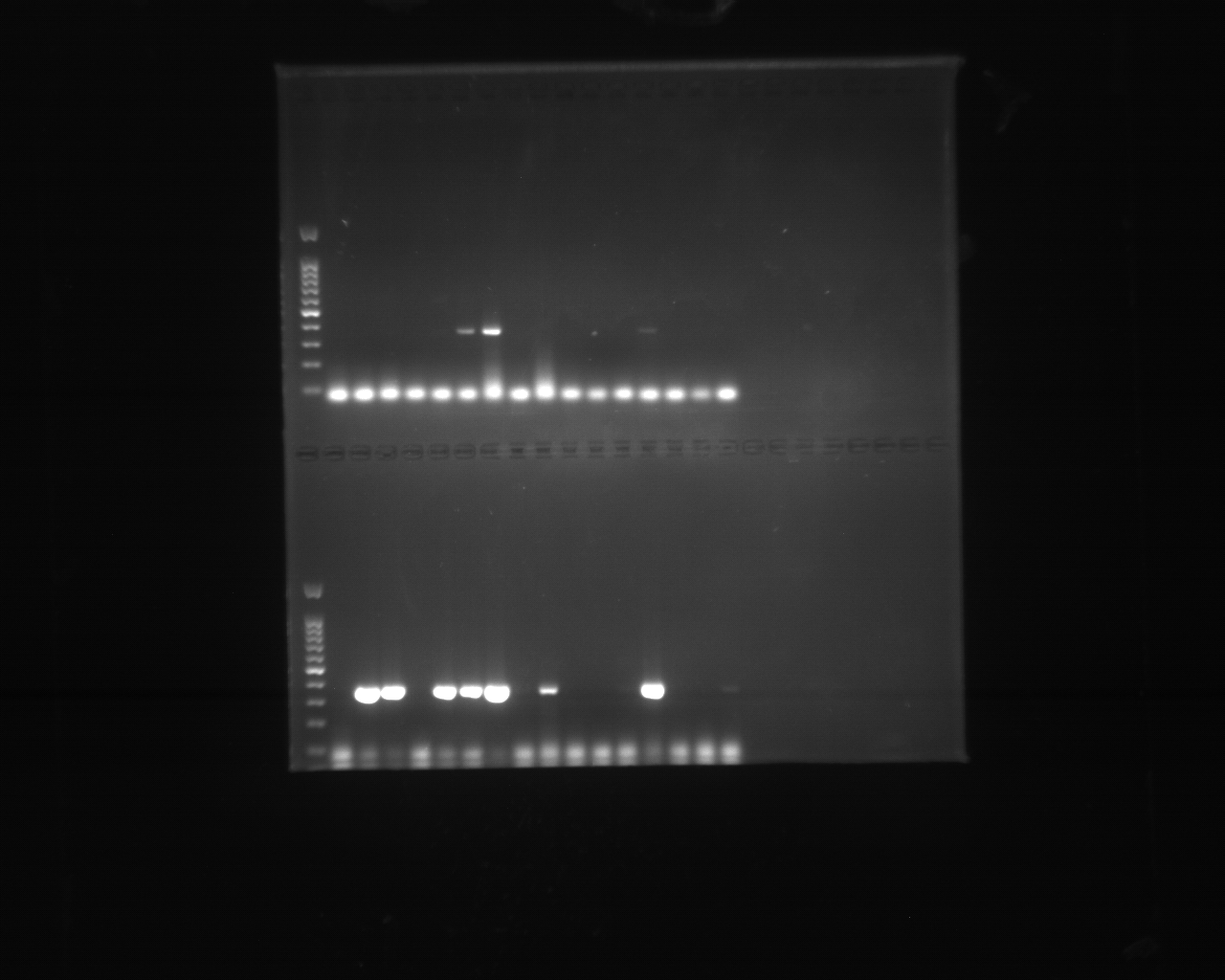

Supplement: Figure 3—source data 2. [file elife-89532-fig3-data2.zip › Figure 3-Source Data 2/E3/Row image/GA1 GA2 .Tif]

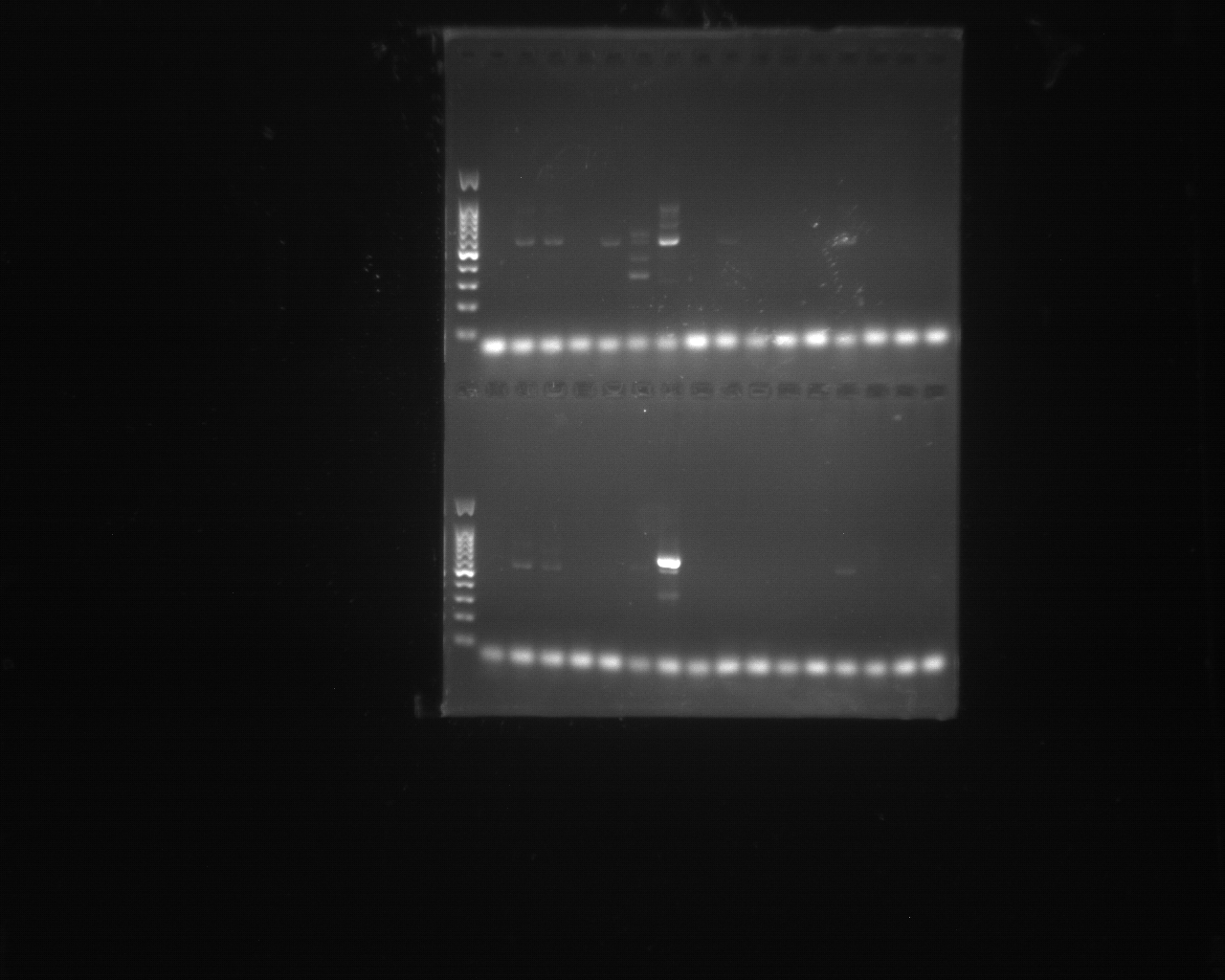

Supplement: Figure 3—source data 2. [file elife-89532-fig3-data2.zip › Figure 3-Source Data 2/E3/Row image/Ga11 Ga12 .Tif]

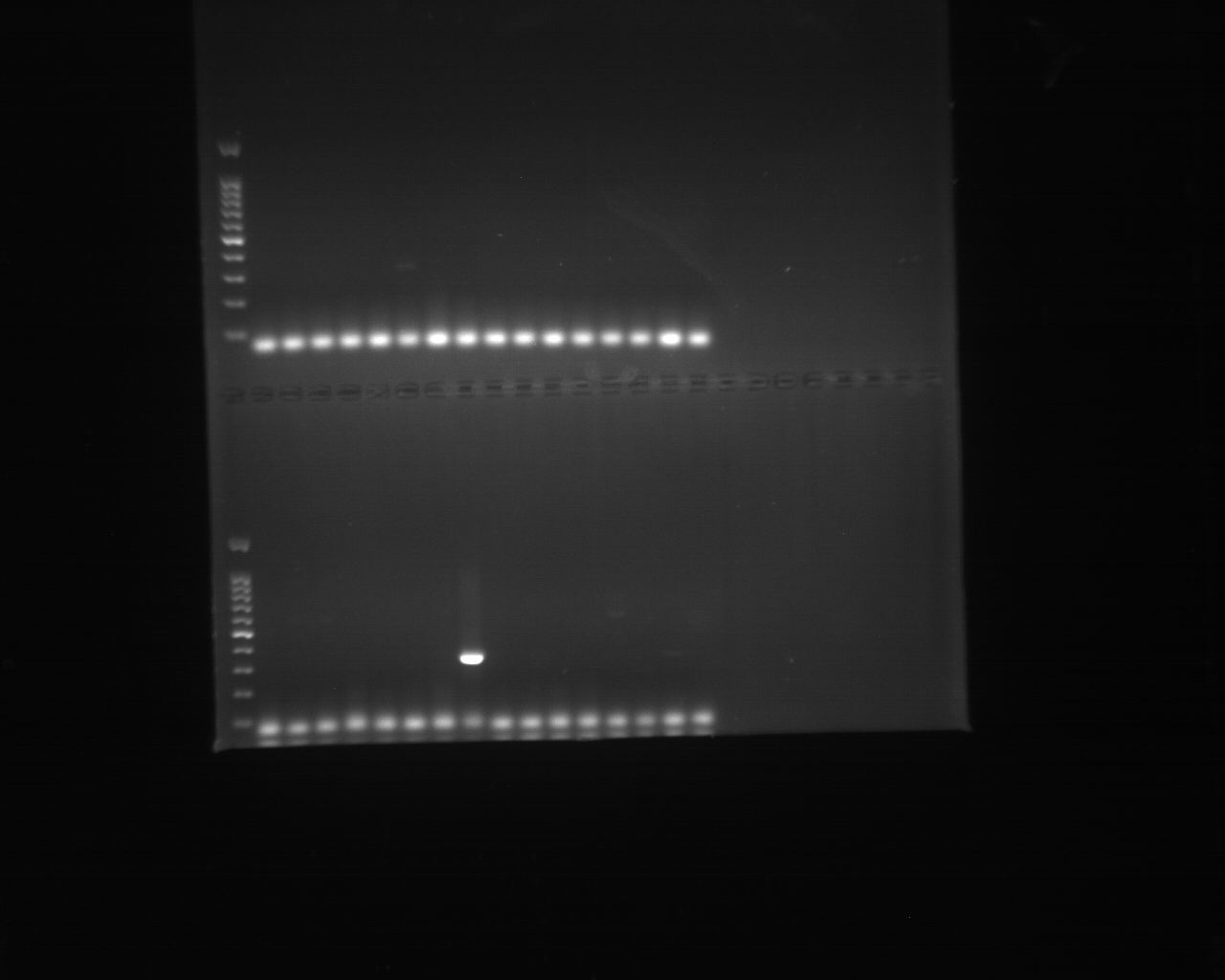

Supplement: Figure 3—source data 2. [file elife-89532-fig3-data2.zip › Figure 3-Source Data 2/E3/Row image/Ga3 Ga4 .Tif]

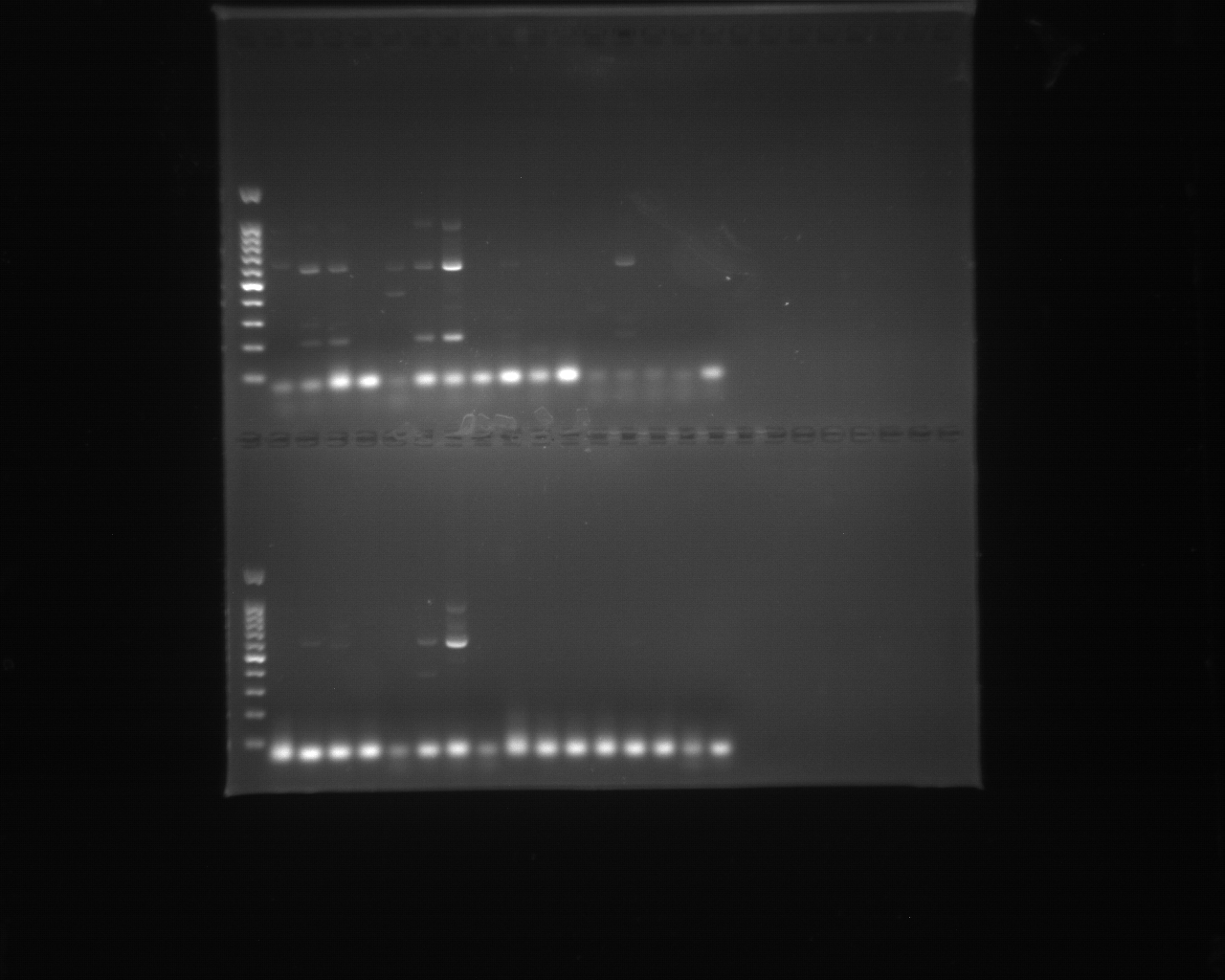

Supplement: Figure 3—source data 2. [file elife-89532-fig3-data2.zip › Figure 3-Source Data 2/E3/Row image/Ga5 Ga6 .Tif]

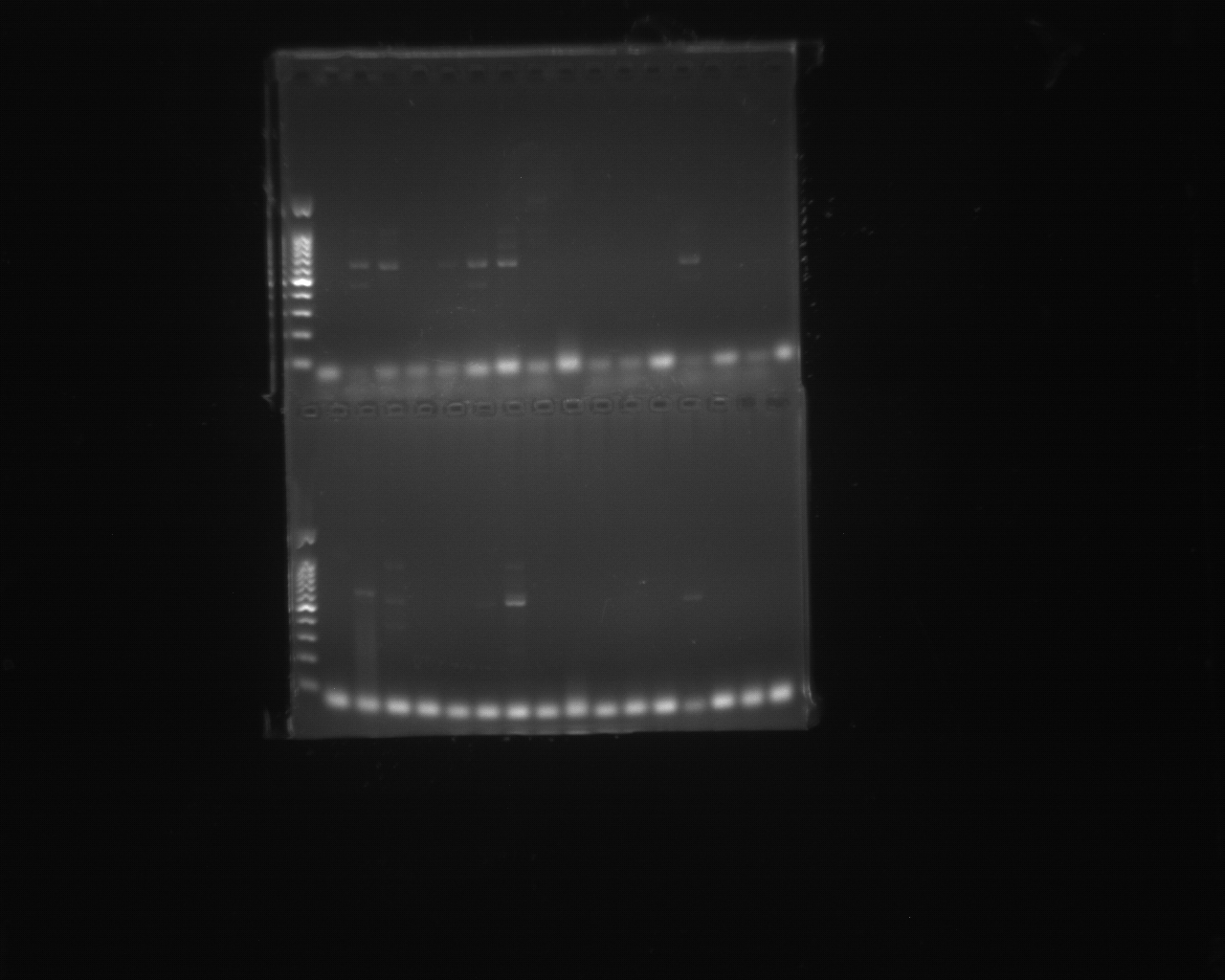

Supplement: Figure 3—source data 2. [file elife-89532-fig3-data2.zip › Figure 3-Source Data 2/E3/Row image/Ga7 Ga9 .Tif]

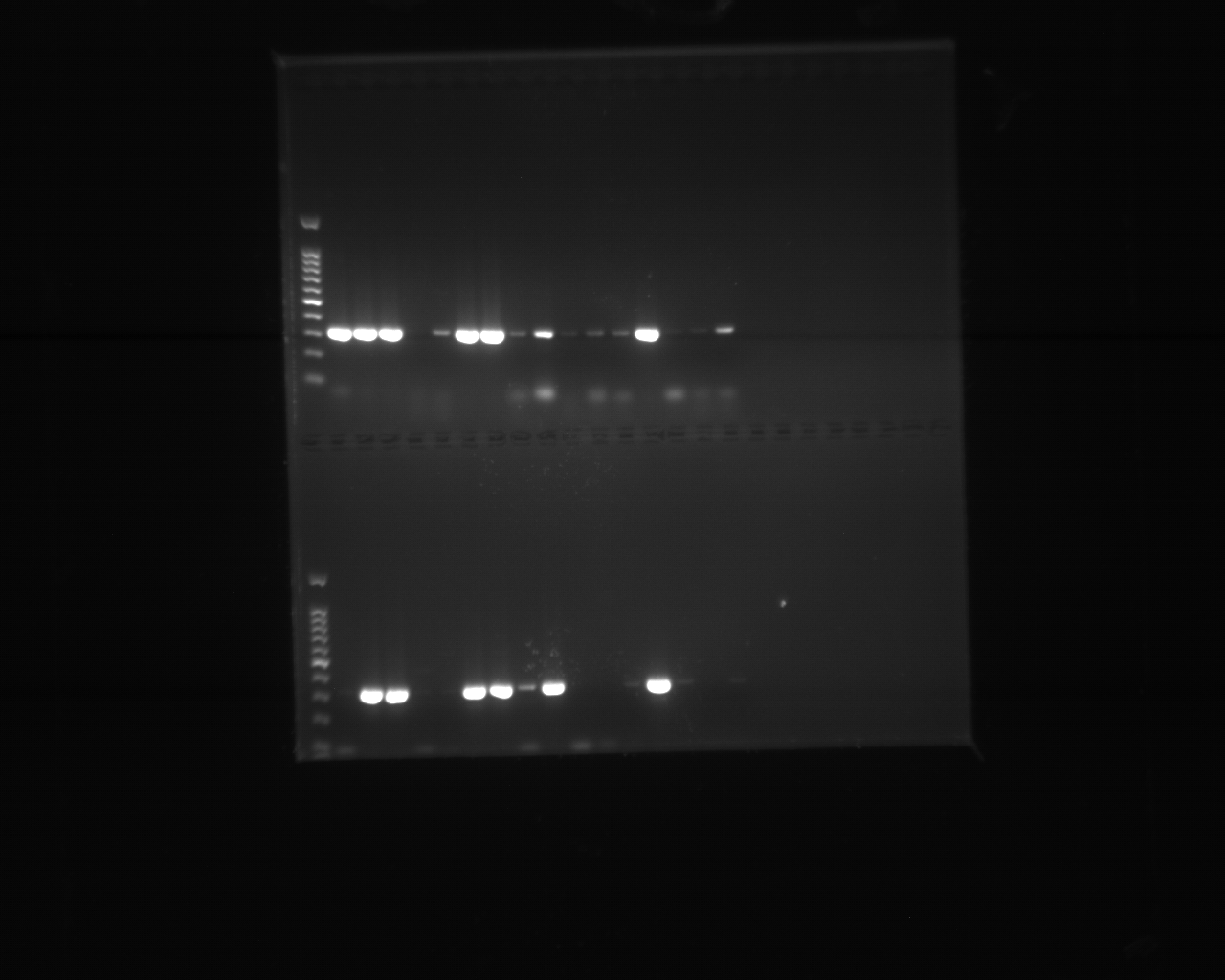

Supplement: Figure 3—source data 2. [file elife-89532-fig3-data2.zip › Figure 3-Source Data 2/E3/Row image/GA8 GA10 .Tif]

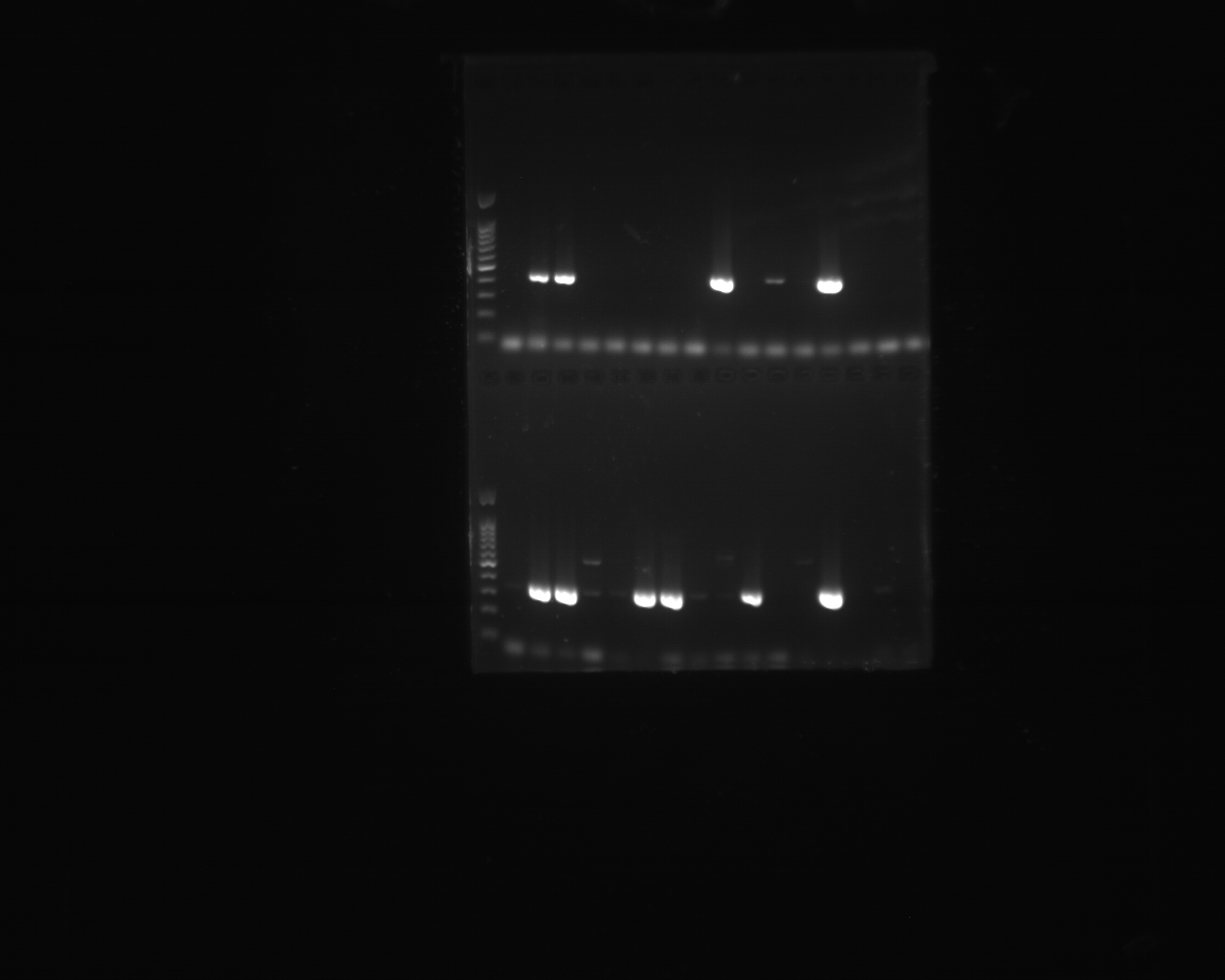

Supplement: Figure 3—source data 2. [file elife-89532-fig3-data2.zip › Figure 3-Source Data 2/E3/Row image/GB1 GB2 .Tif]

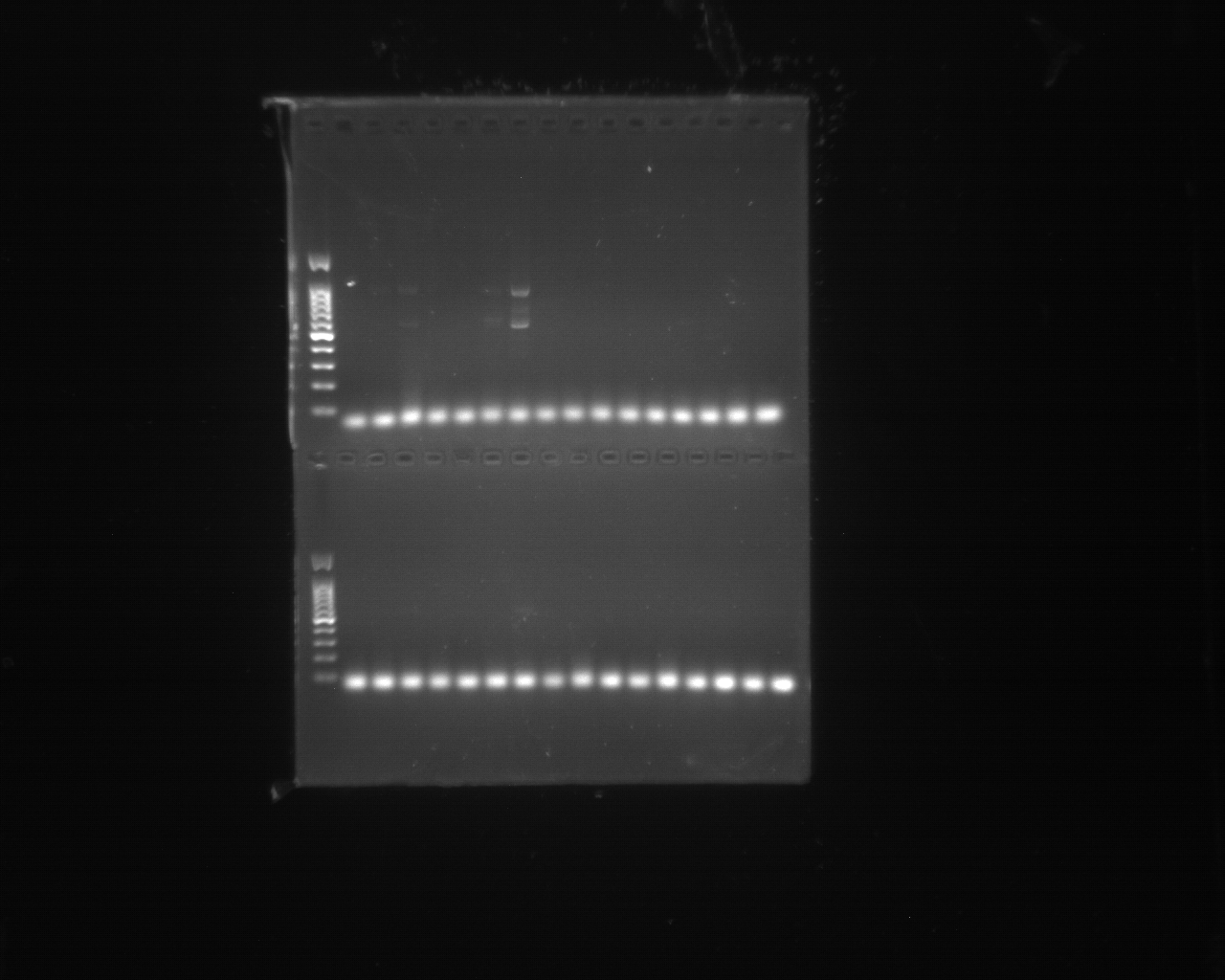

Supplement: Figure 3—source data 2. [file elife-89532-fig3-data2.zip › Figure 3-Source Data 2/E3/Row image/Gb4 Gb5 .Tif]

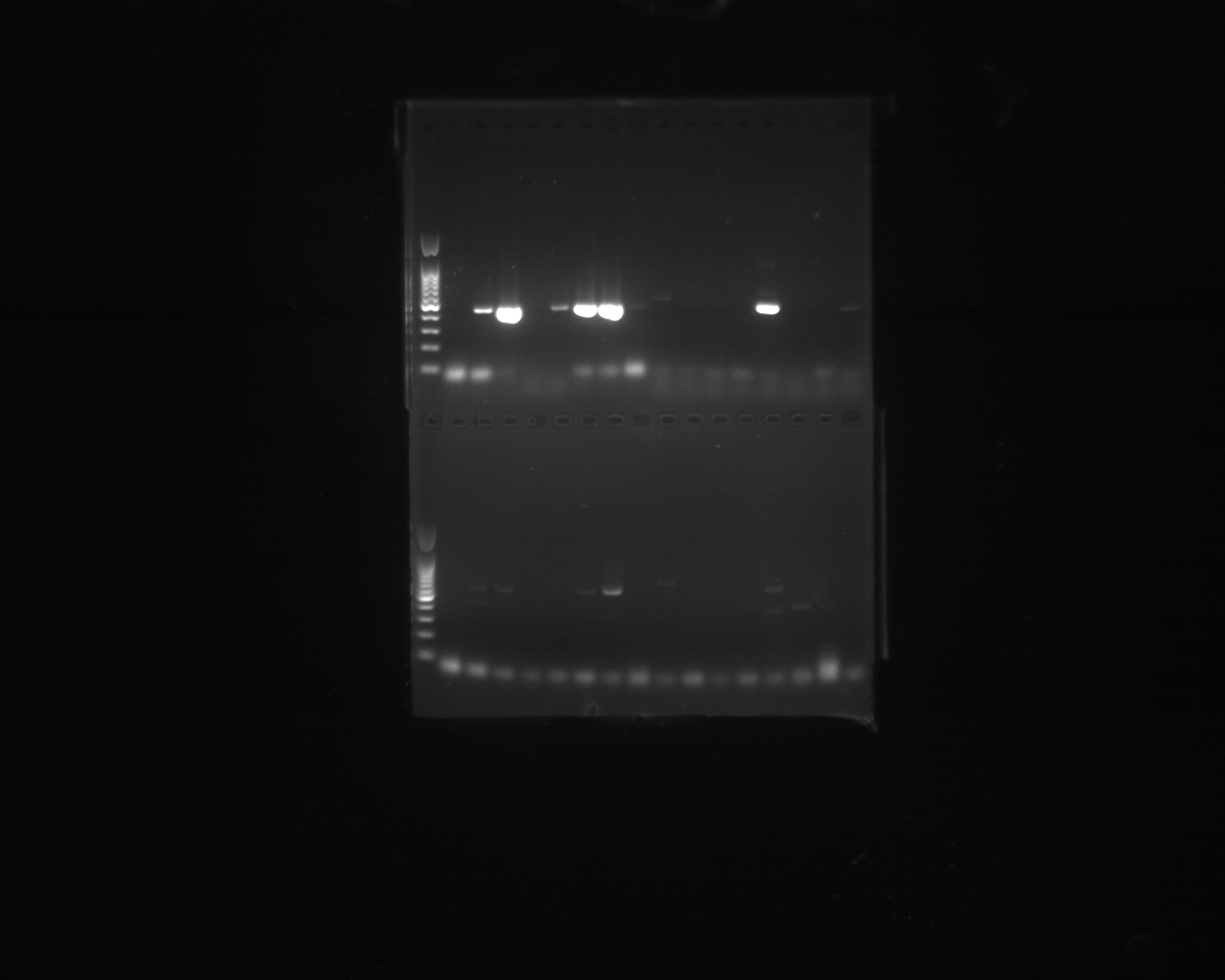

Supplement: Figure 3—source data 2. [file elife-89532-fig3-data2.zip › Figure 3-Source Data 2/E3/Row image/GB6 GB7 .Tif]

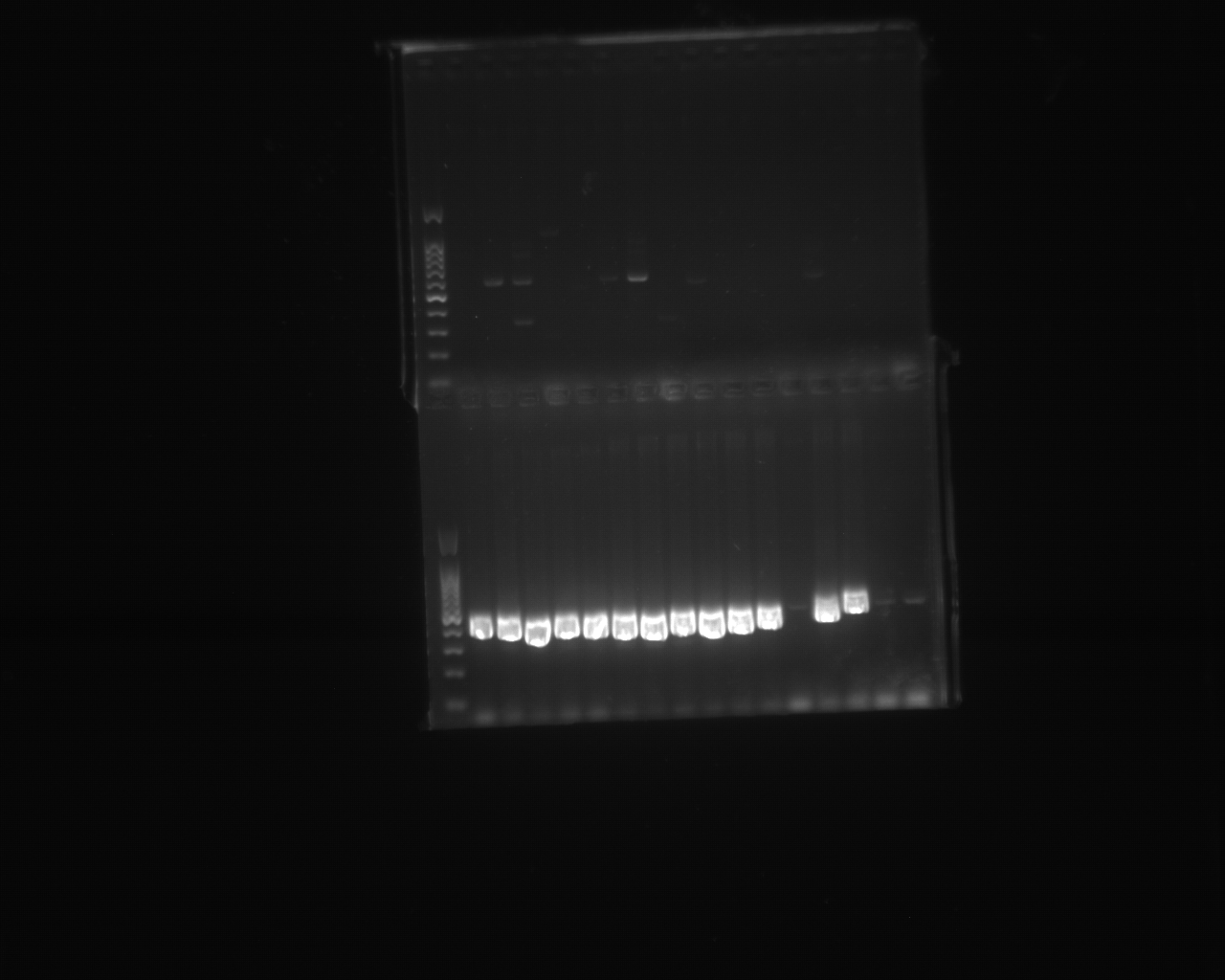

Supplement: Figure 3—source data 2. [file elife-89532-fig3-data2.zip › Figure 3-Source Data 2/E3/Row image/Gb8 actin (2).Tif]

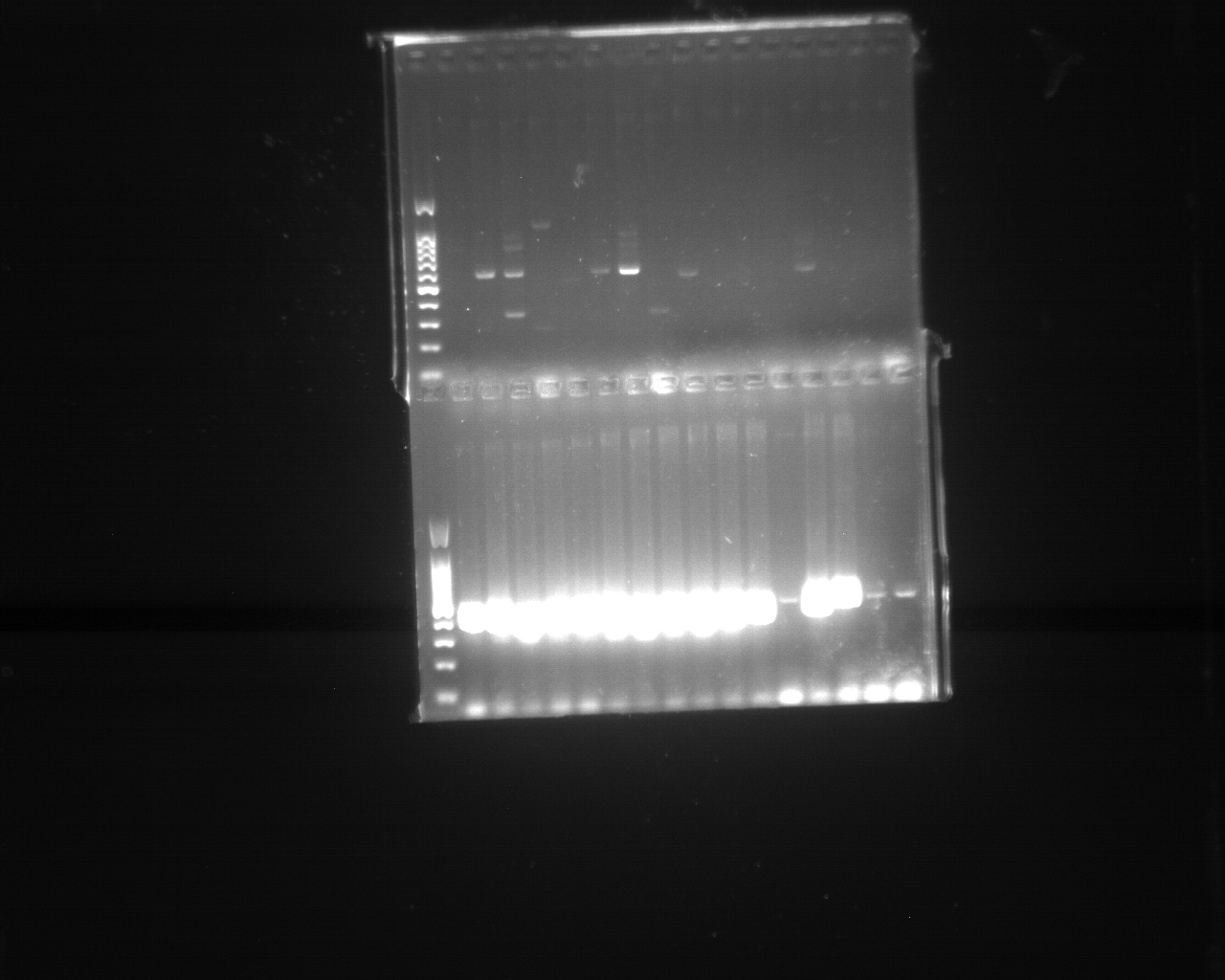

Supplement: Figure 3—source data 2. [file elife-89532-fig3-data2.zip › Figure 3-Source Data 2/E3/Row image/Gb8 actin .Tif]

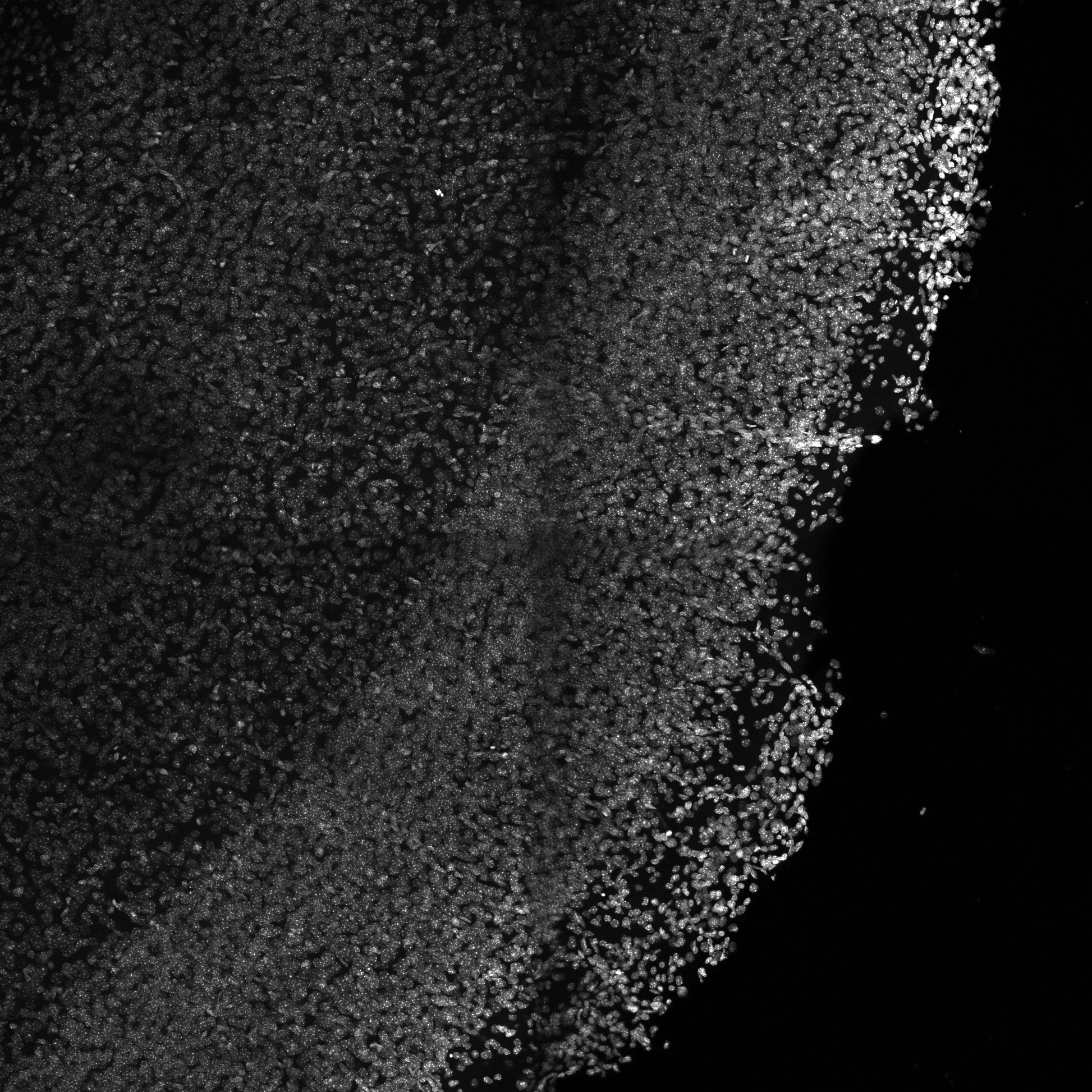

Supplement: Figure 3—figure supplement 1—source data 1. [file elife-89532-fig3-figsupp1-data1.zip › Figure 3-Figure Supplement1-Source Data1/Row image/1.tif]

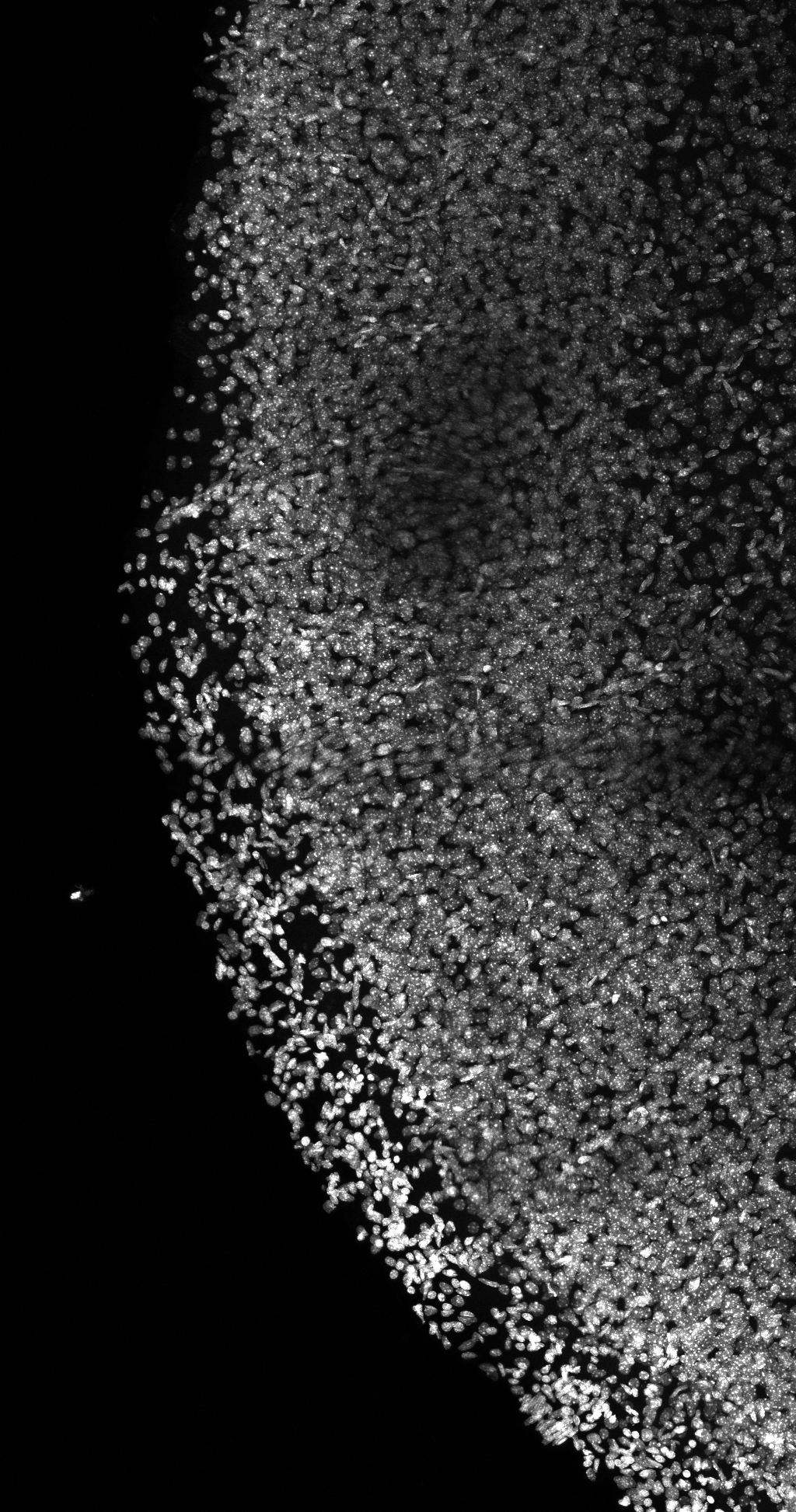

Supplement: Figure 3—figure supplement 1—source data 1. [file elife-89532-fig3-figsupp1-data1.zip › Figure 3-Figure Supplement1-Source Data1/Row image/2.tif]

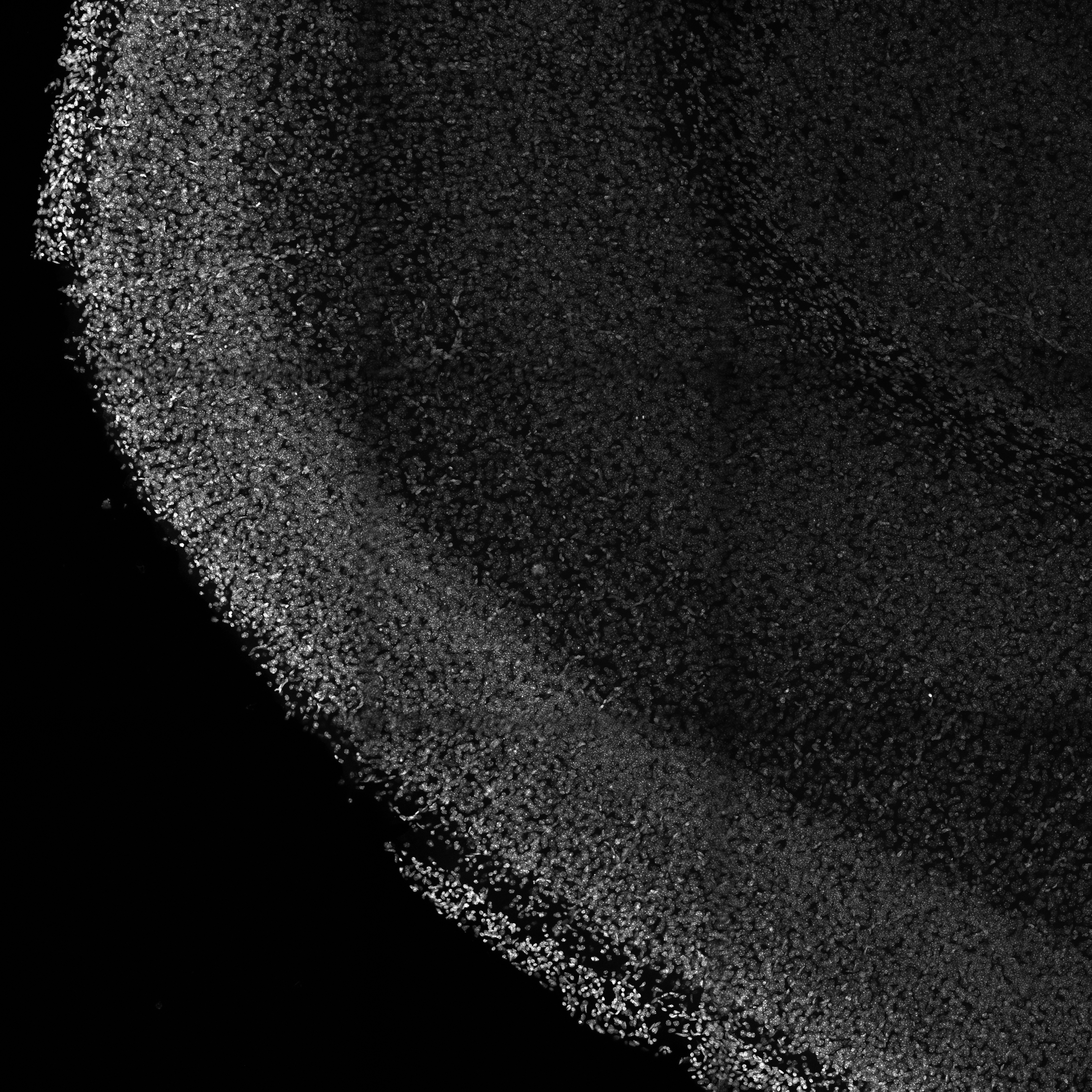

Supplement: Figure 3—figure supplement 1—source data 1. [file elife-89532-fig3-figsupp1-data1.zip › Figure 3-Figure Supplement1-Source Data1/Row image/3.tif]

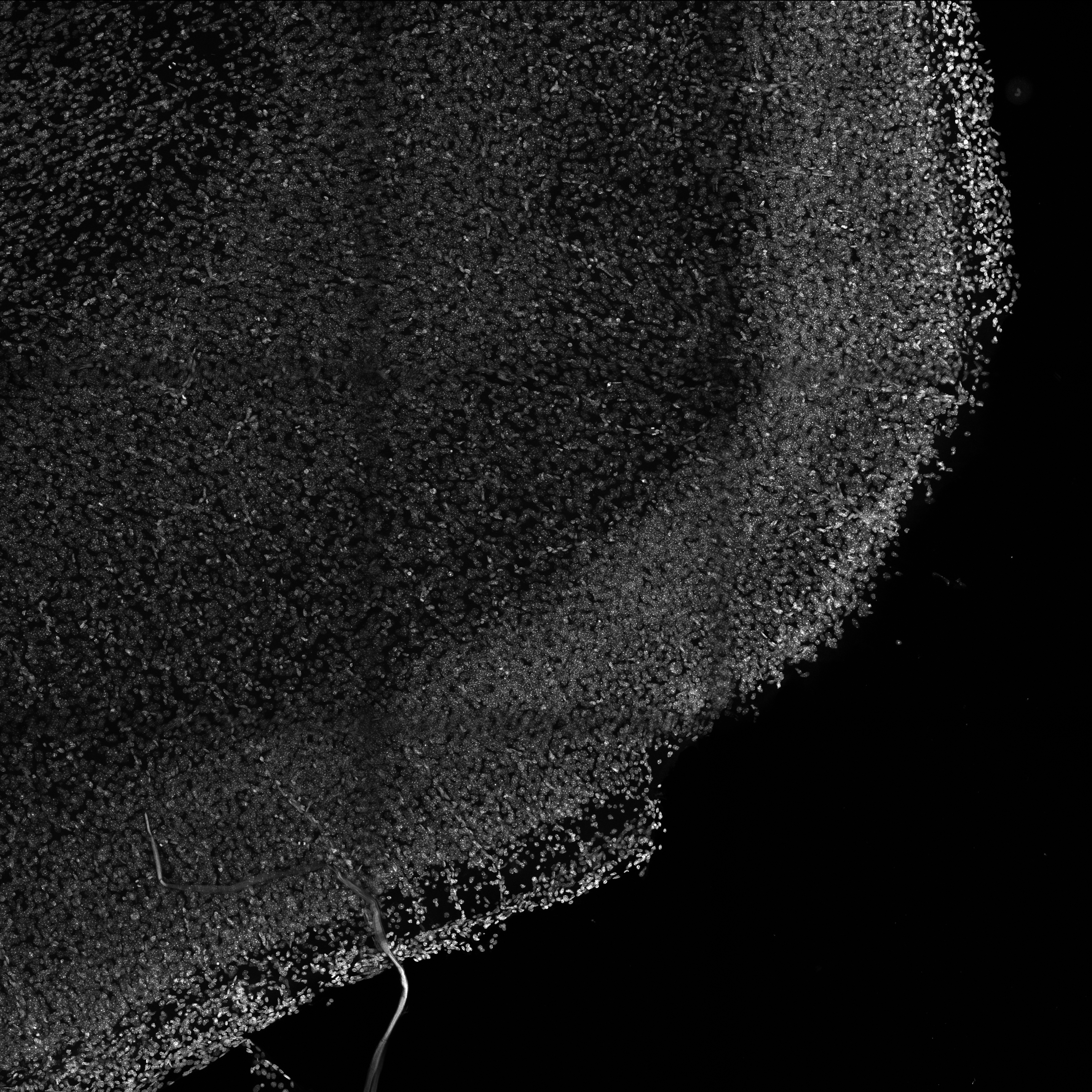

Supplement: Figure 3—figure supplement 1—source data 1. [file elife-89532-fig3-figsupp1-data1.zip › Figure 3-Figure Supplement1-Source Data1/Row image/4.tif]
