# Supplementary material for: Combinatorial expression of γ-protocadherins regulates synaptic connectivity in the mouse neocortex
Source: eLife. 2024 Mar 12;12:RP89532. doi: 10.7554/eLife.89532 (PMC10932546; doi:10.7554/eLife.89532)
Supplement: Figure 3—source data 2. [file elife-89532-fig3-data2.zip › Figure 3-Source Data 2/legend for Figure 3-Source Data2.docx]

We performed 3 times of experiments (named E1, E2 and E3).

We selected the cells collected in 2h after sections (cell 1-cell 9 in all experiments) to reduce the influence of RNA degradation.

We used pipette internal solutions and Rnase free water as negative controls.

Slice incubation solutions was used to measure external RNA pollutions.

Experiment 1 2019.7.7 **(Left panel)**

ga1- ga12, gb1 gb2,

Lane 1:100bp DNA ladder

Lane 2-21: cell1-cell20(cell1-cell9 was kept for analysis as 1-9)

Lane 22: slice incubation solutions

Lane 23: pipette internal solutions (Rnase free aCSF)

Lane 24: Rnase free water

gb4,gb5,gb8,actin:

Lane 1-12: cell1-cell12

Lane 13:100bp DNA ladder

Lane 14-21:cell13-cell20(cell1-cell9 was kept for analysis as 1-9)

Lane 22: slice incubation solutions

Lane 23: pipette internal solutions (Rnase free aCSF)

Lane 24: Rnase free water

The figures were named by the target gene name (up-down)

The figures contains actin were collected twice in different contrast.

Actin was confirmed again with the same order of PCR products.

Experiment 2 2019.7.28 **(Middle panel)**

Lane 1: 100bp DNA ladder

Lane 2-15: cell 1-14(cell1-cell9 was kept for analysis as 10-18)

Lane 16: pipette internal solutions (Rnase free aCSF)

Lane 17: Rnase free water

The figures were named by the target gene name (up-down)

The figures contains actin were collected twice in different contrast.

Actin was confirmed twice with the same order of PCR products.

Experiment 3 2019.8.4 **(Right panel)**

Lane 1: 100bp DNA ladder

Lane 2-14: cell 1-13(cell2-cell9 was kept for analysis as 19-26)

Lane 15: slice incubation solutions

Lane 16: pipette internal solutions (Rnase free aCSF)

Lane 17: Rnase free water

Cell1 was skipped because that cell was not completely collected into the pipette.

The figures were named by the target gene name (up-down)

The figures contains actin were collected twice in different contrast.

Actin was confirmed again with the same order of PCR products.

We misaligned the actin line in the previous figure in E3 (**Figure 3-Figure supplement 2D, right panel**). Now we corrected this mistake.

Specific amplification product length(bps):

Pcdhg a1=354

Pcdhg a2=353

Pcdhg a3=321

Pcdhg a4=318

Pcdhg a5=321

Pcdhg a6=532

Pcdhg a7=538

Pcdhg a8=315

Pcdhg a9=344

Pcdhg a10=328

Pcdhg a11=339

Pcdhg a12=703

Pcdhg b1=440

Pcdhg b2=349

Pcdhg b4=363

Pcdhg b5=449

Pcdhg b6=448

Pcdhg b7=448

Pcdhg b8=349
